# Elucidation of the glycosylation steps during biosynthesis of antitumor macrolides PM100117 and PM100118 and engineering for novel derivatives

**DOI:** 10.1186/s12934-016-0591-7

**Published:** 2016-11-09

**Authors:** Raúl García Salcedo, Carlos Olano, Rogelio Fernández, Alfredo F. Braña, Carmen Méndez, Fernando de la Calle, José A. Salas

**Affiliations:** 1 Departamento de Biología Funcional e Instituto Universitario de Oncología del Principado de Asturias (I.U.O.P.A), Universidad de Oviedo, Asturias, 33006 Oviedo, Spain; 2 Drug Discovery Area, PharmaMar S.A., Avda. de los Reyes, Colmenar Viejo, 128770 Madrid, Spain

**Keywords:** Antitumor, Deoxysugar, Glycosyltranferase, Structural analogue

## Abstract

**Background:**

Antitumor compounds PM100117 and PM100118 are glycosylated polyketides derived from the marine actinobacteria *Streptomyces caniferus* GUA-06-05-006A. The organization and characterization of the PM100117/18 biosynthesis gene cluster has been recently reported.

**Results:**

Based on the preceding information and new genetic engineering data, we have outlined the pathway by which PM100117/18 are glycosylated. Furthermore, these genetic engineering experiments have allowed the generation of novel PM100117/18 analogues. Deletion of putative glycosyltranferase genes and additional genes presumably involved in late biosynthesis steps of the three 2,6-dideoxysugars appended to the PM100117/18 polyketide skeleton, resulted in the generation of a series of intermediates and novel derivatives.

**Conclusions:**

Isolation and identification of the novel compounds constitutes an important contribution to our knowledge on PM100117/18 glycosylation, and set the basis for further characterization of specific enzymatic reactions, additional genetic engineering and combinatorial biosynthesis approaches.

**Electronic supplementary material:**

The online version of this article (doi:10.1186/s12934-016-0591-7) contains supplementary material, which is available to authorized users.

## Background

Natural products (NPs) are an invaluable source of biologically active drugs with therapeutic significance [1, 2]. A considerable portion of these products present as part of their chemical structure deoxysugar (DOH) moieties, mainly 6-deoxyhexoses (6DOH) and 2,6-dideoxyhexoses (2,6DOH) [3, 4]. Many studies aimed to unravel the structure–activity relationship of numerous NPs have shown the relevant and diverse functional roles played by DOHs on a wide range of biological activities, including antibiotic, antifungal, antitumor and antiparasitic [5–8]. In accordance with these important roles, removal of DOH moieties might lead to various degrees of reduction in activity, ranging from a complete activity loss to the retention of certain activity level as the glycosidic fraction is eliminated [4].

Glycobiology research has led to discover and delineate the biosynthesis pathway of many natural sugars [9]. As a result, a core set of enzymatic activities has been found to synthesize the diverse sugar structures observed in nature. Various common enzyme reactions, which include deoxygenations, transaminations, ketoreductions, *C*-, *N*- or *O*-methylations, epimerizations, and less frequently oxygenations, are required to generate the surprisingly high structural diversity of natural DOHs [3, 9]. The complete enzyme set required for DOH biosynthesis is, in most cases, encoded in the biosynthesis gene cluster of the respective NP. Therefore, because the great majority of sugars present in bioactive NPs are DOHs, identification of clusters containing genes coding for these activities may constitute a useful strategy for the discovery of clinical attractive drugs [10, 11]. Some of these enzymes have been thoroughly characterized in vitro [12–14] and in vivo [15] in multiple attempts to modify sugar structures and alter the glycosylation pattern of NP. Upon DOHs biosynthesis, transfer onto specific positions of the corresponding aglycone is controlled by glycosyltransferase (GT) enzymes. These enzymes require an activated DOH donor, typically a NDP or NMP-DOH, for transfer of the glycosyl residue to the acceptor molecule [16]. In this way, a staggering diversity of sugars can be transferred to a no less structurally diverse array of acceptor molecules, including polyketide, nonribosomal peptide, indolocarbazole and aminocoumarin aglyca among many others, by the various hundreds of putative GTs described in databases as involved in NPs biosynthesis [16] (http://www.cazy.org/GlycosylTransferases.html). Most GTs catalyze *O*-glycosidic bond formation between the sugar and its natural aglycone. Other linkages such as *C*–*C* [17] or *C*–*N* [18] glycosidic bonds are relatively unusual. Substrate flexibility is an interesting feature of some sugar biosynthesis and GT enzymes. This is especially remarkable in GTs which can show different degrees of promiscuity towards the acceptor and/or the donor molecules [3, 8, 19, 20]. Similarly, other GTs possesses the unusual ability to transfer sugars to different positions of the same aglycone sometimes using different type of linkages [21, 22]. GT flexibility is a key aspect for the success of many glycosylation pattern engineering strategies aimed to afford and develop new NPs. Deletion and heterologous expression of DOH biosynthesis and/or GTs genes have allowed the generation of a handful of interesting derivatives from structurally diverse NPs, such as erythromycin [23], methymicin/pikromycin [24], daunorubicin/doxorubicin [25], rebeccamycin [26] or elloramycin [27–29], among others. In some cases, novel derivatives resulting from these strategies show an enhanced bioactivity and/or improved pharmacological properties. Relevant examples of glycosylation-pattern modifications leading to these improvements are the aureolic acids demycarosyl-3D-β-d-digitoxosyl-mithramycin SK, demycarosyl-mithramycin SDK and demycarosyl-3D-β-d-digitoxosyl-mithramycin SDK, which derived from the clinically used anticancer agent mithramycin by combinatorial biosynthesis in *Streptomyces argillaceus* [30].

**Fig. 1:**
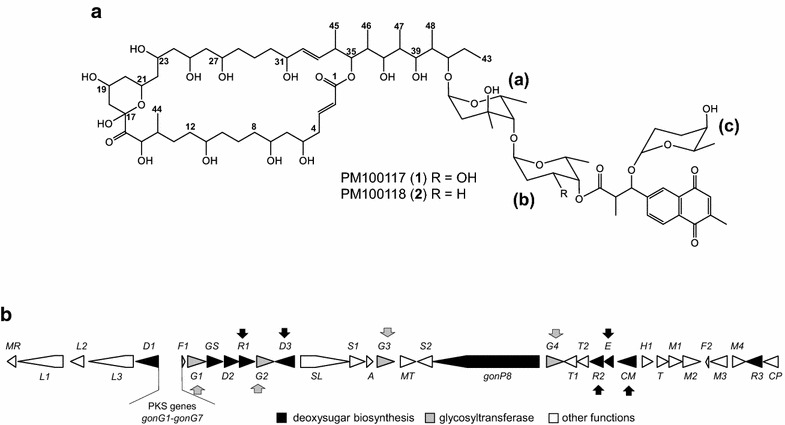
PM100117 and PM100118 2,6-DOH moieties and biosynthesis gene cluster. **a** PM100117/18 chemical structure. DOH moieties are indicated: *a* l-axenose *b* l-rhodinose (R = H) or 2-deoxi-l-fucose (R = OH) *c* l-rhodinose. **b** Genetic organization of PM100117/18 biosynthesis gene cluster showing genes involved in 6DOH biosynthesis (*black arrows*) and transfer (*grey arrows*)

PM100117 and PM100118 are glycosylated macrolides produced by the marine actinobacteria *Streptomyces caniferus* GUA-06-05-006A [31, 32]. The glycosylation pattern of PM100117/18 consists of three 2,6DOHs (l-axenose, 2-deoxy-l-fucose and l-rhodinose), which along with a naphthoquinone chromophore form a side chain attached to the PM100117/18 polyketide skeleton (Fig. 1a). The remarkable antitumor activity of PM100117/18, and the generation of structural analogues with improved bioactivity recently reported [32], prompted us to undertake the engineering of these compounds looking for more derivatives. Here, we describe the generation of seven novel analogues by a glycoengineering approach consisting in the deletion of PM100117/18 biosynthetic genes previously characterized in silico as coding for putative GTs and key sugar biosynthetic enzymes (Fig. 1b). In addition, the outcome of these genetic engineering manipulations provided valuable information on the PM100117/18 glycosylation steps.

## Results

### Deletion of 2,6-dideoxysugar biosynthesis genes

**Fig. 2:**
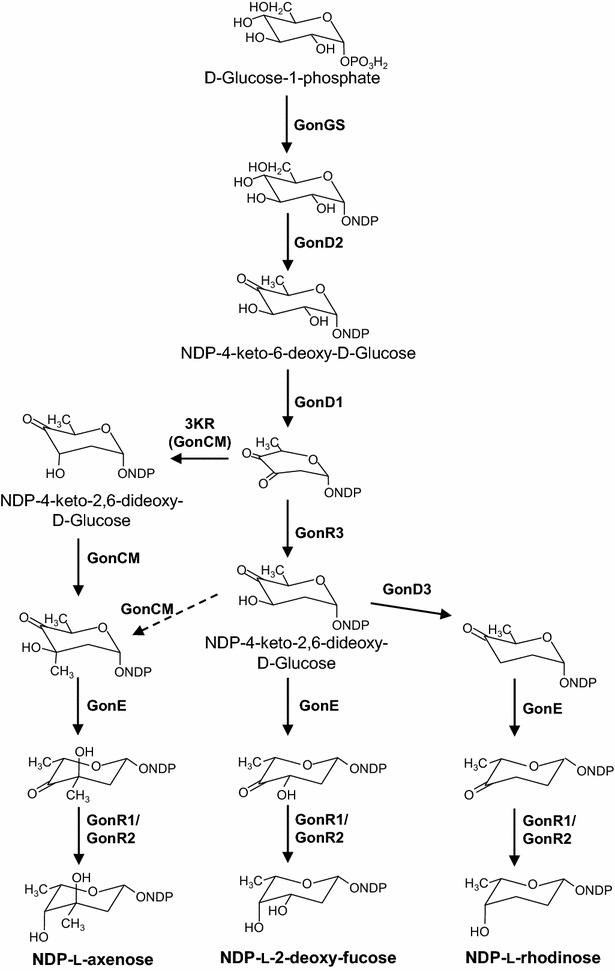
Proposed biosynthesis pathway of PM100117 and PM100118 2,6-DOH moieties. *3KR*: ketoreductase

The PM100117/18 gene cluster encodes most of the putative functions required to catalyze the biosynthesis of the three 2,6DOH moieties appended to the PM100117/18 macrolactone ring (Fig. 1). From these activities, as deduced in silico (Additional file 1), biosynthesis of l-axenose, 2-deoxy-l-fucose and l-rhodinose can be envisaged to occur as depicted in Fig. 2. Early biosynthesis steps of 6DOHs involves intermediacy of nucleoside diphosphate (NDP)-4-keto-6DOH, which is synthesized from a hexose-1-phospate, most possibly d-glucose, in two reaction steps catalyzed by a NDP-d-hexose synthase (GonGS) and a NDP-d-hexose-4,6-dehydratase (GonD2), respectively. To achieve, 2-deoxy-l-fucose and l-rhodinose, NDP-4-keto-6DOH could be converted into a NDP-4-keto-2,6DOH intermediate with an equatorial hydroxyl group at carbon C3, by the sequential activity of NDP-d-hexose-2,3-dehydratase (GonD1) and NDP-d-hexose-3-ketoreductase (GonR3), in accordance with the high resemblance of GonR3 to other 3-ketoreductases that render this configuration (Additional file 1). Similarly, l-axenose biosynthesis would involve a NDP-4-keto-2,6DOH intermediate with a hydroxyl group at carbon C3 in axial stereo configuration, however the corresponding NDP-d-hexose-3-ketoreductase is not present in the PM100117/18 gene cluster. Later steps towards PM100117/18 DOHs biosynthesis from this key 2,6DOH intermediate might involve four reaction types outlined in Fig. 2, which include *C*-methylation at C3 (GonCM), 3-deoxygenation (GonD3), 5- or 3,5-epimerization (GonE) and 4-ketoreduction (GonR1/GonR2).

**Fig. 3:**
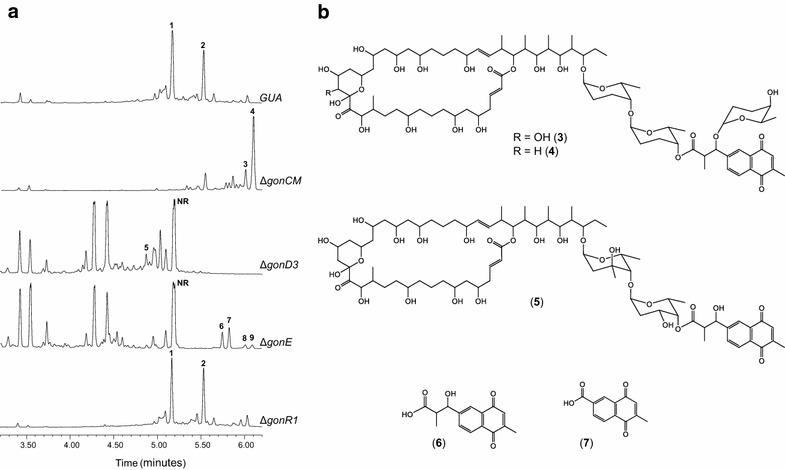
Production of PM100117 (*1*) and PM100118 (*2*) derivatives* 3*–*9* in 2,6-DOH biosynthesis gene mutant strains. **a** UPLC analysis (254 nm) of metabolite production in *S. caniferus* GUA-06-05-006A wild-type and Δ*gonCM*, Δ*gonD3*, Δ*gonE* and Δ*gonR1* deletion mutants. *NR*: peak non-related to PM100117/18. **b** Chemical structure of various PM100117/18 analogues

Herein, we sought to generate PM100117/18 structural analogues by manipulating the biosynthesis of their pendant 2,6DOHs. For that purpose we generated the mutant strains *ΔgonCM*, *ΔgonD3*, *ΔgonE*, and *ΔgonR1*, in which genes involved in late steps of 2,6DOH biosynthesis, *gonCM*, *gonD3*, *gonE*, and *gonR1*, respectively, were deleted. To detect the biosynthesis of PM100117/18 derivatives, whole-culture extracts from these strains were analyzed by UPLC (Fig. 3) and HPLC/MS. UPLC chromatograms from strains Δ*gonCM*, Δ*gonD3* and Δ*gonE* showed suppression of PM100117 (1, UPLC R_*t*_ = 5.182 min, *m/z* 1601.9 [*M* + Na]^+^) and PM100118 (2, UPLC R_*t*_ = 5.536 min, *m/z* 1585.9 [*M* + Na]^+^) biosynthesis, and the appearance of several peaks corresponding to potential analogues (Fig. 3a). Mass assessments by HPLC/MS suggested that the novel compounds detected in Δ*gonCM* are compatible with PM100117/18 analogues in which the first sugar moiety, l-axenose, has been substituted for either 2-deoxy-l-fucose or l-rhodinose. This was confirmed by elucidating the chemical structure of products 3 [UPLC R_*t*_ = 6.035 min, *m/z* 1571.9 (*M* + Na)^+^] and 4 [UPLC R_*t*_ = 6.102 min, *m/z* 1555.9 (*M* + Na)^+^] by NMR, showing that both are PM100118 derivatives with an l-rhodinose unit in place of l-axenose as first sugar moiety (Fig. 3b and Additional file 2: Fig. S1 and Table S1). The presence of a hydroxyl group at C18 of the polyketide ring in 3 (absent in 4 and also in 1 and 2) suggests that compound 3 might be a shunt product of PM100117/18 biosynthesis after undergoing a hydroxylation reaction at this position. These results are consistent with the involvement of GonCM in l-axenose biosynthesis (Fig. 2). Likewise, compound 5 [UPLC R_*t*_ = 4.941 min, *m/z* 1487.8 (*M* + Na)^+^], produced by strain Δ*gonD3* (Fig. 3a), was also purified and subjected to NMR analysis (Additional file 2: Fig. S1 and Table S2), confirming that, as expected from the hypothetical 2,6DOH biosynthetic pathways outlined in Fig. 2, this product structurally derives from PM100117 by lacking the third sugar moiety, l-rhodinose. The estimated molecular mass for compounds 6 [UPLC R_*t*_ = 5.721 min, *m/z* 297 (*M* + Na)^+^] and 7 [UPLC R_*t*_ = 5.796 min, *m/z* 239 (*M* + Na)^+^] from strain Δ*gonE* was strikingly low in comparison to that of the parental products, suggesting that these compounds might not be related to PM100117/18 biosynthesis. However, structure elucidation (Additional file 2: Fig. S1 and Table S3) revealed that 6 corresponds to the naphthoquinone chromophore, while 7 is the precursor of 6 to which a propionate side chain is incorporated (Fig. 3b). Masses displayed by compounds 8 [UPLC R_*t*_ = 6.012 min, *m/z* 1457.8 (*M* + Na)^+^] and 9 [UPLC R_*t*_ = 6.098 min, *m/z* 1441.8 (*M* + Na)^+^], fitted with PM100117 lacking both l-rhodinose as third sugar moiety and oxygenations at C16 and/or C17. Nonetheless, neither these structural features nor the configuration of the sugar moieties presumably present in 8 and 9 have been further confirmed because the low production level and high instability of these products, which made impossible the purification of quantities suitable for NMR analysis. On the other hand, mutant Δ*gonR1* produced PM100117/18 at similar levels to those observed in wild type *S. caniferus* GUA-06-05-006A (Fig. 3a), indicating that this gene is not essential for PM100117/18 2,6DOHs biosynthesis. It is important to note the presence in the PM100117/18 gene cluster of an additional putative NDP-4-keto-6-deoxyhexose reductase gene, *gonR2* (Fig. 1b). We set to determine whether this gene codes for the 4-ketoreductase activity presumably involved in the final biosynthesis step of the three PM100117/18 2,6DOHs (Fig. 2). Nevertheless, after recurrent attempts, we fail to produce a *gonR2* deletion mutant. As an alternative approach, *gonR2* was cloned under the control of the ermE*p promoter to produce pC*gonR2*, which was introduced in *S. caniferus* GUA-06-05-006A. The metabolic profile of the resultant strain, OE*gonR2*, was then analyzed by UPLC, showing no differences with respect to wild type strain, GUA-pSETH, carrying the respective empty plasmid (Additional file 3: Fig. S2).

### Deletion of glycosyltransferase genes

**Fig. 4:**
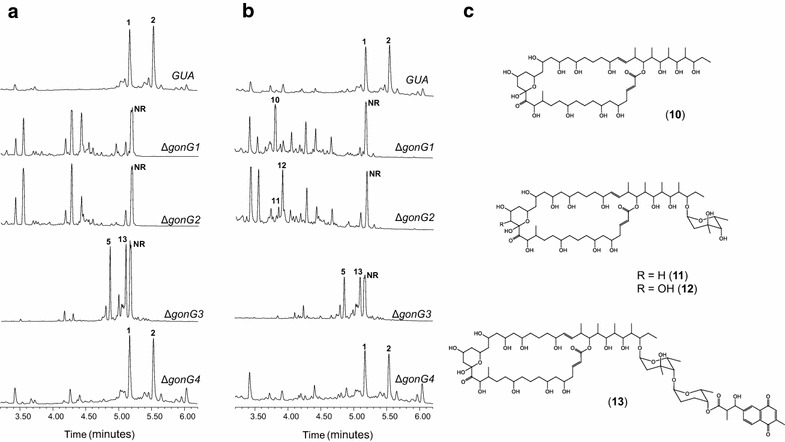
Production of PM100117 (*1*) and PM100118 (*2*) biosynthesis intermediates* 10*–*13* in glycosyltransferase gene mutant strains. UPLC analysis at 254 (**a**) and 216 nm (**b**) wavelength of metabolite production in *S. caniferus* GUA-06-05-006A wild-type and Δ*gonG1*, Δ*gonG2*, Δ*gonG3*and Δ*gonG4* deletion mutants. *NR*: peak non-related to PM100117/18. **c** Chemical structure of various PM100117/18 analogues

The PM100117/18 biosynthesis gene cluster contains four ORFs (*gonG1*, *gonG2*, *gonG3* and *gonG4*) coding for putative GT proteins [32], each of which might be involved in transfer of at least one DOH moiety to a defined position of PM100117/18. As additional route to produce PM100117/18 structural analogues, we pursued to manipulate 2,6DOH transfer to the PM100117/18 macrolactone aglycone by independently deleting these putative GT genes. Thus, we produced mutant strains Δ*gonG1*, Δ*gonG2*, Δ*gonG3* and Δ*gonG4*. Metabolite production of these strains was examined as described above. It is known that loss of the naphthoquinone chromophore unit causes a shift in the PM100117/18 maximum absorption wavelength from 254 to 216 nm [32]. Thereby, since deletion of GT genes could lead to glycosylation pattern changes involving loss of this structural element, screening for novel derivatives entailed the analysis of chromatograms at both wavelengths. Deletion of *gonG1* or *gonG2* abolished PM100117/18 production (Fig. 4a) and induced the accumulation of various potential analogues with a maximum absorption wavelength at 216 nm, hence lacking the naphthoquinone moiety (Fig. 4b). HPLC/MS analysis showed that novel compounds produced by strain Δ*gonG1* possess masses corresponding to different variations of the PM100117/18 non-glycosylated aglycone, while masses of those produced by mutant strain Δ*gonG2* matched putative PM100117/18 analogues lacking the second 2,6DOH moiety. Structural elucidation by NMR (Additional file 2: Fig. S1 and Table S4 and S5) revealed that compound 10 (UPLC R_*t*_ = 3.808 min, *m/z* 957.6 [*M* + Na]^+^) from Δ*gonG1* indeed corresponded to the PM100117/18 aglycone, while 11 (UPLC R_*t*_ = 3.842 min, *m/z* 1102.4 [*M* + Na]^+^) and 12 (UPLC R_*t*_ = 3.905 min, *m/z* 1117.7 [*M* + Na]^+^) from strain Δ*gonG2* were PM100117/18 derivatives retaining only the first sugar moiety, l-axenose (Fig. 4c). These results implicate the GTs GonG1 and GonG2 in transfer of the first and second 2,6DOH moiety, respectively, during PM100117/18 biosynthesis. UPLC and HPLC/MS analysis of Δ*gonG3* metabolite biosynthesis showed the production of derivative 5 (Fig. 4b) as well as an additional compound, 13 (UPLC R_*t*_ = 5.143 min, *m/z* 1471.8 [*M* + Na]^+^), with absorption (254 nm) and mass spectra compatible with a PM100118 derivative lacking the third sugar moiety. Confirmation of this structure by NMR analysis (Fig. 4c; Additional file 2) endorsed the involvement of the GT GonG3 in transfer of the third 2,6DOH, l-rhodinose. Lastly, deletion of *gonG4* did not show any effect either on PM100117/18 biosynthesis, which were produced at comparable levels to those observed in the wild type strain *S. caniferus* GUA-06-05-006A, or on the accumulation of novel PM100117/18 derivatives (Fig. 4a, b). The study of the possible role of this gene in PM100117/18 biosynthesis is addressed in section below.

**Fig. 5:**
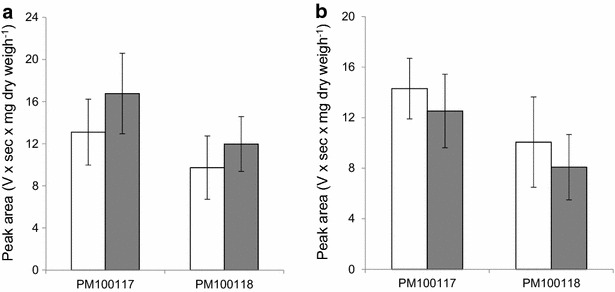
Analysis of GonG4 functional role on PM100117 and PM100118 biosynthesis. **a** Determination of PM100117/18 UPLC *peak areas* in strains *S. caniferus* GUA-06-05-006A (*white bars*) and Δ*gonG4* (*grey bars*). **b** *Peak area* quantification of PM100117/18 in strains GUA-pSETH (*white bars*) and OE*gonG4* (*grey bars*). In all cases, data are means ± standard deviations from three independent experiments

We conducted a series of experiments to investigate the possible functional role of the putative GT GonG4. Firstly, a precise quantification of PM100117/18 production in strain Δ*gonG4* and wild type *S. caniferus* GUA-06-05-006A confirmed that *gonG4* deletion has no impact on the production level of these compounds, suggesting that putative GT GonG4 might not be involved in their biosynthesis (Fig. 5a). To assess the potential of GonG4 to transfer 2,6DOHs to PM100117/18, we increased its presence in *S. caniferus* GUA-06-05-006A by over-expressing *gonG4* using the integrative plasmid pS*GonG4*. Precise UPLC-based quantification of PM100117/18 production in the resultant strain, OE*gonG4*, did not show any change with respect to the wild type strain (Fig. 5b; Additional file 3: Fig. S3a). Next, we reasoned that, under certain conditions, transfer of 2-deoxy-l-fucose and l-rhodinose as second 2,6DOH units to form PM100117 and PM100118, respectively, might involve two different GTs. However no potential derivative containing the second sugar moiety was detected when *gonG4* was over-expressed in Δ*gonG2* (Additional file 3: Fig. S3b). On the other hand, a defensive function consisting of transferring of glucose units to antibiotic agents, which as a result become inactivated, has been proposed for several GTs [33–35]. Notwithstanding, the involvement of GonG4 in PM100117/18 inactivation by glycosylation has not been proven by experiments consisting of *gonG4* over-expression and subsequent feeding with PM100118. Therefore, the role of gonG4 on PM100117/18, if any, remains elusive.

### In vitro cytotoxicity analysis of PM100117 and PM100118 derivatives

We assessed the in vitro cytotoxic activity of the novel analogues generated against cancer cell lines A549 (human lung carcinoma cells), PSN1 (pancreas carcinoma), MDA-MB-231 (human breast adenocarcinoma) and HT29 (human colorectal carcinoma). The GI_50_ (50% inhibition on cell growth) and TGI (total growth inhibition) concentrations of compounds 3 and 4 increased slightly with respect to PM100117/18, indicating a drop in the antitumor activity of the derivatives relative to the parental products (Table 1). The remaining novel products, 5-13, showed a more remarkable antitumor activity reduction, showing GI_50_ concentrations up to ten times higher than the natural compounds.

**Table 1:** In vitro cytotoxic activity of compounds 1–13

Compound		A549 (μM)	HT29 (μM)	MDA-MB-231 (μM)	PSN1 (μM)
**1** PM100117	GI_50_	1.52	3.04	2.66	nd
	TGI	1.84	3.23	2.79	nd
	LC_50_	2.22	3.61	2.97	nd
**2** PM100118	GI_50_	2.24	1.92	1.73	nd
	TGI	3.13	2.81	2.75	nd
	LC_50_	4.28	4.09	4.16	nd
**3** From mutant Δ*gonCM*	GI_50_	1.94	5.03	4.06	2.97
	TGI	2.39	6.07	6.39	3.23
	LC_50_	3.16	>6.45	>6.45	>6.45
**4** From mutant Δ*gonCM*	GI_50_	2.02	5.22	4.39	3.13
	TGI	2.58	6.76	5.78	3.41
	LC_50_	>6.97	>6.97	>6.76	>6.97
**5** From mutant Δ*gonD3*	GI_50_	>6.82	>6.82	>6.82	>6.82
	TGI	nd	nd	nd	nd
	LC_50_	nd	nd	nd	nd
**6**,** 7**,** 8 **and** 9** From mutant Δ*gonE* ^a^	GI_50_	>10.70	>10.70	>10.70	>10.70
	TGI	nd	nd	nd	nd
	LC_50_	nd	nd	nd	nd
**10** From mutant Δ*gonG1*	GI_50_	>10.70	>10.70	>10.70	>10.70
	TGI	nd	nd	nd	nd
	LC_50_	nd	nd	nd	nd
**11** and** 12** From mutant Δ*gonG2* ^a^	GI_50_	>9.20	>9.20	>9.20	>9.20
	TGI	nd	nd	nd	nd
	LC_50_	nd	nd	nd	nd
**13** From mutant Δ*gonG3*	GI_50_	>6.90	>6.90	>6.90	>6.90
	TGI	nd	nd	nd	nd
	LC_50_	nd	nd	nd	nd

^a^These compounds were assayed individually

*GI*_50_ compound concentration that produces 50% inhibition on cell growth as compared to control cells, *TGI* compound concentration that produces total growth inhibition as compared to control cells, *LC*_50_ compound concentration that produces 50% cell death as compared to control cells, *nd* values not determined

## Discussion

Up to thirteen ORFs from the PM100117 and PM100118 biosynthesis gene cluster can reliably be predicted to code for enzymes potentially involved in glycosylation reactions. Although bioinformatics analysis of the entire PM100117/18 gene cluster was previously addressed [32], here we provide additional in silico data supporting function assignment to putative DOH biosynthesis and GT enzymes (Additional file 1). The biosynthesis pathways of the PM100117/18 2,6DOHs moieties (Fig. 2) that can be deduced from these functions are congruent with 2,6DOHs biosynthesis reaction schemes previously reported [36]. Only a gene coding for an EryBII-type 3-ketoreductase (3KR, Fig. 2) presumably involved in the formation of a NDP-4-keto-2,6DOH intermediate with an axial hydroxyl group at C3 has not been located within the gene cluster. The absence of functions required for 2,6DOHs biosynthesis has been also described in the sequence analysis of the gene cluster of other glycosylated NPs, such as spinosyn [37], brasilicardin A [38] or mithramycin [39]. However, in the later example, gene *mtmC* has been shown to code for a ketoreductase/methyltrasnferase bifunctional enzyme [40, 41]. Thus, 3-*C*-methyltransferase GonCM might be also responsible for the 3-ketoreduction reaction leading to l-axenose. Alternatively, methylation of the NDP-4-keto-2,6-dideoxyl-d-Glucose intermediate by GonCM could be sequential to GonR3 activity (Fig. 2). Furthermore, the involvement of 3-*C*-methyltransferase GonCM and 3-dehydratase GonD3 in reaction steps of l-axenose and l-rhodinose biosynthesis, respectively, is also supported by genetic engineering experiments (Fig. 3). Likewise, implication of 3,5-epimerase GonE in PM100117/18 biosynthesis has been also confirmed. Nevertheless, putative isomerase activity of this enzyme has not been further verified because of the impossibility to conduct NMR analysis of compounds 8 and 9. It is noteworthy the presence in the PM100117/18 gene cluster of two genes, *gonR1* and *gonR2*, coding for putative 4-ketoreductase enzymes. However, the involvement of these enzymes in the biosynthesis of the three PM100117/18 DOH moieties has not been fully demonstrated by genetic experiments. The inactivation of *gonR1* has no apparent effect on PM100117/18 biosynthesis, and its lack could be complemented by the presence of an additional 4-ketoreductase encoded in *S. caniferus* chromosome, being *gonR2* a good candidate. On the other hand, the impossibility of producing a *gonR2* deletion mutant suggests the involvement of this gene in important primary processes such as, for example, supply of essential sugars for cell-wall formation. A similar connection of primary and secondary metabolism via DOH biosynthesis genes has been previously reported for the biosynthesis of l-rhamnose required for spinosyn A production in *Saccharopolyspora spinosa* [42].

Gene deletion experiments have also enabled the identification of GTs responsible for transfer of each 2,6DOH sugar moiety to its corresponding position of the PM100117/18 glycosylation pattern. Putative GT GonG1 is involved in transfer of the first sugar unit, l-axenose, since upon deletion of *gonG1* no glycosylated PM100117/18 derivatives were produced (Fig. 4). Following l-axenose transfer, GonG2 is responsible for the introduction of the second sugar moiety, 2-deoxy-l-fucose and l-rhodinose, to PM100117 and PM100118 biosynthesis, respectively. Since two different 2,6DOH moieties can be transferred as second sugar unit, we previously predicted the involvement in this step of two GTs, each dedicated to transfer only one 2,6DOH. Nevertheless, UPLC and MS analysis did not detect production in Δ*gonG2* strain of PM100117/18 analogues carrying the second DOH unit (Fig. 4), thus pointing at GonG2 as the only GT responsible for the introduction of both 2,6DOHs at second position of the PM100117/18 glycosylation pattern. Finally, the structure of analogues derived from Δ*gonG3* (Fig. 4c), which lack the third sugar moiety, suggests that GonG3 is the GT in charge of transferring l-rhodinose to the naphthoquinone moiety of the PM100117/18 glycosylation profile. Surprisingly, the functional role of the putative GT GonG4 remains elusive. As a primary hypothesis, we speculated a role for GonG4 on transferring 2-deoxy-l-fucose or l-rhodinose as second sugar moiety. However, neither PM100117 nor PM100118 production was detected upon *gonG4* over-expression in Δ*gonG2* mutant strain (Fig. 5d). Alternatively, GonG4 might be inactive, but sequence alignment with functional glycosyltransferases did not reveal obvious differences supporting that possibility, or require post-translational regulatory signals for its activation. Moreover, it seems plausible also to contemplate that under certain environmental conditions, GonG4 might have a role on transferring 2,6DOH moieties other than l-axenose, 2-deoxy-l-fucose or l-rhodinose, leading to the production of natural PM100117/18 derivatives. This is consistent with the presence in the PM100117/18 gene cluster of two ORFs, *gonR1* and *gonR2*, coding for putative 4-ketoreductase enzymes. Their presence in the cluster allows us to speculate with the biosynthesis of additional 2,6DOHs. For instance, last step of 2-deoxy-l-fucose an l-rhodinose biosynthesis could be branched towards the formation of l-olivose and l-amicetose, respectively, if either GonR1 or GonR2 were able to produce hydroxyl groups in equatorial configuration at C4. However, based on sequence analysis data, it is not possible to forecast the resulting stereo configuration after GonR1 and/or GonR2 activity. Otherwise, we cannot rule out the biosynthesis of other 2,6DOHs by the activity of enzymes encoded in other locations of *S. caniferus* chromosome.

Standing by the chemical structure of PM100117/18 analogues produced by the mutant strains analyzed in this work, we can make interesting conclusions on the substrate flexibility of GonG1, GonG2 and GonG3. When genes coding for these putative GTs are individually deleted, none of the two remaining GTs is flexible to catalyze the transfer of its own natural sugar to the acceptor aglycone. This apparently low promiscuity of the three GTs towards the acceptor substrate contrasts with the flexibility showed by GonG1 to transfer l-rhodinose (compounds 3 and 4) as first sugar moiety to the PM100117/18 polyketide ring (Fig. 3) in the Δ*gonCM* mutant, in which l-axenose biosynthesis is presumably suppressed. Another interesting observation from the structure of the compounds detected in mutants Δ*gonCM* and Δ*gonG2* is the production of a derivative with a hydroxyl functional group at C18 of the PM100117/18 macrolactone ring (compounds 3 and 11), a feature also observed in the polyketide skeleton of the structurally related antibiotic brasilinolide A [43]. This seems to reinforce the structural and biosynthetic relationships between both compounds. On the other hand, accumulation of the naphthoquinone moiety (compound 6) is exclusively detected in strain Δ*gonE*, and not in other mutant strains, such as Δ*gonG1* or Δ*gonG2*, in which this chromophore is neither incorporated into the PM100117/18 structure (Figs. 3, 4). Based on our current knowledge on PM100117/18 biosynthesis, no suitable explanation to this observation can be exposed. Moreover, the naphthoquinone chromophore and its derivative compound 7 do not show in vitro cytotoxic activity (Table 1), in spite of the structural and biosynthetic connection of these products to menaquinone, whose antitumor properties have been repeatedly noted [44, 45]. Furthermore, as it was predictable from a previous report [32], removal of large portions from PM100117/18 structure, as displayed in different degrees by compounds 5, 10, 11, 12 and 13, resulted in a remarkable decrease of in vitro cytotoxicity. Only compounds 3 and 4, which preserve chemical structures containing three 2,6DOHs, retain activity levels comparable to those showed by the parental products (Table 1).

Given their biological activity, PM100117/18 constitute appealing potential targets of biochemical, genetic and medical research. Findings on PM100117/18 biosynthesis reported in this work will enable the manipulation of the described glycosylation machinery by genetic methods in order to generate additional structural diversity of these compounds.

## Conclusions

PM100117 and PM100118 exhibit a composition of structural elements with biosynthetic interest, such as a naphthoquinone chromophore and three 2,6DOHs. Previously, we showed the vital role of the naphthoquinone unit on the PM100117/18 biological activity. Herein, the 2,6DOHs moieties are proven to be equally essential to PM100117/18 cytotoxicity. Therefore, manipulations aimed to biosynthesize novel PM100117/18 antitumor derivatives must contemplate the preservation of these structural components. In addition, we have demonstrated that PM100117/18 glycosylation can be genetically manipulated to direct the biosynthesis of novel derivatives. At least one GT coded within the PM100117/18 gene cluster has shown certain flexibility towards its natural 2,6DOH donor substrate, widening future perspectives to successfully fulfill the engineering of novel analogues by combinatorial biosynthesis methods. On the other hand, the uncertain functional role of the putative GT GonG4, and the presence in the PM100117/18 gene cluster of two ORFs coding for putative 4-ketoreductase enzymes, invites to speculate with the biosynthesis, under certain environmental conditions, of additional natural products structurally related to PM100117/18. The role of these enzymes deserves further investigations, which might require performance of biochemical and enzymatic analyses.

## Methods

### Bacterial strains, media and culture conditions

The PM100117/18 producer *S. caniferus* GUA-06-05-006A [31] and its isogenic strains were routinely cultivated on solid medium A [46] or in TSB [47] medium. Cultures for PM100117/18 production experiments were carried out in supplemented MS medium (SMS) [32] at 30 °C and rotary shaking at 200 rpm for 7 days. A more detailed description of these fermentations can be found in a previous report [32]. The *Escherichia coli* strains used for gene cloning (DH10B) [48] and intergeneric conjugation (ET12567/pUB307) [47] were grown at 37 °C in 2xTY medium. All media were supplemented, when required, with the appropriate antibiotic for plasmid selection following standard directions [47].

### Analysis of PM100117 and PM100118 production

Whole-culture samples (2 ml) from S*. caniferus* GUA-06-05-006A wild type, or the corresponding mutant strains, were mixed with the same volume of ethyl acetate and shacked vigorously at room temperature for 1 h. The organic phase was collected and evaporated to dryness with a Speed-Vac. The residue was re-dissolved in 60 µl of methanol:DMSO (1:1), clarified by centrifugation and then subjected to UPLC and LC/MS analysis as described elsewhere [49]. To assess the PM100117/18 production level, SMS medium was *vacuum*-filtrated with nitrocellulose filters of 0.45 µm pore size (Millipore). After 7-days cultivation of the respective strains in clarified SMS medium, 2-ml whole-culture samples were collected by triplicate and centrifuged. The cell pellet was washed twice with milliQ water and then incubated for 72 h at 100 °C to determine cell dry-weight of the culture. Metabolite extraction and UPLC analysis were performed as mentioned above. The final sample solution was appropriately diluted to ensure reliable linear-range PM100117/18 quantifications. Peak areas were determined by triplicate and normalized with respect to the corresponding dry-weight values.

### Purification of PM100117 and PM100118 derivatives

For purification of compounds 3–13, cultures of the corresponding producing strains were centrifuged and the supernatants were filtered and applied to a solid-phase extraction cartridge (Sep-Pak Vac C18, 10 g, Waters). The retained material in each case was eluted with a mixture of methanol and 0.05% TFA in water. A linear gradient from 0 to 100% methanol in 60 min, at 10 ml/min, was used. Fractions were taken every 5 min and analyzed by UPLC. The fractions containing the desired compounds were evaporated *in vacuo* and subsequently re-dissolved in a small volume of a mixture of DMSO and methanol (50:50). For purification of compounds 6 and 7 the extracted broth was acidified by adding formic acid up to 1% and re-extracted with one volume of ethyl acetate. Additionally, purification of compounds 3 and 4, required extraction from cell pellets with ethyl acetate. The resulting pellet extracts were similarly evaporated and re-dissolved. The compounds of interest were purified by preparative HPLC using a SunFire C18 column (10 µm, 10 × 250 mm, Waters). Compounds were chromatographed with mixtures of acetonitrile or methanol and 0.05% TFA in water in isocratic conditions optimized for each peak, at 7 ml/min, and when needed (compounds 3, 4, 6 and 7) collected on 0.1 M phosphate buffer, pH 7.0. After every purification step, the collected compounds were diluted fourfold with water and then applied to a solid-phase extraction cartridge (Sep-Pak C18, Waters). The cartridge was washed with water, the retained compound was eluted with methanol and dried in vacuo. Once the purification was finished, the compounds were dissolved in a mixture of tert-butanol and water (1:1) and lyophilized.

### In vitro cytotoxicity assay

The procedure to determine the in vitro antitumor activity of the PM100117/18 analogues obtained in this work was previously described [31, 32].

### DNA manipulation and plasmids construction

DNA manipulation experiments in *Escherichia coli* and S*. caniferus* GUA-06-05-006A were carried out according to standard protocols [47, 50]. PCR amplifications were performed by using Herculase II Fusion polymerase (Agilent Technologies) with the touchdown PCR procedure previously reported [32]. Primers and plasmids used in this work are described in (Additional file 4: Table S6). To accomplish single deletion of genes *gonCM*, *gonD3*, *gonE*, *gonR1*, *gonR2*, *gonG1*, *gonG2*, *gonG3* and *gonG4*, the downstream DNA sequence of the corresponding target ORFs were amplified with the primer pairs** EcoRV**-*CM*/**NdeI**-*CM*,** EcoRV**-*D3*/**BamHI**-*D3*,** EcoRV**-*E*/**BamHI**-*E*,** BamHI**-*R1*/**EcoRV**-*R1*,** EcoRV**-*R2*/**BamHI**-*R2*,** BamHI**-*G1*/**EcoRV**-*G1*,** BglII**-*G2*/**EcoRV**-*G2*,** NdeI**-*G3*/**EcoRV**-*G3* and** BamHI**-*G4*/**EcoRV**-*G4*, respectively, and cloned in the designated restriction sites (bold) of plasmid pEFBA-oriT [51], downstream to the *aac*(*3*)*IV* gene, which confers resistance to apramycin (Apm^R^). Then, the upstream sequence of the cited target genes were likewise amplified with the primer pairs** NsiI**-*CM*/**SpeI**-*CM*,** NsiI**-*D3*/**SpeI**-*D3*,** NsiI**-*E*/**SpeI**-*E*,** SpeI**-*R1*/**NsiI**-*R1*,** NsiI**-*R2*/**SpeI**-*R2*,** SpeI**-*G1*/**NsiI**-*G1,* **SpeI**-*G2*/**NsiI**-*G2*,** XbaI**-*G3*/**NsiI**-*G3* and** SpeI**-*G4*/**NsiI**-*G4*, respectively, and cloned upstream to the *aac*(*3*)*IV* gene. Finally, the hygromycin B resistance (Hyg^R^) gene marker, *hyg*, was extracted by XbaI/NheI digestion from plasmid pLHyg [52] and introduced in the XbaI site of the deletion plasmids. Gene *hyg* allows recognizing clones in which a complete gene replacement by a double cross-over has taken place (Hyg^S^ Apm^R^) from those in which a single cross-over event has integrated the deletion plasmid into the chromosome (Hyg^R^ Apm^R^). To construct the pSETH plasmid backbone, used to achieve gene complementation and over-expression experiments, a 1.6-kb DNA fragment was amplified from plasmid pDR4 [53] with the primer pair NcoI-dHG^R^/NcoI-rvHG^R^ and cloned in the NcoI site of plasmid pSETec [49], which harbors the constitutive ermE*p promoter. Plasmids intended to accomplish gene complementation and over-expression were constructed by inserting the respective target ORF in the BamHI/EcoRV sites of pSETH (Additional file 4: Table S6). Correct fragment insertion in deletion and complementation plasmids was verified by PCR and sequencing.

### Gene mutation and complementation by intergeneric conjugation

Deletion plasmids pD*gonCM*, pD*gonD3*, pD*gonE*, pD*gonR1*, pD*gonG1*, pD*gonG2*, pD*gonG3* and pD*gonG4* (Table S6) were introduced in S*. caniferus* GUA-06-05-006A by intergeneric conjugation [32] to generate deletion strains Δ*gonCM*, Δ*gonD3*, Δ*gonE*, Δ*gonR1*, Δ*gonG1*, Δ*gonG2*, Δ*gonG3* and Δ*gonG4*, respectively. ORF replacement in the resulting mutant strains was confirmed by PCR (Additional file 4: Fig. S5) with the primer pairs indicated in Table S6 (Additional file 4: Table S6). Complementation plasmids pC*gonCM*, pC*gonD3*, pC*gonE*, pC*gonG1*, pC*gonG2* and pC*gonG3* were then introduced by the same procedure in the corresponding mutant strains to afford strains C*gonCM*, C*gonD3*, C*gonE*, C*gonG1*, C*gonG2*, C*gonG3*, in which PM100117/18 biosynthesis was partially restored (Additional file 5: Fig S6). Plasmids, pSETH, pC*gonR2* and pC*gonG4* were also transferred to S*. caniferus* GUA-06-05-006A to produce strains GUA-pSETH, OE*gonR2* and OE*gonG4*, respectively. On the other hand, transfer of pSETH and pC*gonG4* to mutant Δ*gonG2* yielded strains Δ*G2*-pSETH and Δ*G2*-OE*G4*, respectively. The exconjugant selection procedure followed in this work was thoroughly described in a previous report [31].

## Additional files

**Additional file 1.** *In silico* analysis of sugar biosynthesis and glycosyltransferase genes.

**Additional file 2.** **Fig. S1.** Mass and NMR spectra compounds 3-13. **Tables S1–S5.** NMR data of compounds 3-13.

**Additional file 3.** **Fig. S2.** Effect of *gonR2* over-expression on PM100117/18 biosynthesis. **Fig. S3.** Effects of *gonG4* over-expression on *S. caniferus* GUA-06-05-006A and Δ*gonG2*. Methods: Feeding experiment. **Fig. S4.** Heterologous expression of *gonG4* in *Streptomyces albus* J1074 and feeding with PM100118.

**Additional file 4.** **Table S6.** Primers and plasmids used in this work. **Fig. S5.** Gene deletion confirmation by PCR in mutant strains ∆*gonG1*, ∆*gonG2*, ∆*gonG3*, ∆*gonG4*, ∆*gonR1*, ∆*gonE*, ∆*gonCM* and ∆*gonD3*. 

**Additional file 5.** **Fig. S6.** Genetic complementation of mutant strains Δ*gonCM*, Δ*gonD3*, Δ*gonE*, Δ*gonG1*, Δ*gonG2* and Δ*gonG3*.

## Abbreviations

Apm^R^: apramycin resistant phenotype
DMSO: dimethyl sulfoxide
DOH: deoxysugar
2,6DOH: 2,6-dideoxyhexoses
6DOH: 6-deoxyhexoses
GT: glycosyltransferase
HPLC: high-performance liquid chromatography
Hyg^R^: hygromycin B resistant phenotype
Hyg^s^: hygromycin B sensitive phenotype
LC: liquid chromatography
MS: mass spectrometry
NDP: nucleoside diphosphate
NMP: nucleoside monophosphate
HPLC: high-performance liquid chromatography
NMR: nuclear magnetic resonance
NP: natural product
ORF: open reading frame
Rt: retention time
TFA: trifluoroacetic acid
UPLC: ultra-performance liquid chromatography
