# Supplementary material for: Elucidation of the glycosylation steps during biosynthesis of antitumor macrolides PM100117 and PM100118 and engineering for novel derivatives
Source: Microb Cell Fact. 2016 Nov 9;15:187. doi: 10.1186/s12934-016-0591-7 (PMC5103430; doi:10.1186/s12934-016-0591-7)
Supplement: Supplementary file 1 — Additional file 1. In silico analysis of sugar biosynthesis and glycosyltransferase genes. [file 12934_2016_591_MOESM1_ESM.pdf]

In silico analysis of sugar biosynthesis and glycosyltransferase genes

Putative function assignment to proteins presumably involved in 2,6-dideoxysugar (2,6DOH) biosynthesis, and the identification of putative glycosyltransferase (GT) genes, has been performed by BLAST-based database scan [1] at the National Center for Biotechnology Information (NCBI) and sequence alignment by EMBOSS water [2] from the European Molecular Biology Laboratory (EMBL).

Bioinformatics analysis of GonGS detected an rmlA_long (TIGR01208) and a G1P_TT_long (cd04189) domain, very conserved among glucose-1-phosphate thymidylyltransferase enzymes, suggesting the involvement of this protein in hexose activation by nucleotide diphosphate (NDP) transfer. GonD2 presents and outstanding identity (I: 76%) and similarity (S: 83%) to the biochemically characterized dTDP-glucose 4,6-dehydratase SsfS2 from the antitumor tetracycline SF2575 gene cluster of *Streptomyces* sp. SF2575 [3], and displays a dTDP_GD_SDR_e (cd05246) domain, a distinctive feature of the extended short chain class of dehydrogenase/reductase (SDR) enzymes. GonD1 shows high resemblance [I: 50%, S: 66%] to the putative dTDP-4-keto-6DOH 2,3-dehydratase Sim20 from the simocyclinone biosynthetic gene cluster of *Streptomyces antibioticus* Tu 6040 [4]. Besides, GonD1 contains the conserved hexose dehydratase domain pfam03559, which hallmarks the family of NDP-hexose 2,3-dehydratase enzymes frequently involved in bioactive NPs biosynthesis. GonR3 presents high resemblance with many putative NDP-hexose 3-ketoreductase proteins, including PokS4 [I: 52%, S: 61%] from the polyketomycin gene cluster in *Streptomyces diastatochromogenes* [5], ChlC4 [I: 49%, S: 63%] from the chlorothricin gene cluster in *Streptomyces antibioticus* [6] or PlaA5 [I: 48%, S: 61%] from the phenalinolactone gene cluster in *Streptomyces* sp. Tu6071 [7]. These three putative NDP-hexose 3-ketoreductases are involved in the biosynthesis of L-axenose, olivose and L-amicetose,

respectively, and presumably render a hydroxyl group at C-3 with an equatorial configuration. Comparison of GonR3 with the NCBI domain database predicts formation of a NAD-binding Rossmann fold (GxGxxG) and detects *N*-terminal (pfam01408) and *C*-terminal (pfam02894) oxidoreductase domains. GonCM features various NCBI database methyltransferase domains, including a zinc binding motif (pfam08421) with four conserved cysteine residues at the protein *N*-terminus. In addition, sequence search by BLAST revealed high identity of GonCM to TxnM1 [I: 79%, S: 87%] from the trioxacarcin gene cluster in *Streptomyces bottropensis* [8] and PokS8 [I: 72%, S: 81%] from the polyketomycin gene cluster in *Streptomyces diastatochromogenes* [5]. Protein GonE possesses a significant resemblance to dTDP-4-deoxyglucose 3,5-epimerase enzymes TxnB6 and PokS7, also implicated in the biosynthesis of trioxacarcin and polyketomycin, respectively. The assignment of GonE with 3,5-isomerase activity is reinforce by the identification in this protein of dTDP_sugar_isom (pfam00908), RfbC (COG1898) and RmlC (TIGR1221) motifs, from the NCBI`s conserved domains database, which are distinctive of the dTDP-4-dehydrorhamnose 3,5-epimerase family of isomerase enzymes [9]. BLAST scan of GonD3 produced two homology hits, aminotransferase and NDP-hexose 3,4-dehydratase. Nonetheless, in addition to genetic engineering data supporting the latest functional role, considerable identity and similarity is also found between GonD3 and diverse putative NDP-hexose 3,4-dehydratase enzymes such as NanG3 [I: 72%, S: 81%], which is presumably involved in the biosynthesis of the nanchangmycin L-rhodinose moiety in *Streptomyces nanchangensis* [10]. Despite this, no dehydratase/reductase specific domains were detected along GonD3 sequence. BLAST analysis of GonR1 and GonR2, which display 40% identity and 55% similarity among them, finds multiple sequence homologies with putative 4-

dehydratase/epimerase/reductase enzymes, such as PokS6 and TxnB5 from the already mentioned polyketomycin and trioxacarcin gene clusters of *S. diastatochromogenes* [5] and *S. bottropensis* [8], respectively. Function assignment to GonR1 and GonR2 as 4-ketoreductase is also endorsed by the detection of relevant NCBI's domains, namely dTDP_HR_like_SDR_e (cd05254), Epimerase_Csub (pfam13950), WcaG (COG0451), rmlD (TIGR1214) and PRK09987 (PRK09987).

Sequence analysis of GonG1-GonG4 detected a cl100113 domain distinctive of the GT-B superfamily of GTs in addition to several other characteristic GT domains both at the protein *C*- and *N*-terminus. Beside, GonG1shows high identity and similarity to the L-2-deoxyfucosyltransferase AknK (I: 43%, S: 59%), involved in the biosynthesis of aclacinomycins [11] in *Streptomyces galilaeus*, and presents a high resemblance to other putative GTs such as NivK (I: 43%, S: 59%) from the nivetetracyclates A and B gene cluster in *Streptomyces niveus* [12]. The putative GT GonG2 is 42% identical and 54% similar to the GT CalG2 of *Micromonospora echinospora*, which has been implicated by *in vitro* biochemical analyses in calicheamicin glycosylation. GonG3 also possesses high identity (39%) and similarity (53%) to the same protein, CalG2, and a high resemblance (I:39%, S: 52%) to the GT LipGtf from the α-Lipomycin gene cluster in *Streptomyces aureofaciens* Tü117 [13]. Conversely, in spite of containing various distinctive GT domains, comparison of GonG4 with other proteins in database does not reveal a clear resemblance to any known GT.

References

1. Altschul SF, Madden TL, Schäffer AA, Zhang J, Zhang Z, Miller W, Lipman DJ: **Gapped BLAST and PSI-BLAST: A new generation of protein database search programs**. *Nucleic Acids Research* 1997, **25**:3389–3402.

2. Li W, Cowley A, Uludag M, Gur T, McWilliam H, Squizzato S, Park YM, Buso N, Lopez R: **The EMBL-EBI bioinformatics web and programmatic tools framework.** *Nucleic Acids Res* 2015, **43**(Web Server issue):W580–W580–4.

3. Pickens LB, Kim W, Wang P, Zhou H, Watanabe K, Gomi S, Tang Y: **Biochemical analysis of the biosynthetic pathway of an anticancer tetracycline SF2575**. *J Am Chem Soc* 2009, **131**:17677–17689.

4. Galm U, Schimana J, Fiedler HP, Schmidt J, Li SM, Heide L: **Cloning and analysis of the simocyclinone biosynthetic gene cluster of *Streptomyces antibioticus* Tü 6040**. *Arch Microbiol* 2002, **178**:102–114.

5. Daum M, Peintner I, Linnenbrink A, Frerich A, Weber M, Paululat T, Bechthold A: **Organisation of the biosynthetic gene cluster and tailoring enzymes in the biosynthesis of the tetracyclic quinone glycoside antibiotic polyketomycin**. *ChemBioChem* 2009, **10**:1073–1083.

6. Jia X-Y, Tian Z-H, Shao L, Qu X-D, Zhao Q-F, Tang J, Tang G-L, Liu W: **Genetic characterization of the chlorothricin gene cluster as a model for spirotetronate antibiotic biosynthesis.** *Chem Biol* 2006, **13**:575–585.

7. Dürr C, Schnell HJ, Luzhetskyy A, Murillo R, Weber M, Welzel K, Vente A, Bechthold A: **Biosynthesis of the terpene phenalinolactone in *Streptomyces* sp. Tü6071: Analysis of the gene cluster and generation of derivatives**. *Chem Biol* 2006, **13**:365–377.

8. Yang K, Qi L-H, Zhang M, Hou X-F, Pan H-X, Tang G-L, Wang W, Yuan H: **The SARP family regulator Txn9 and two-component response regulator Txn11 are key activators for trioxacarcin biosynthesis in *Streptomyces bottropensis***. *Curr Microbiol* 2015, **71**:458–464.

9. Oka T, Nemoto T, Jigami Y: **Functional analysis of *Arabidopsis thaliana* RHM2/MUM4, a multidomain protein involved in UDP-D-glucose to UDP-L-rhamnose conversion**. *J Biol Chem* 2007, **282**:5389–5403.

10. Sun Y, Zhou X, Dong H, Tu G, Wang M, Wang B, Deng Z: **A complete gene cluster from *Streptomyces nanchangensis* NS3226 encoding biosynthesis of the polyether ionophore nanchangmycin**. *Chem Biol* 2003, **10**:431–441.

11. Lu W, Leimkuhler C, Oberthür M, Kahne D, Walsh CT: **AknK Is an L-2-deoxyfucosyltransferase in the biosynthesis of the anthracycline aclacinomycin A**. *Biochemistry* 2004, **43**:4548–4558.

12. Chen C, Liu X, Abdel-Mageed WM, Guo H, Hou W, Jaspars M, Li L, Xie F, Ren B,

Wang Q, Dai H, Song F, Zhang L: **Nivetetracyclates A and B: Novel compounds isolated from *streptomyces niveus***. *Org Lett* 2013, **15**:5762–5765.

13. Bihlmaier C, Welle E, Hofmann C, Welzel K, Vente a, Breitling E, Müller M, Glaser S, Bechthold a: **Biosynthetic gene cluster for the polyenoyltetramic acid alpha-lipomycin.** *Antimicrob Agents Chemother* 2006, **50**:2113–2121.
