# Supplementary material for: Elucidation of the glycosylation steps during biosynthesis of antitumor macrolides PM100117 and PM100118 and engineering for novel derivatives
Source: Microb Cell Fact. 2016 Nov 9;15:187. doi: 10.1186/s12934-016-0591-7 (PMC5103430; doi:10.1186/s12934-016-0591-7)
Supplement: Supplementary file 2 — Additional file 2. Fig. S1. Mass and NMR spectra compounds 3-13. Tables S1–S5. NMR data of compounds 3-13. [file 12934_2016_591_MOESM2_ESM.pdf]

Fig. S1. Mass and NMR spectra of compounds **3-13**. ^{1}H (CD_3OD, 500 MHz for ^{1}H and 125 MHz for ^{13}C) and MS spectra of compounds **3-13**. TOCSY and ROESY spectra of compounds **3**, **5** and **10-13**. ^{13}C (CD_3OD, 500 MHz for ^{1}H and 125 MHz for ^{13}C), gCOSY, gHSQC and gHMBC spectra of compounds **3** and **10-13**.

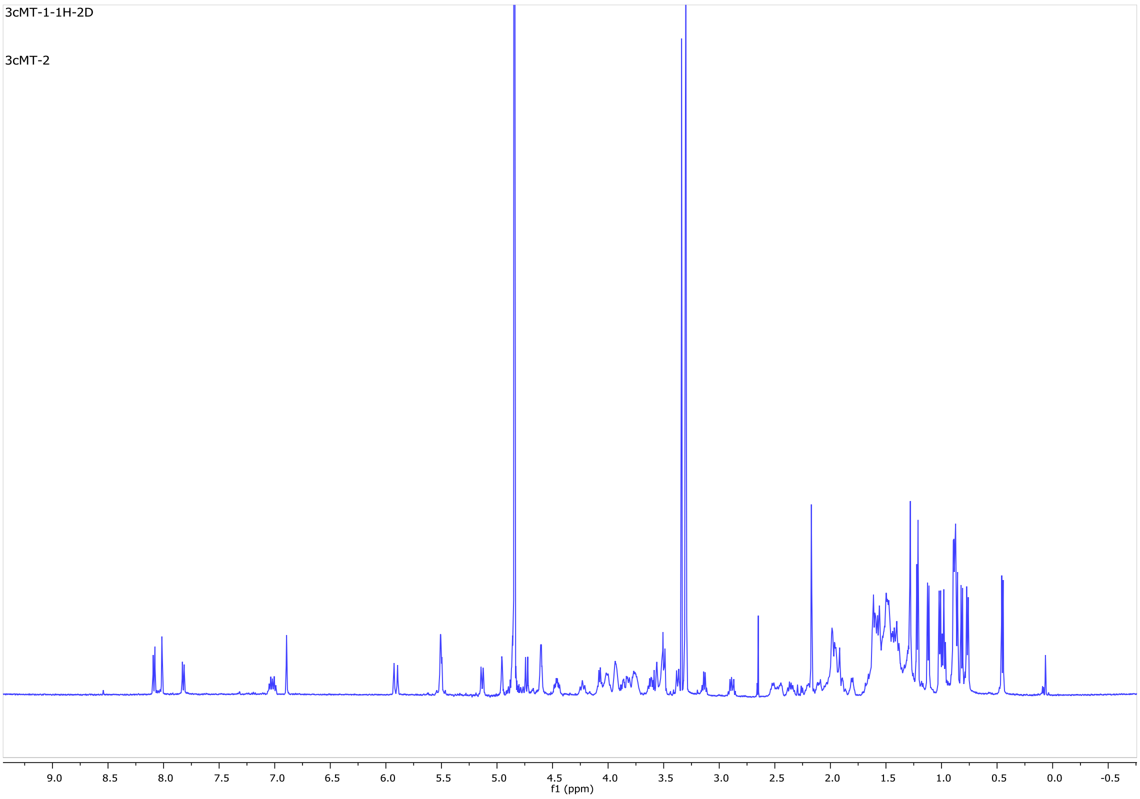

^{1}H spectrum of **3** (CD_3OD, 500 MHz for ^{1}H and 125 MHz for ^{13}C)

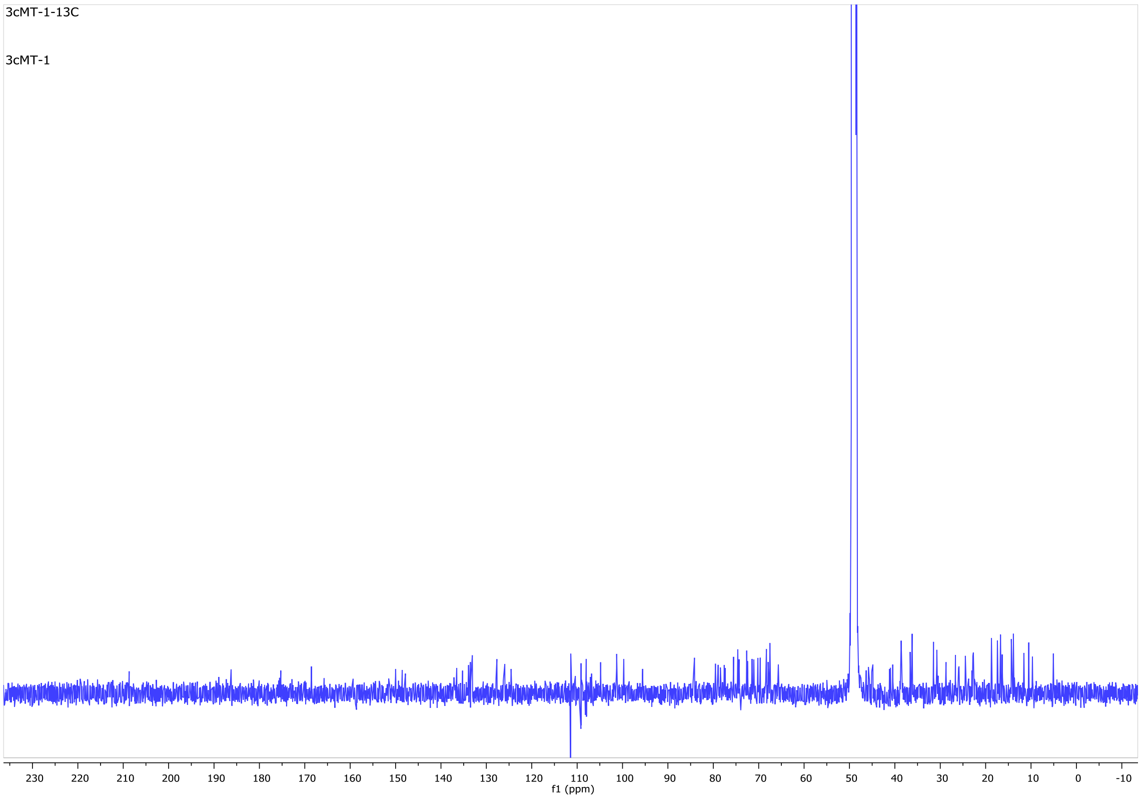

^{13}C spectrum of **3** (CD_3OD, 500 MHz for ^1H and 125 MHz for ^{13}C)

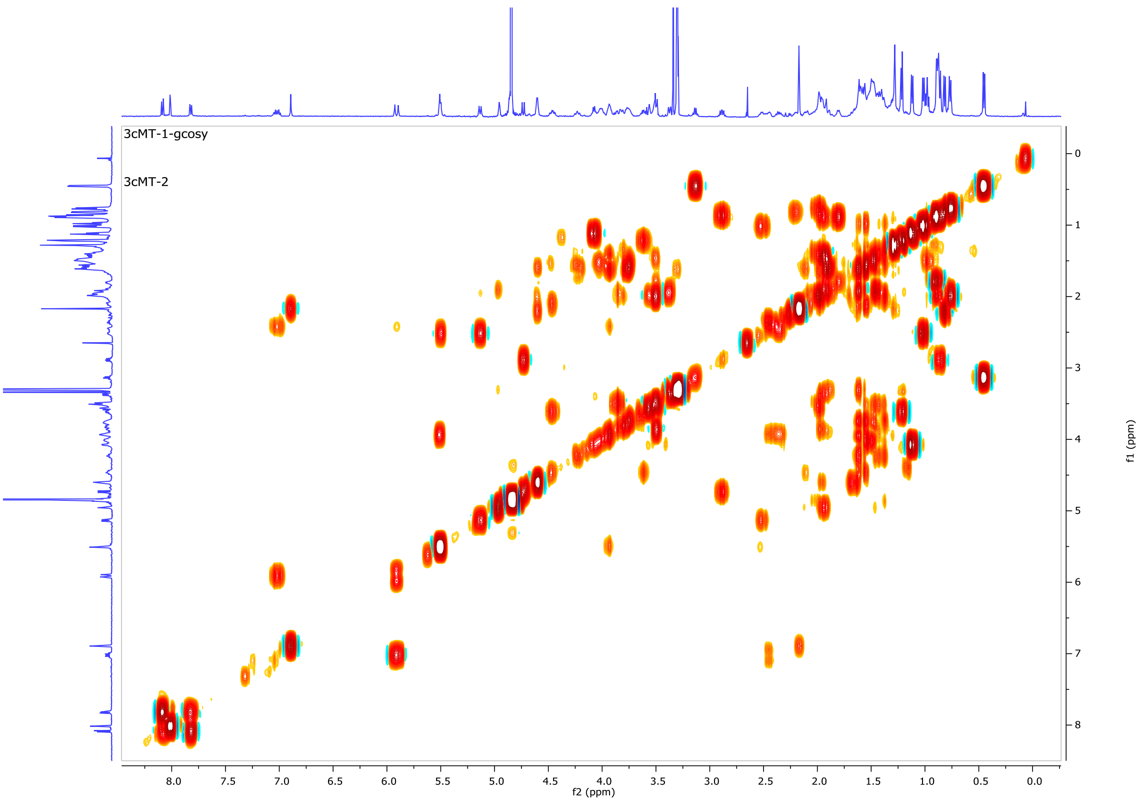

gCOSY spectrum of **3** (CD_3OD, 500 MHz for ^1H and 125 MHz for ^{13}C)

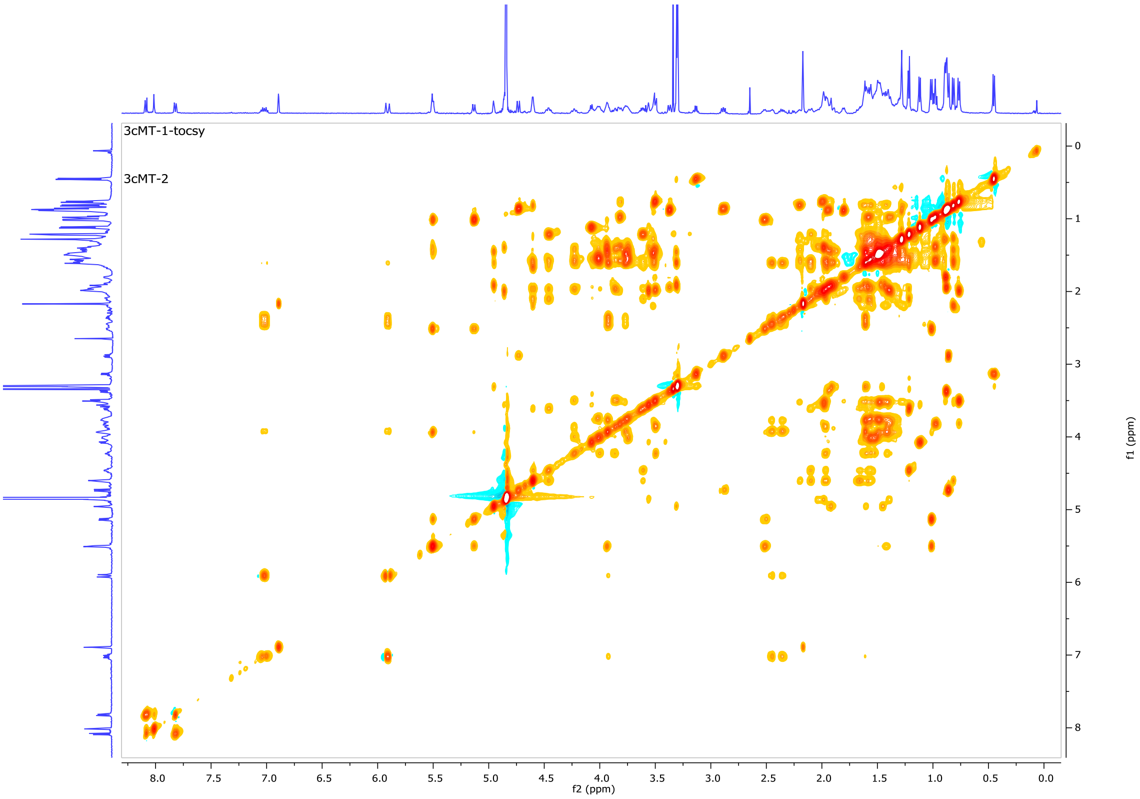

TOCSY spectrum of **3** (CD_3OD, 500 MHz for ^1H and 125 MHz for ^{13}C)

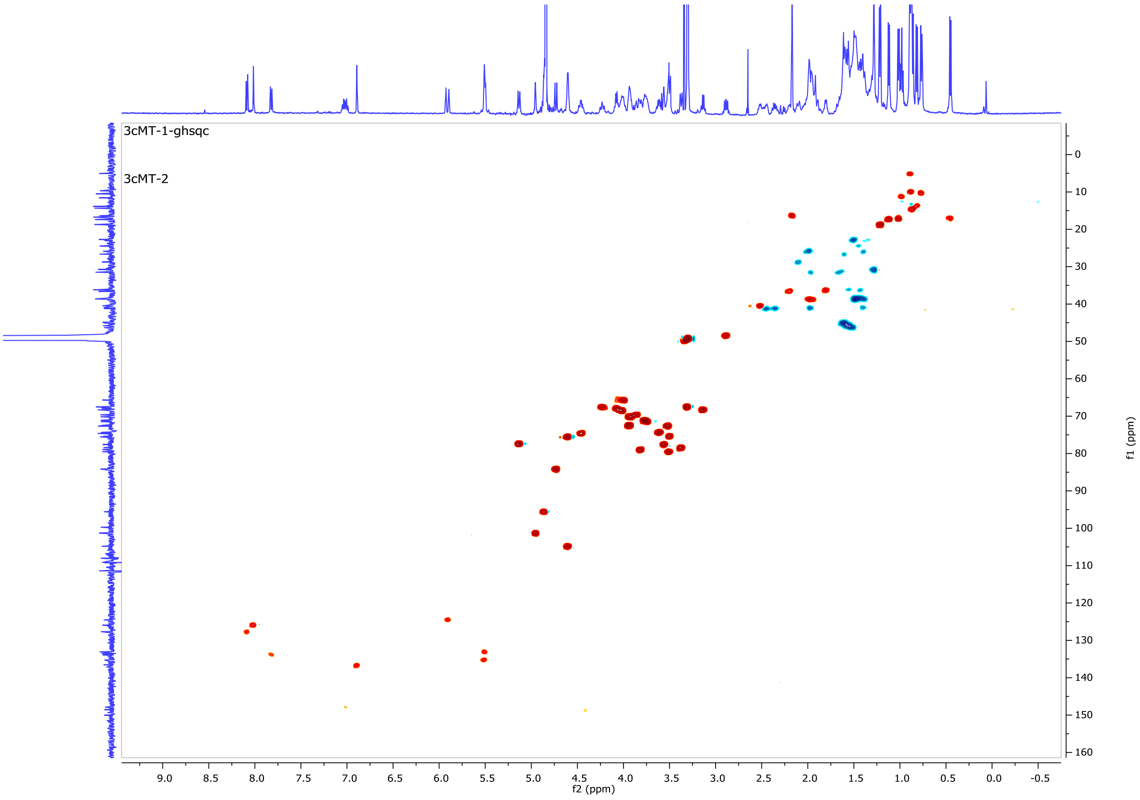

gHSQC spectrun of **3** (CD_3OD, 500 MHz for ^1H and 125 MHz for ^{13}C)

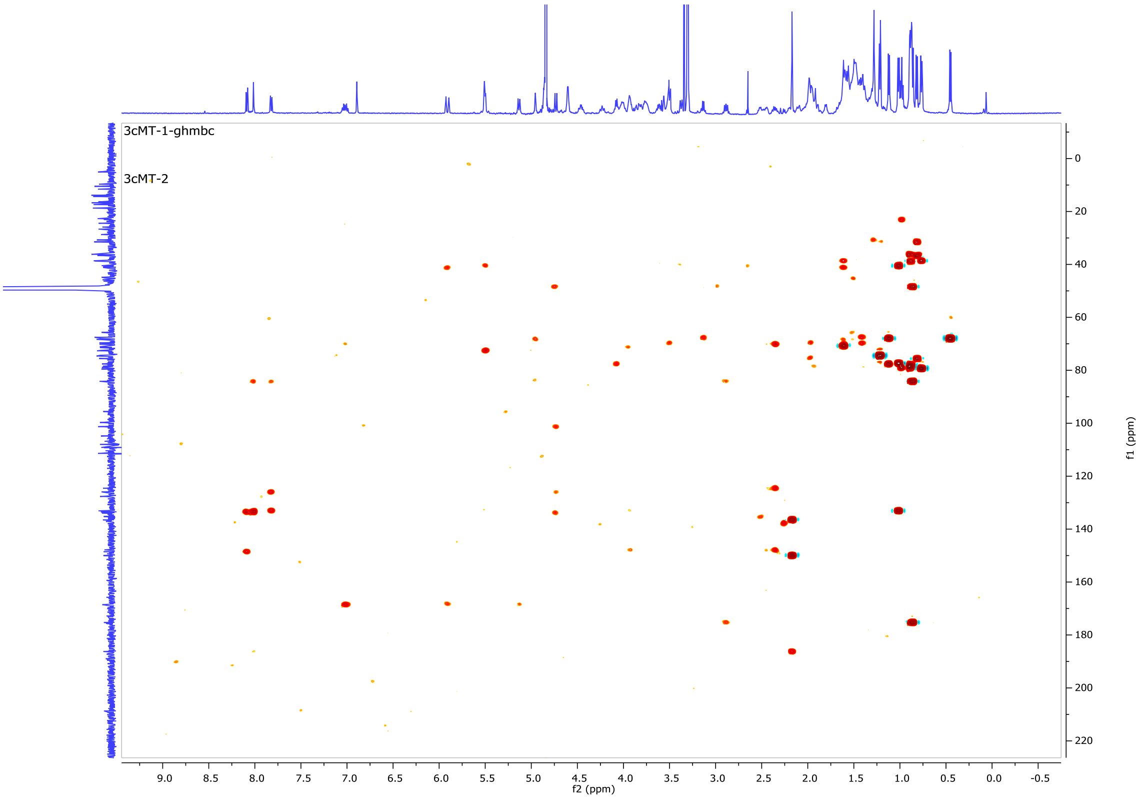

gHMBC spectrum of **3** (CD_3OD, 500 MHz for ^1H and 125 MHz for ^{13}C)

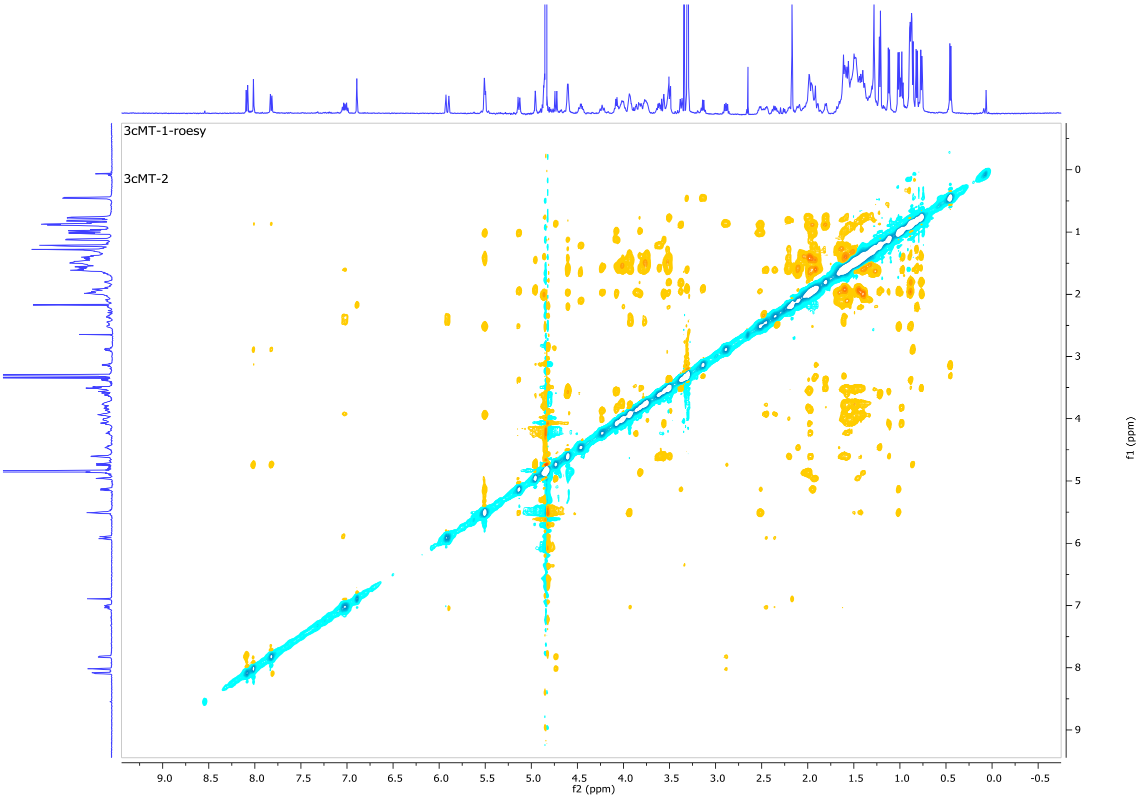

ROESY spectrum of **3** (CD_3OD, 500 MHz for ^1H and 125 MHz for ^{13}C)

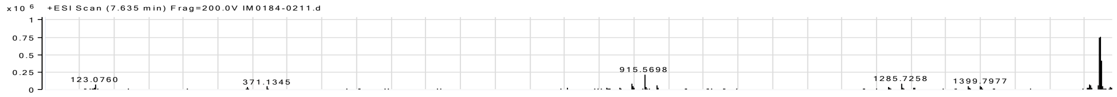

MS spectrum of **3**

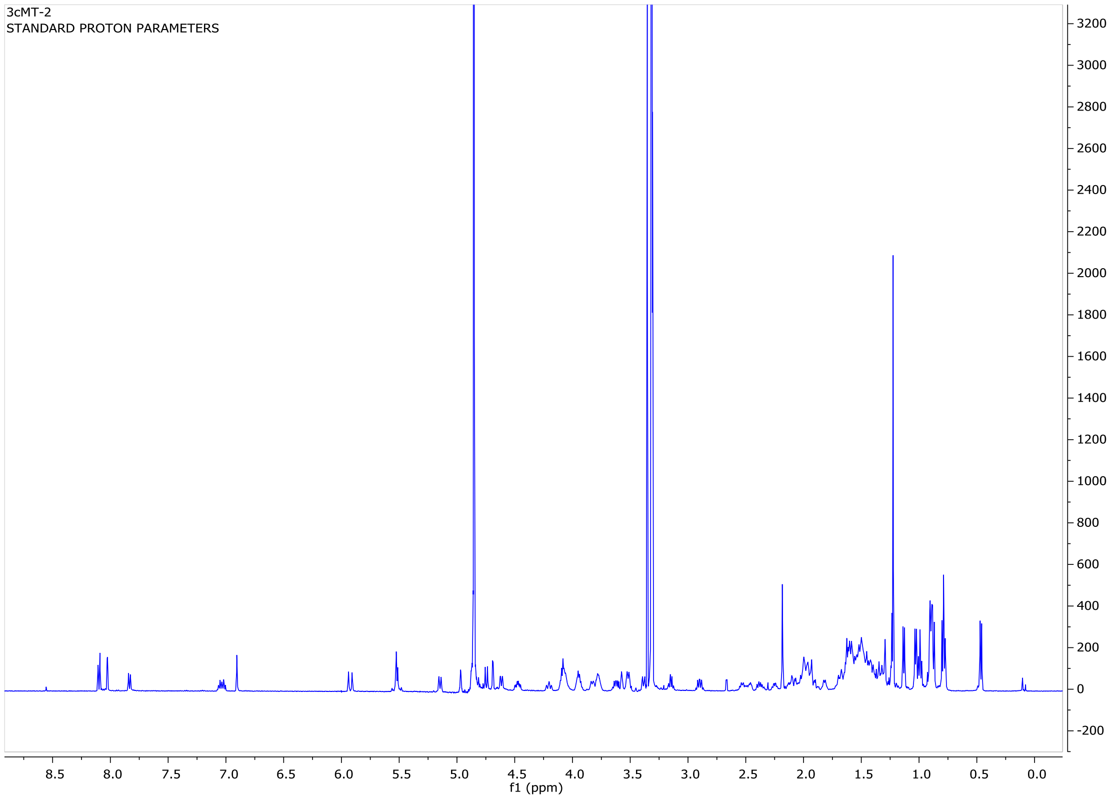

^{1}H spectrum of **4** (CD_3OD, 500 MHz for ^{1}H and 125 MHz for ^{13}C)

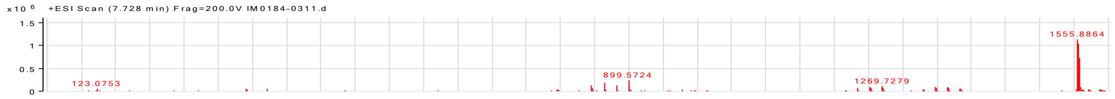

MS spectrum of **4**

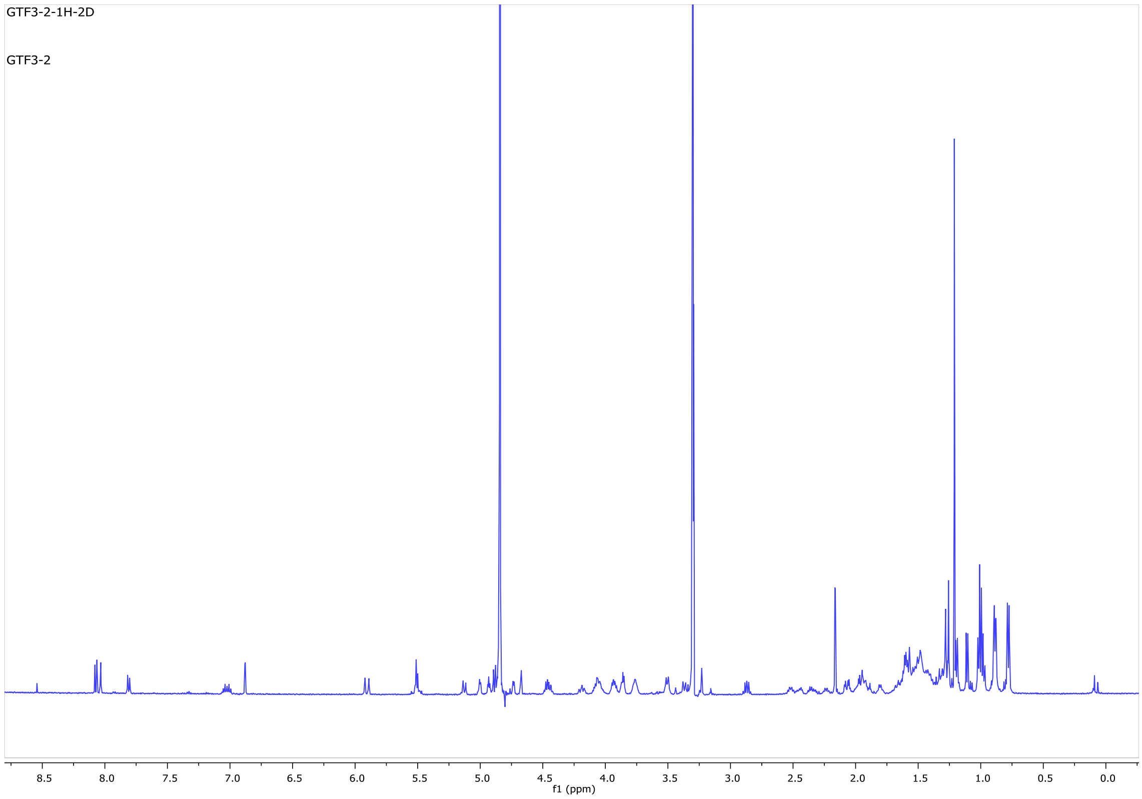

^1H spectrum of **5** (CD_3OD, 500 MHz for ^1H and 125 MHz for ^{13}C)

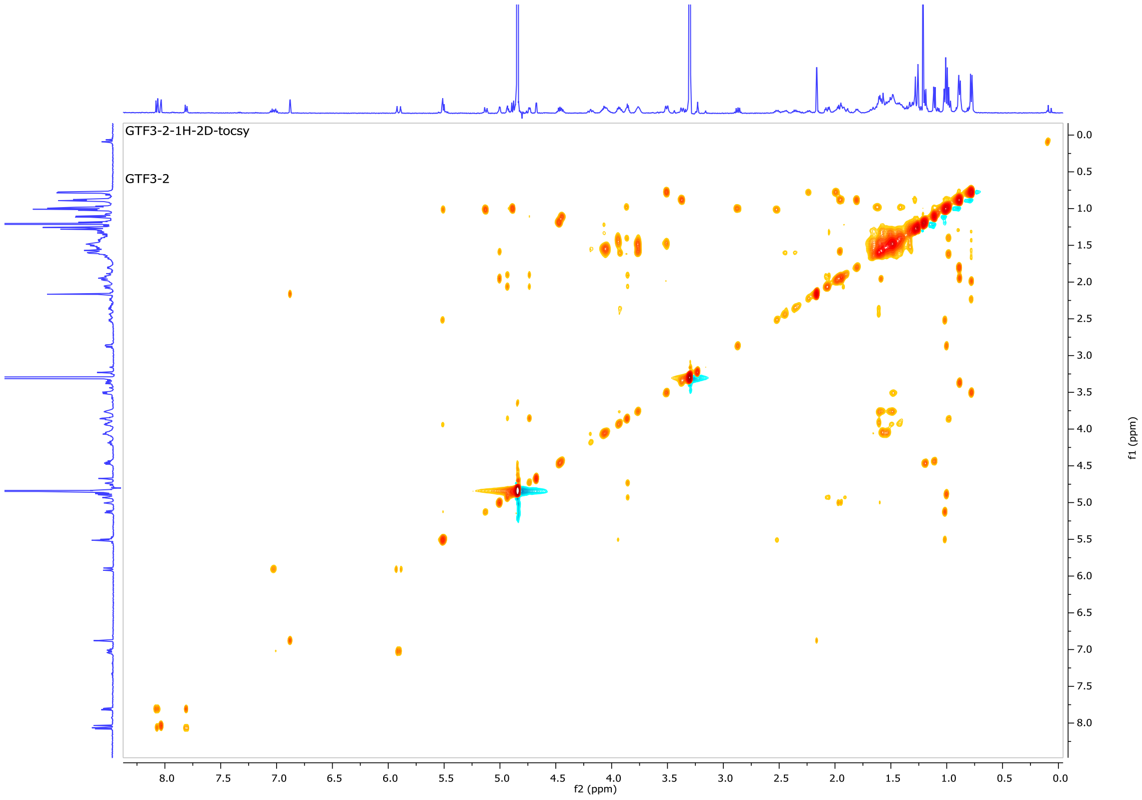

TOCSY spectrum of **5** (CD_3OD, 500 MHz for ^1H and 125 MHz for ^{13}C)

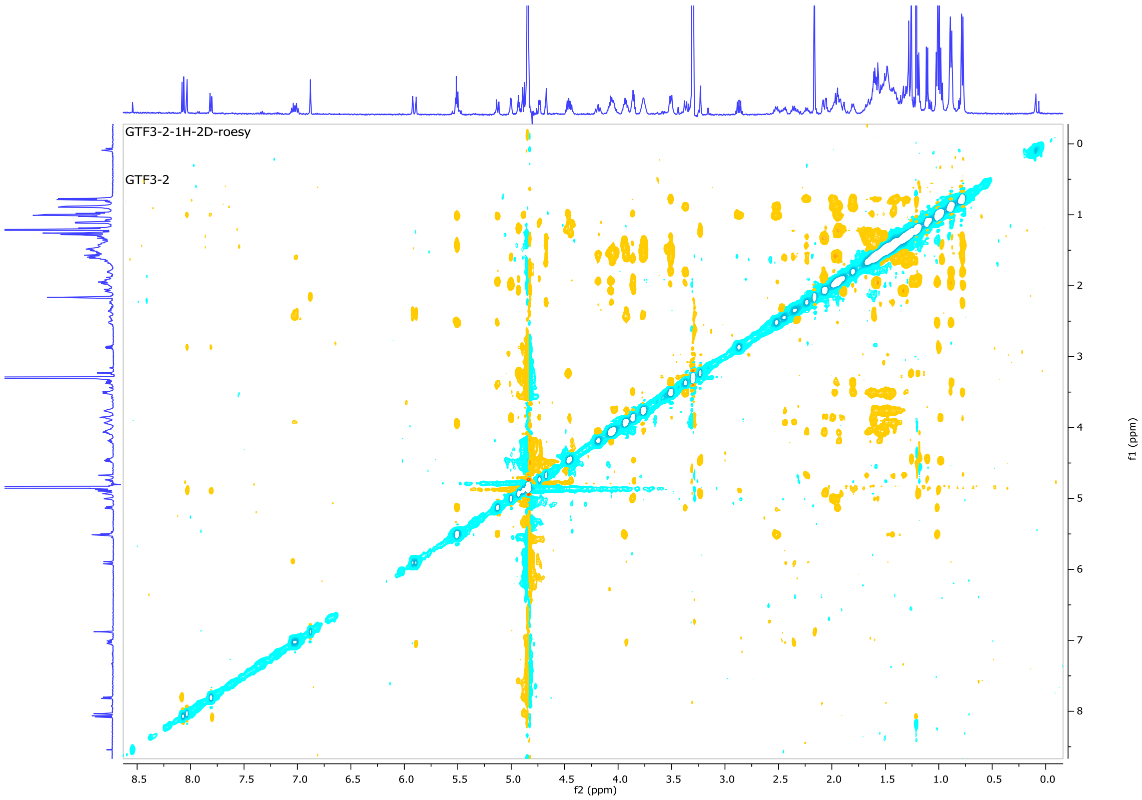

ROESY spectrum of **5** (CD_3OD, 500 MHz for ^1H and 125 MHz for ^{13}C)

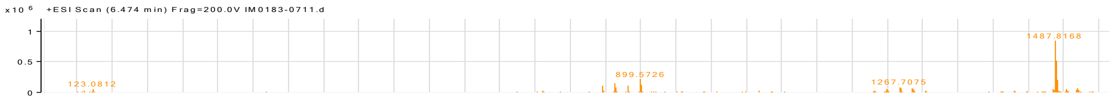

MS spectrum of **5**

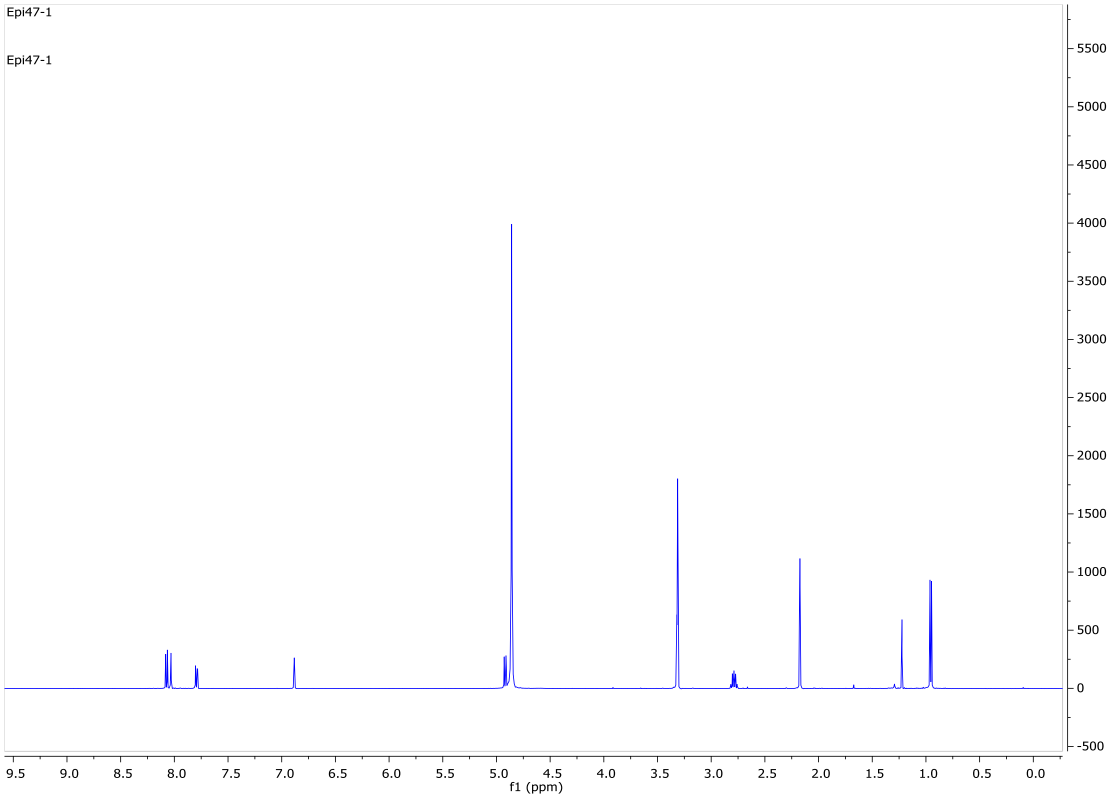

^{1}H spectrum of **6** (CD_3OD, 500 MHz for ^{1}H and 125 MHz for ^{13}C)

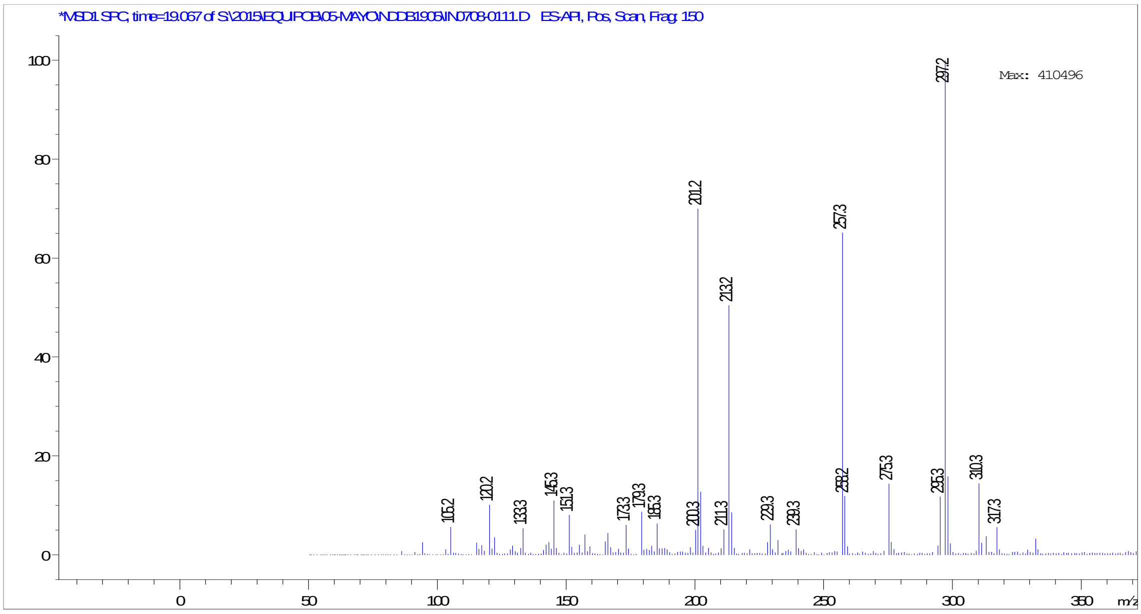

MS spectrum of **6**

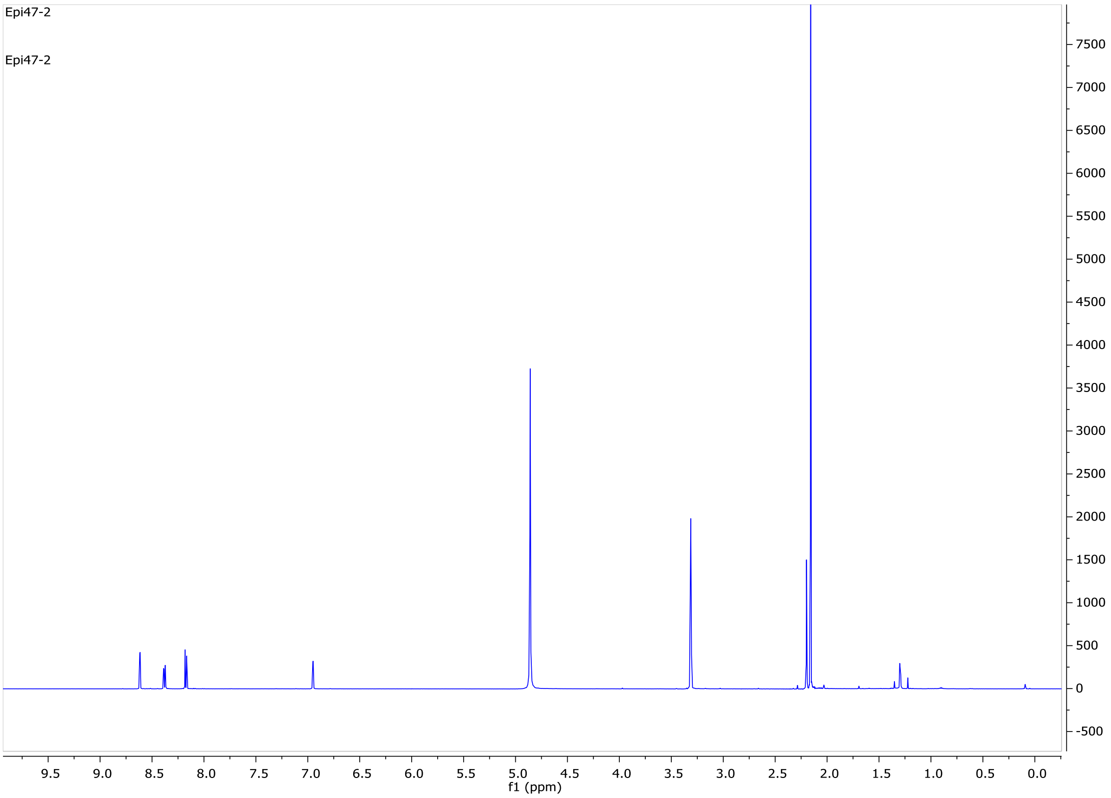

^{1}H spectrum of **7** (CD_3OD, 500 MHz for ^{1}H and 125 MHz for ^{13}C)

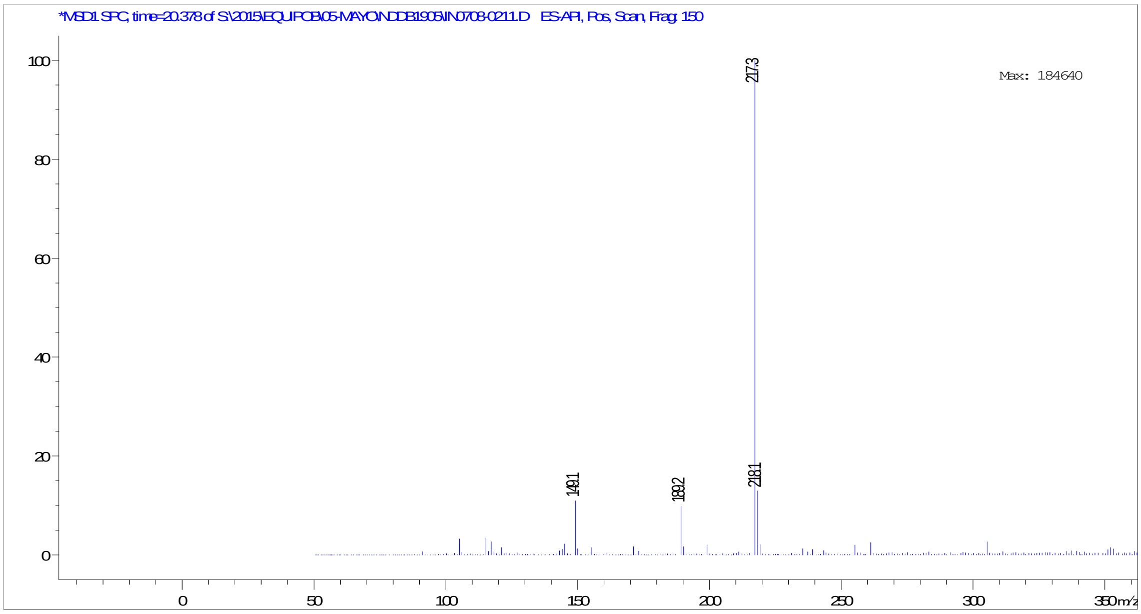

MS spectrum of **7**

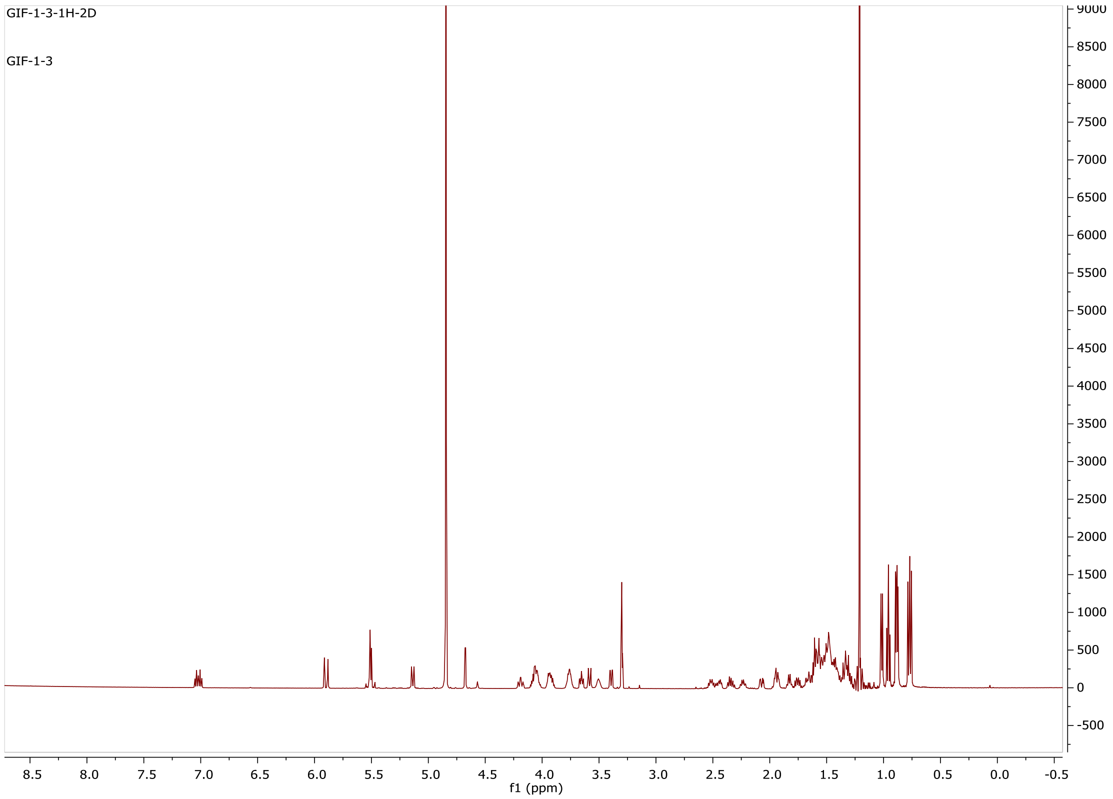

^{1}H spectrum of **10** (CD_3OD, 500 MHz for ^{1}H and 125 MHz for ^{13}C)

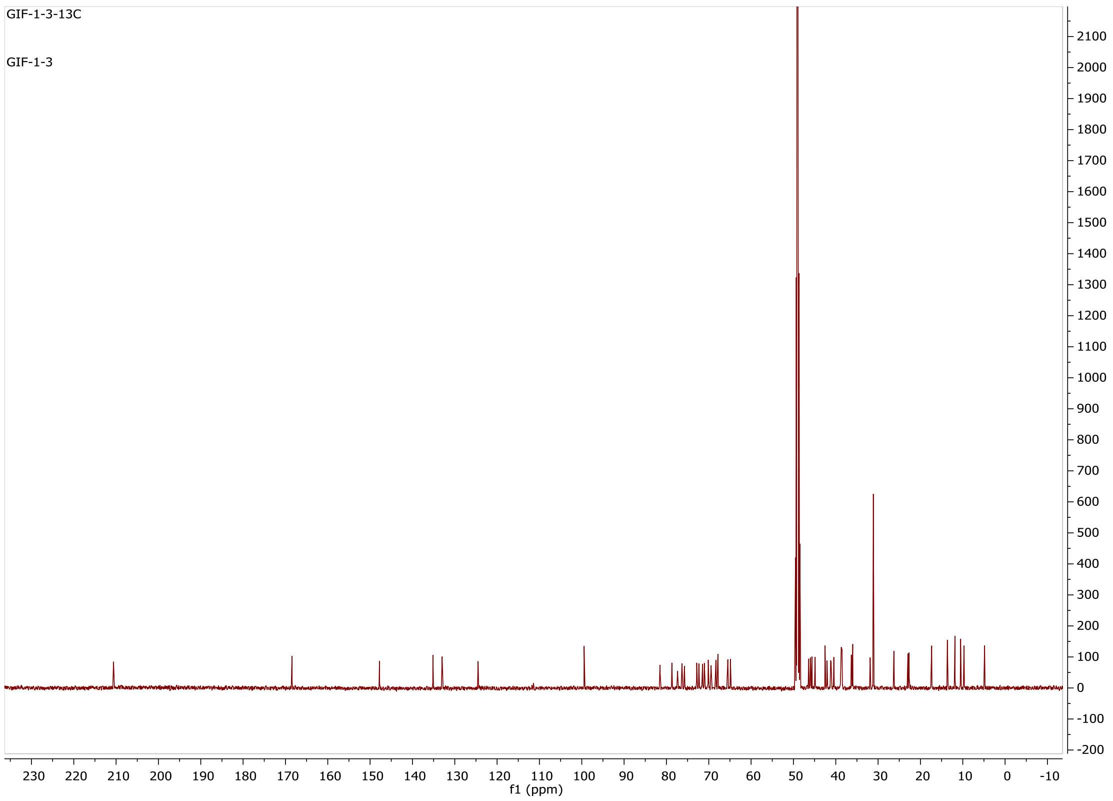

^{13}C spectrum of **10** (CD_3OD, 500 MHz for ^{1}H and 125 MHz for ^{13}C)

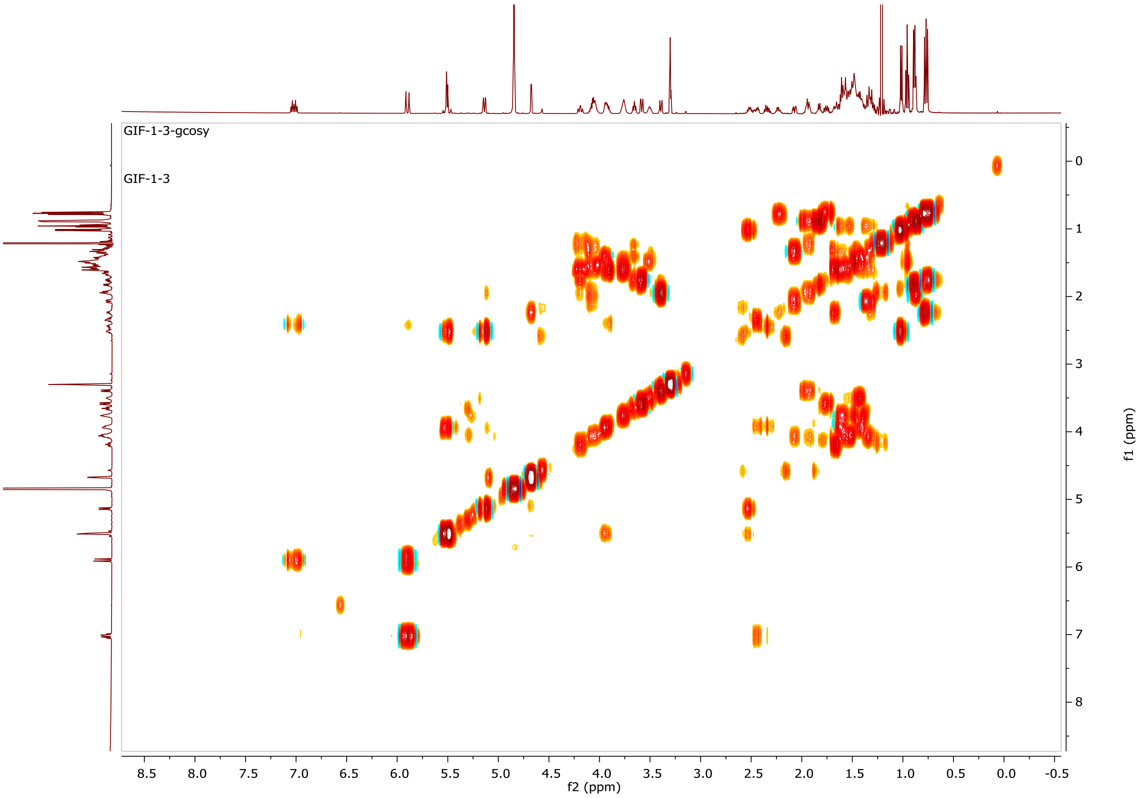

gCOSY spectrum of **10** (CD_3OD, 500 MHz for ^1H and 125 MHz for ^{13}C)

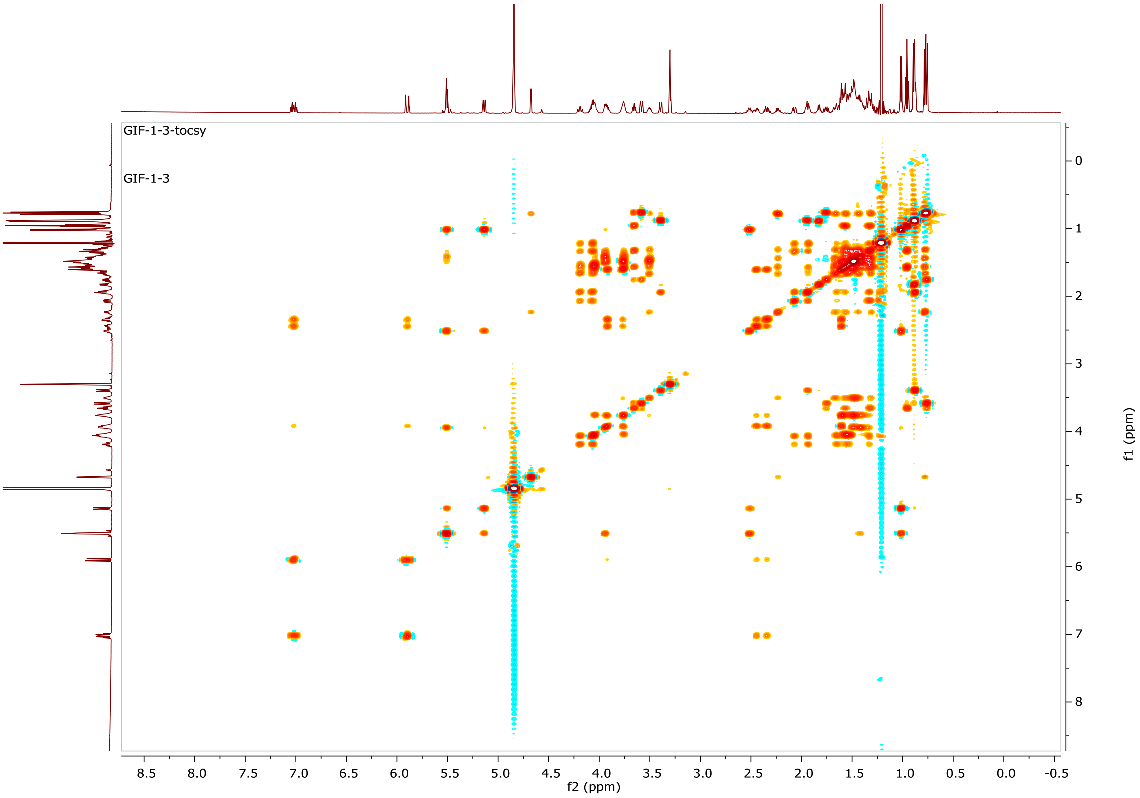

TOCSY spectrum of **10** (CD_3OD, 500 MHz for ^1H and 125 MHz for ^{13}C)

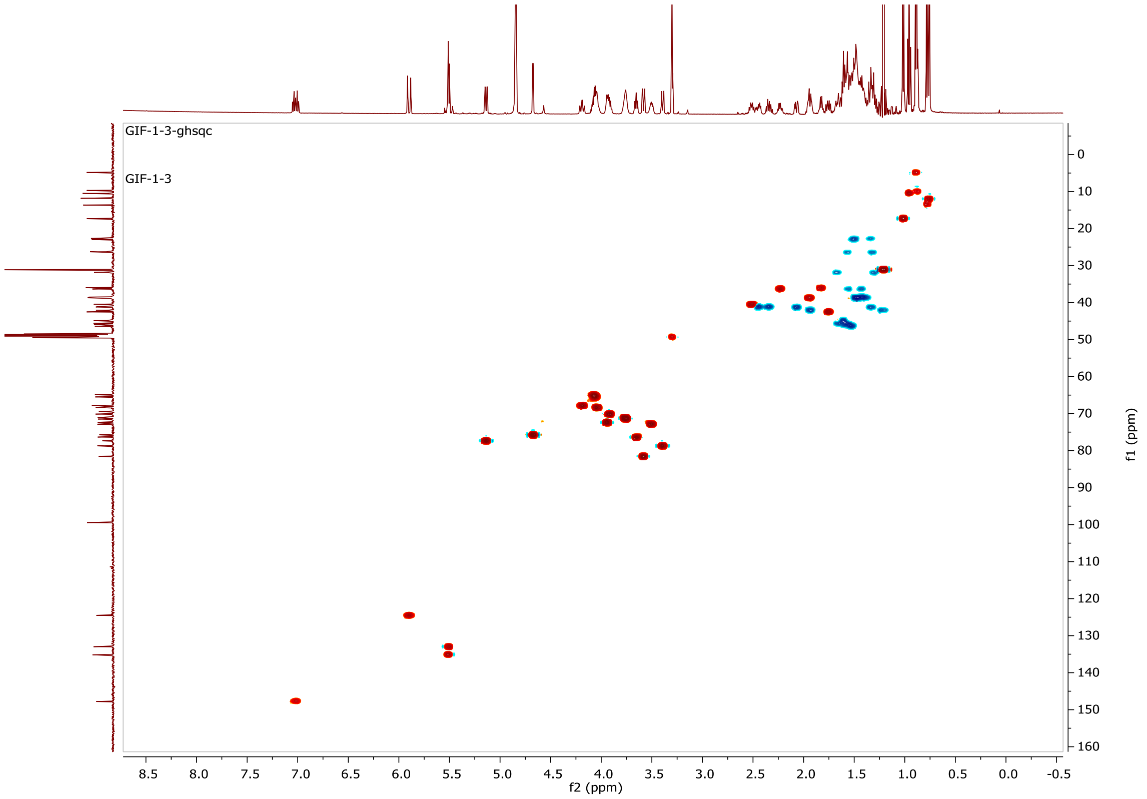

gHSQC spectrun of **10** (CD_3OD, 500 MHz for ^1H and 125 MHz for ^{13}C)

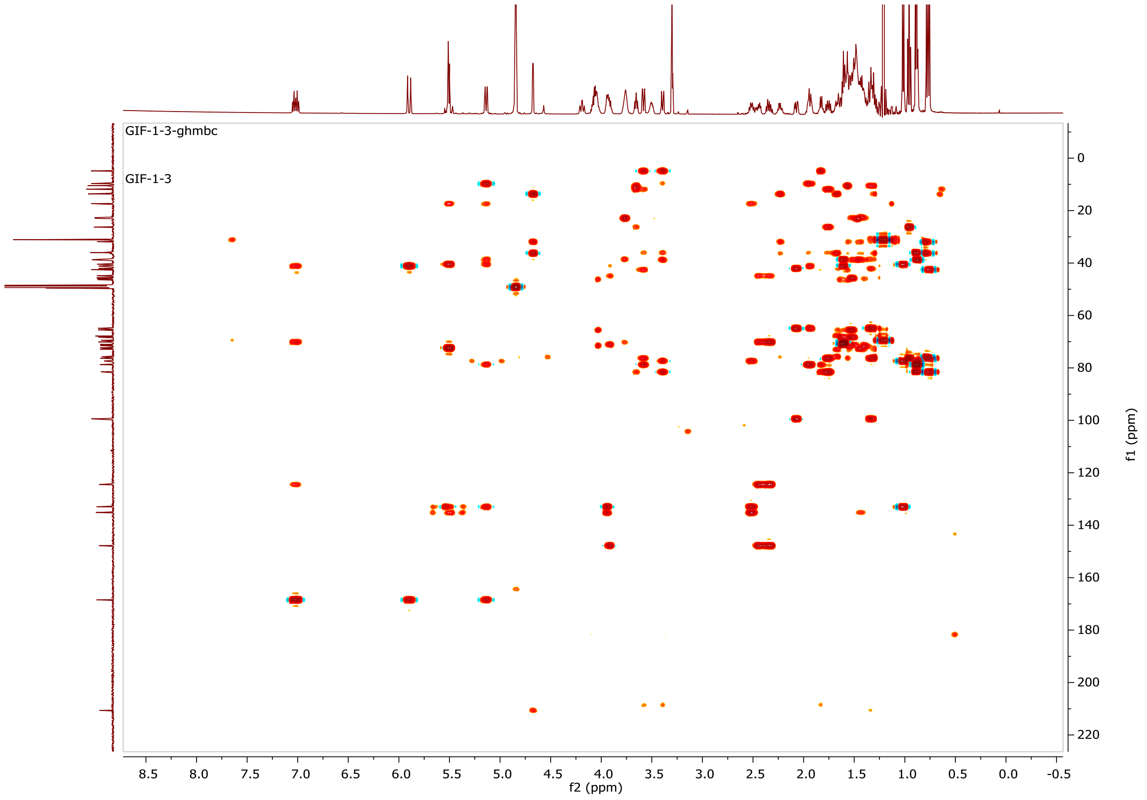

gHMBC spectrum of **10** (CD_3OD, 500 MHz for ^1H and 125 MHz for ^{13}C)

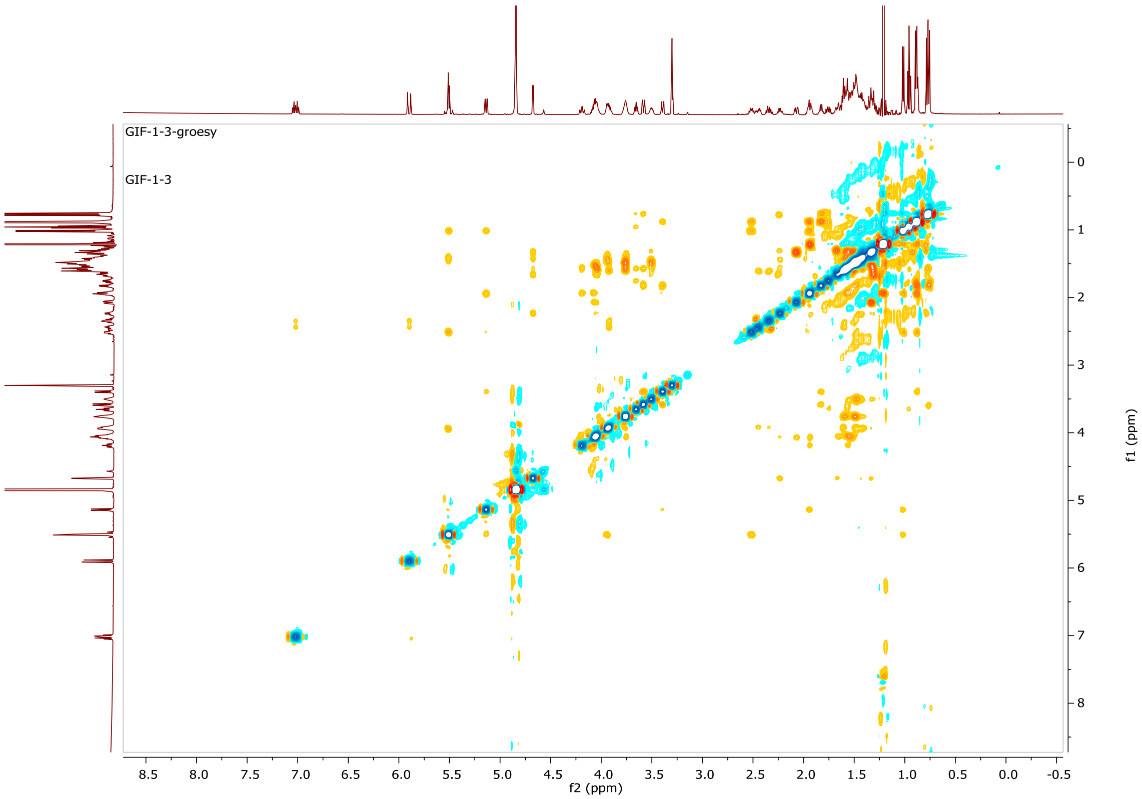

ROESY spectrum of **10** (CD_3OD, 500 MHz for ^1H and 125 MHz for ^{13}C)

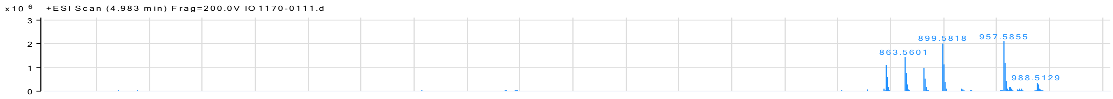

MS spectrum of **10**

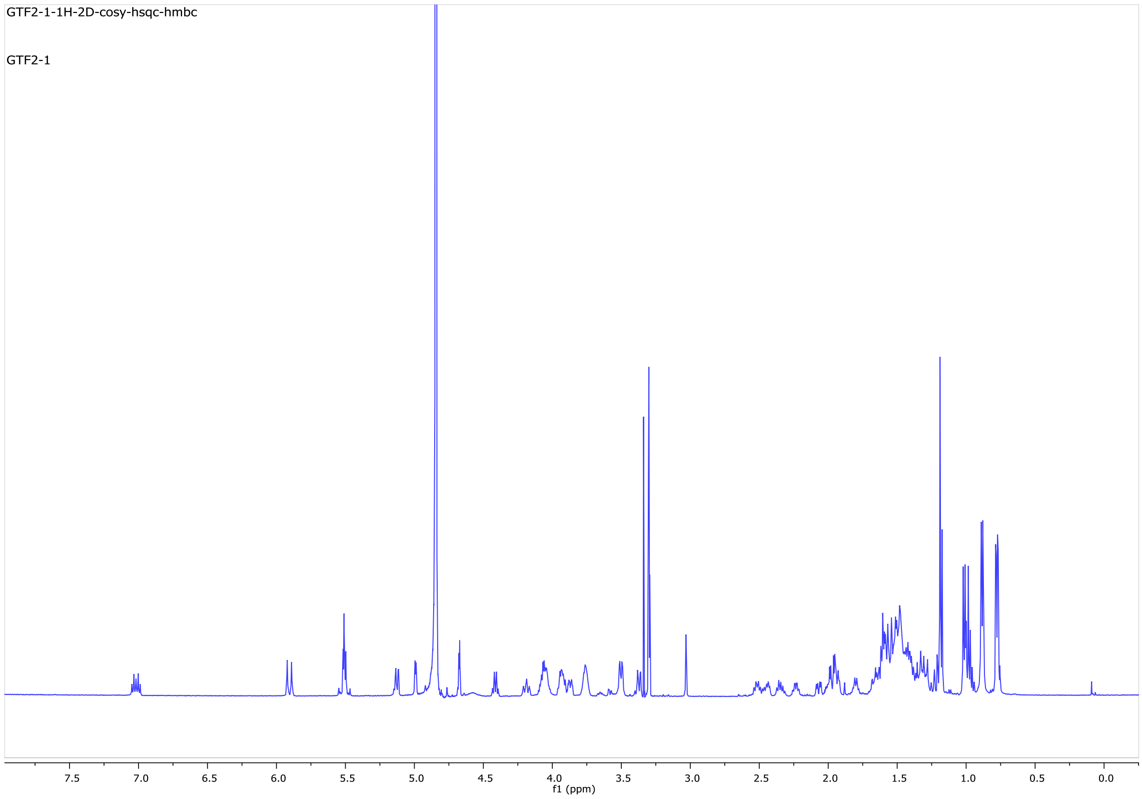

^{1}H spectrum of **11** (CD_3OD, 500 MHz for ^{1}H and 125 MHz for ^{13}C)

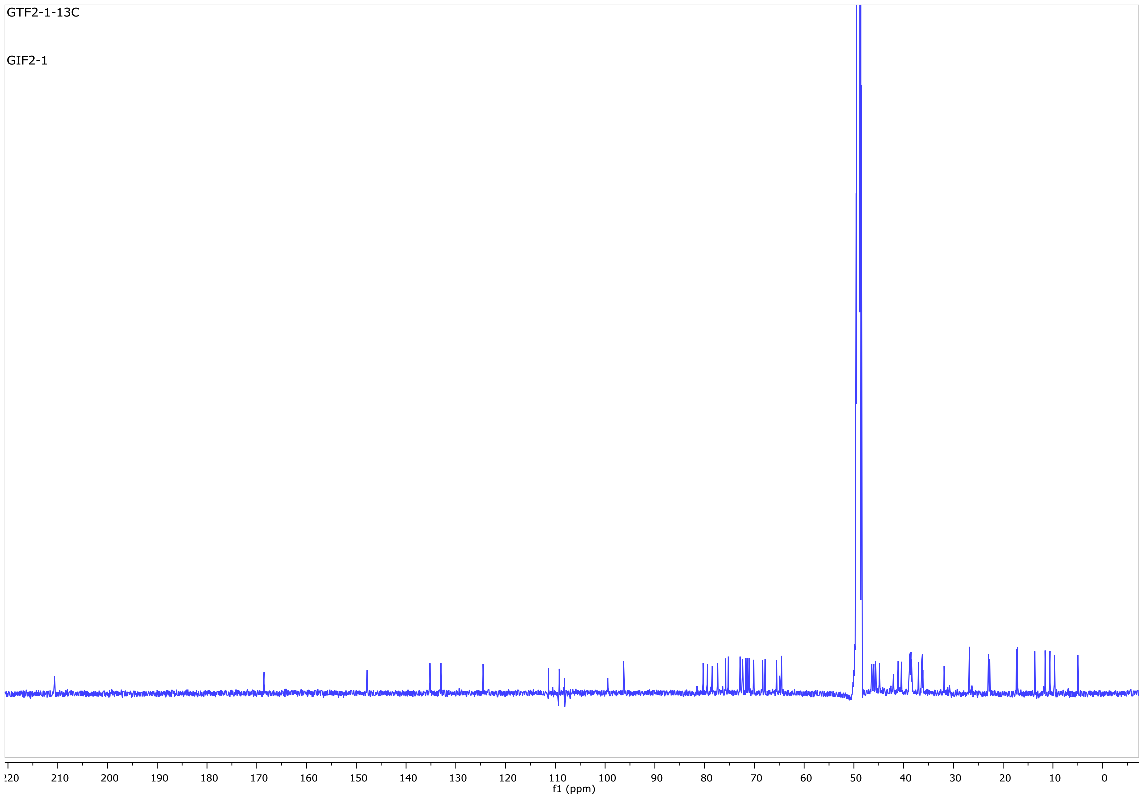

^{13}C spectrum of **11** (CD_3OD, 500 MHz for ^{1}H and 125 MHz for ^{13}C)

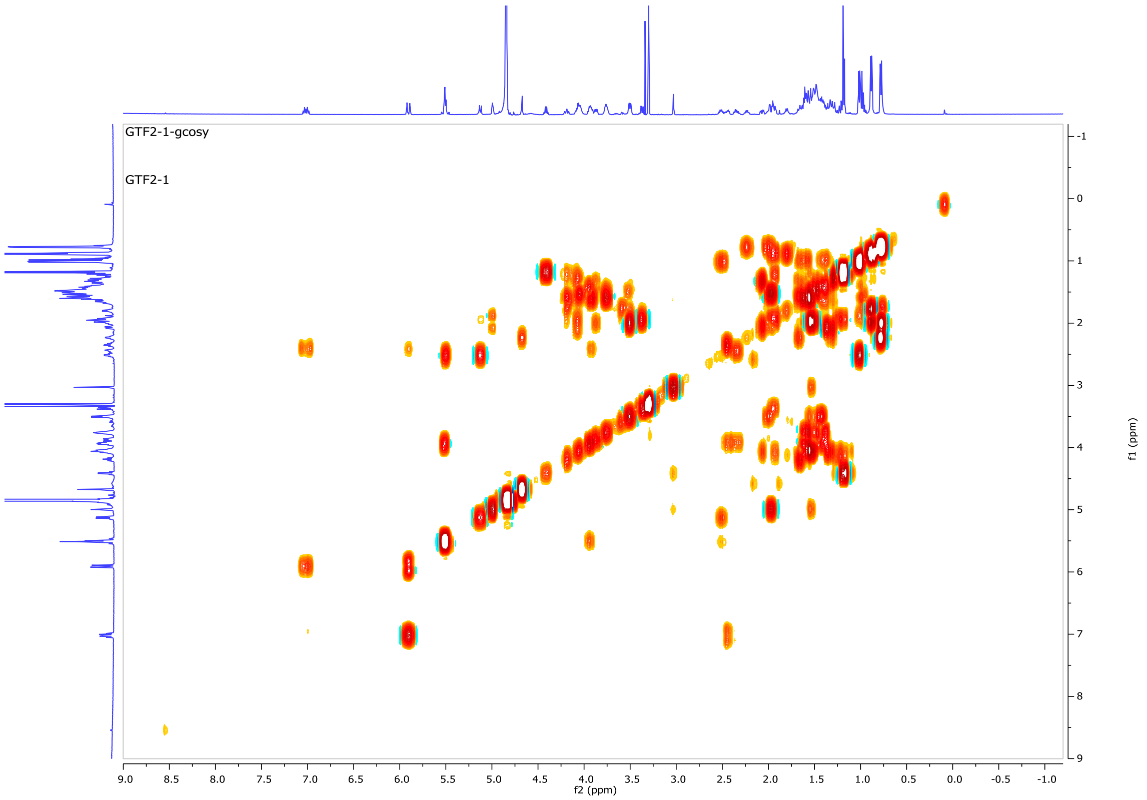

gCOSY spectrum of **11** (CD_3OD, 500 MHz for ^1H and 125 MHz for ^{13}C)

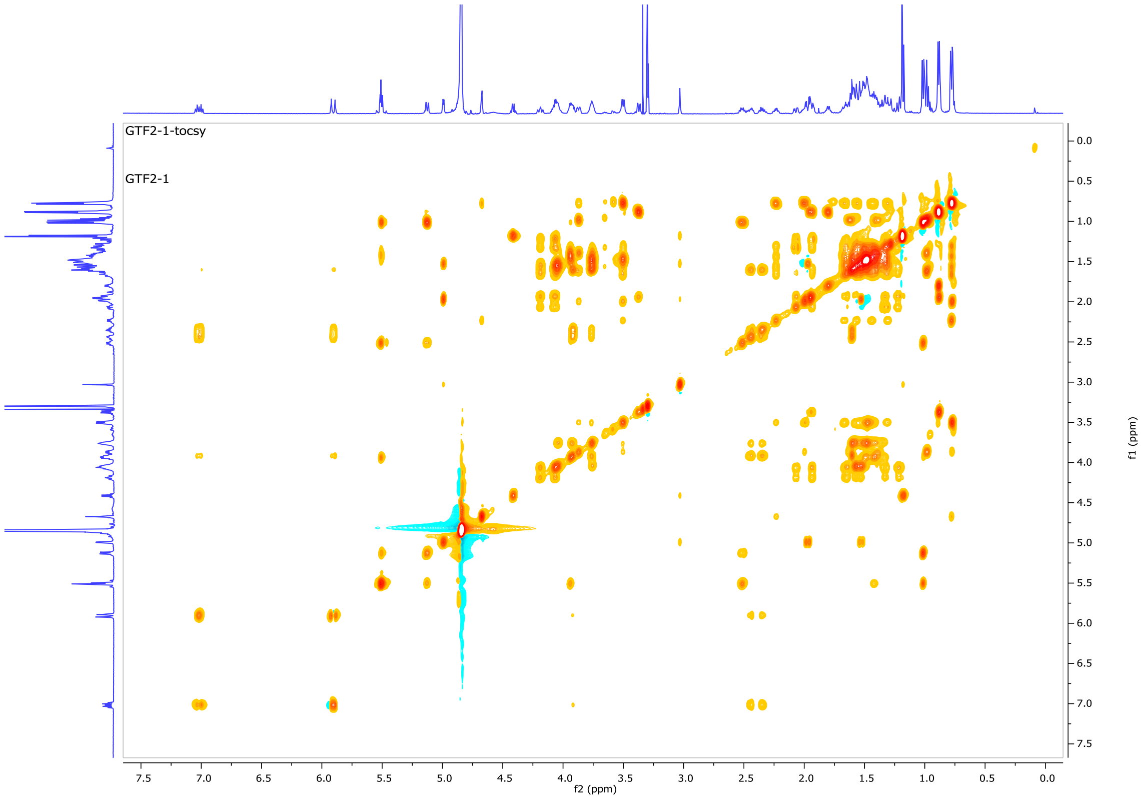

TOCSY spectrum of **11** (CD_3OD, 500 MHz for ^1H and 125 MHz for ^{13}C)

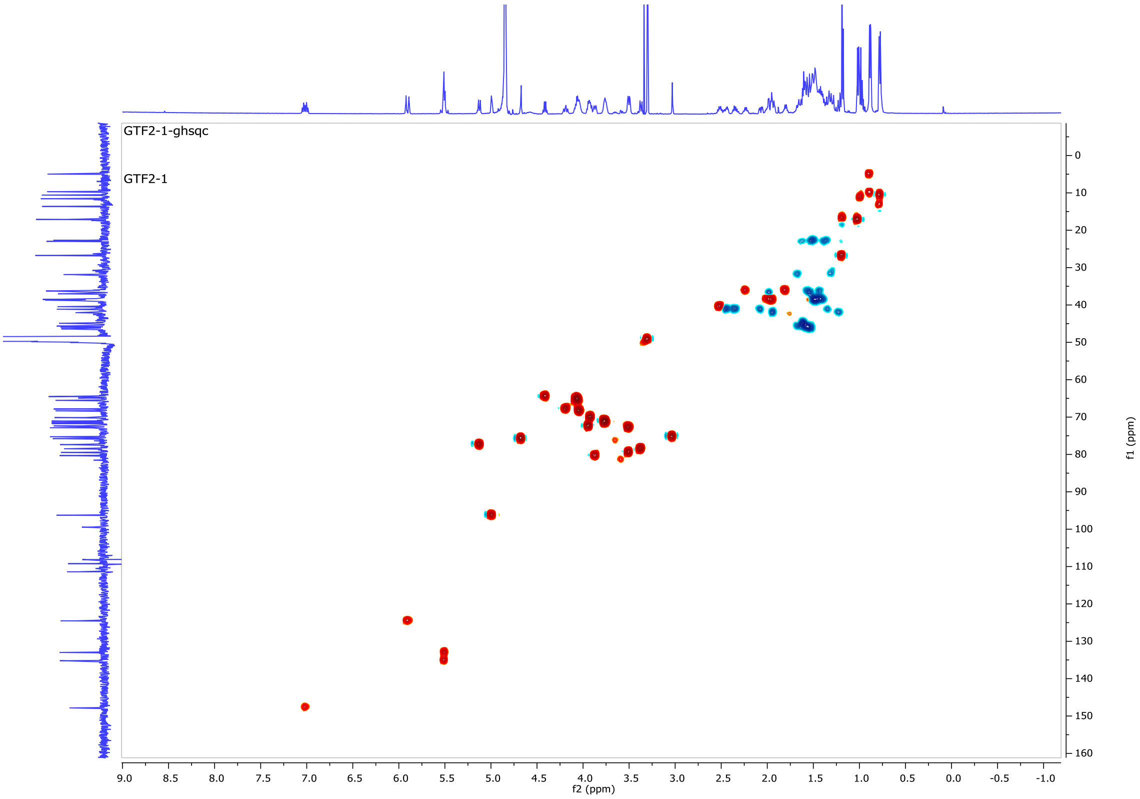

gHSQC spectrun of **11** (CD_3OD, 500 MHz for ^1H and 125 MHz for ^{13}C)

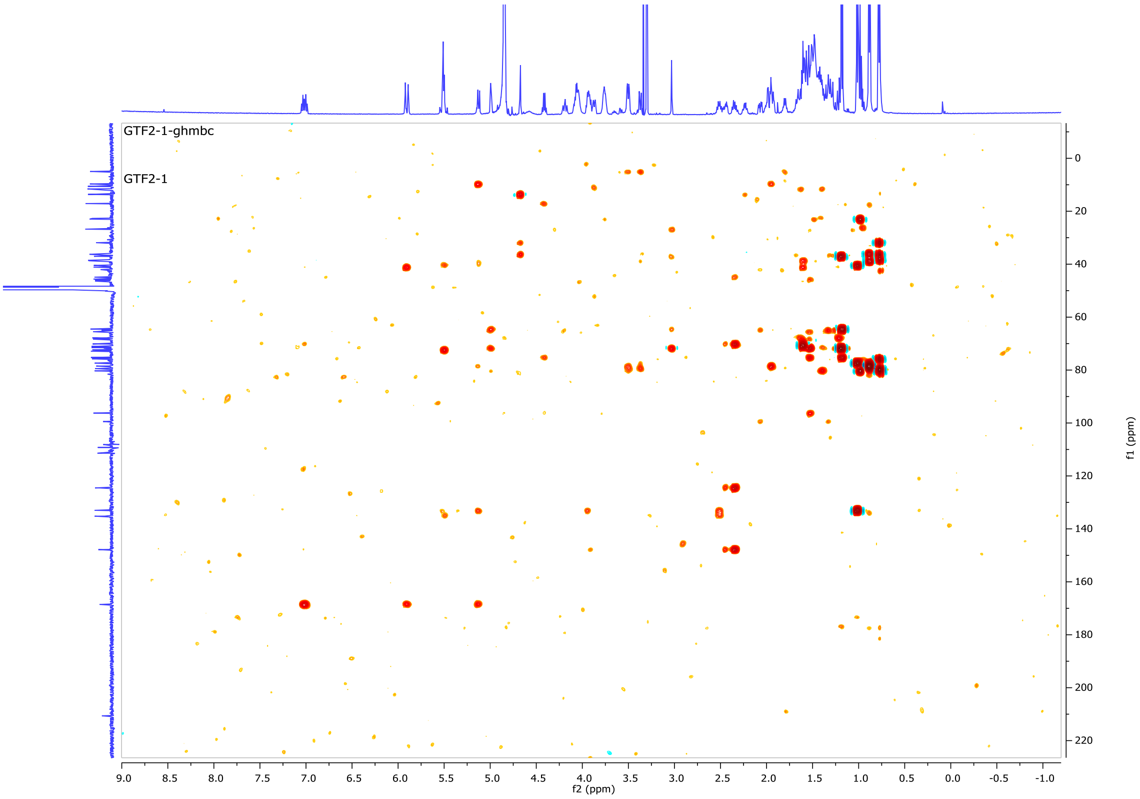

gHMBC spectrum of **11** (CD_3OD, 500 MHz for ^1H and 125 MHz for ^{13}C)

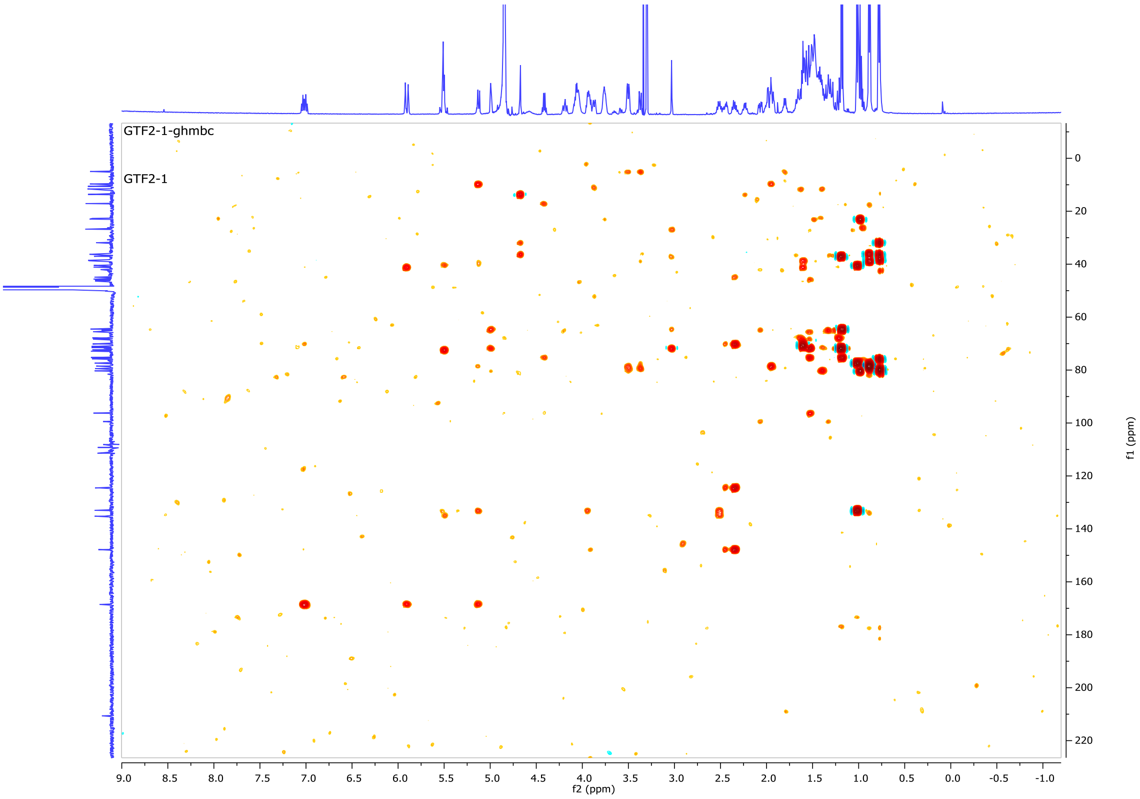

ROESY spectrum of **11** (CD_3OD, 500 MHz for ^1H and 125 MHz for ^{13}C)

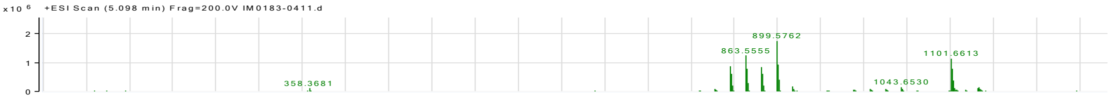

MS spectrum of **11**

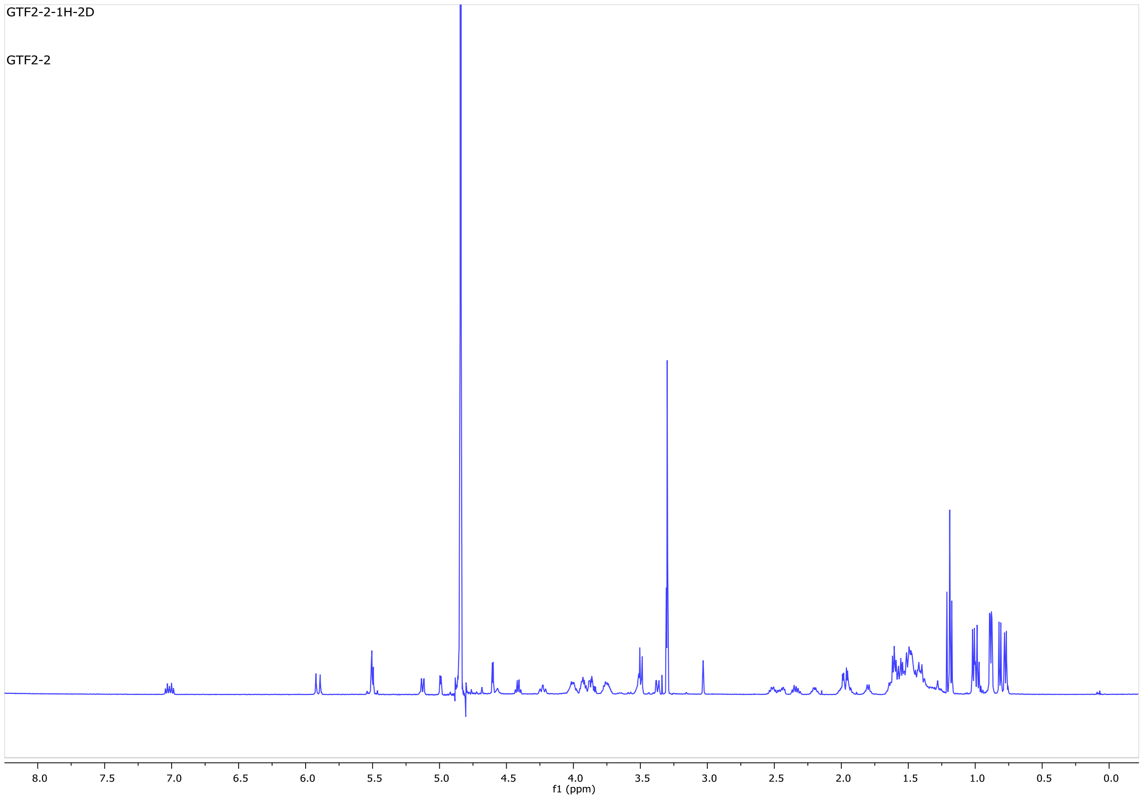

^{1}H spectrum of **12** (CD_3OD, 500 MHz for ^{1}H and 125 MHz for ^{13}C)

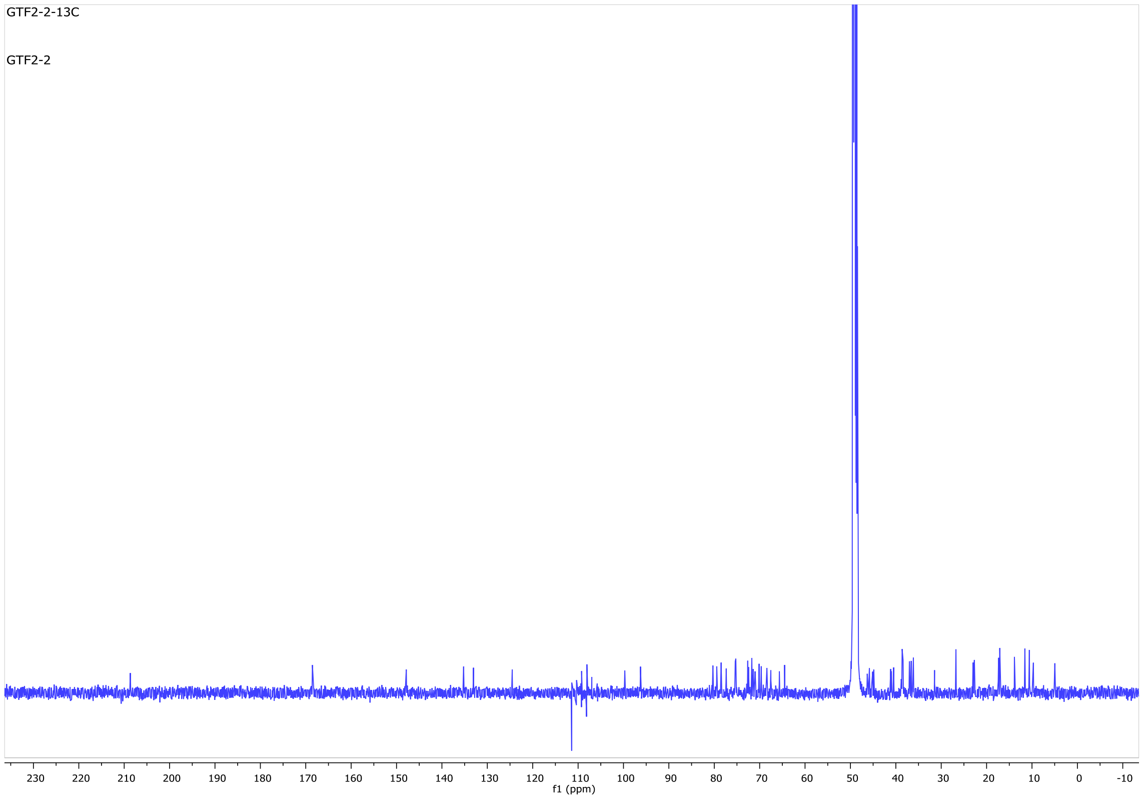

^{13}C spectrum of **12** (CD_3OD, 500 MHz for ^{1}H and 125 MHz for ^{13}C)

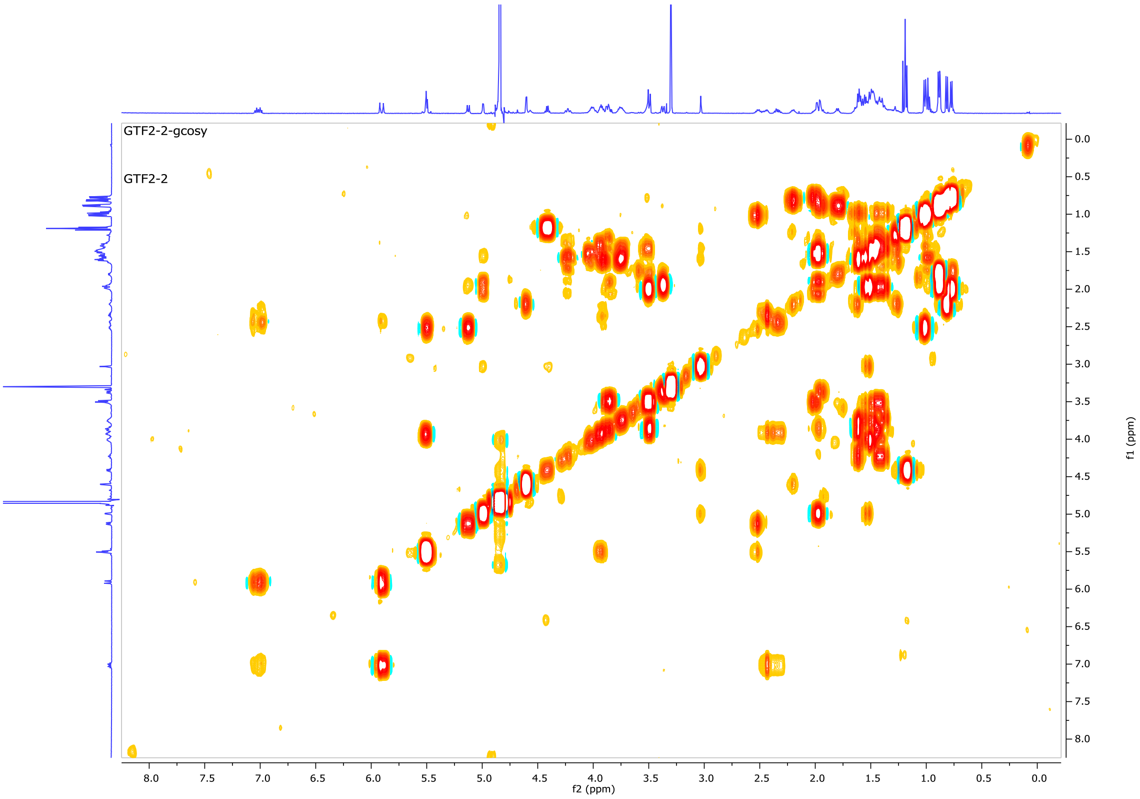

gCOSY spectrum of **12** (CD_3OD, 500 MHz for ^1H and 125 MHz for ^{13}C)

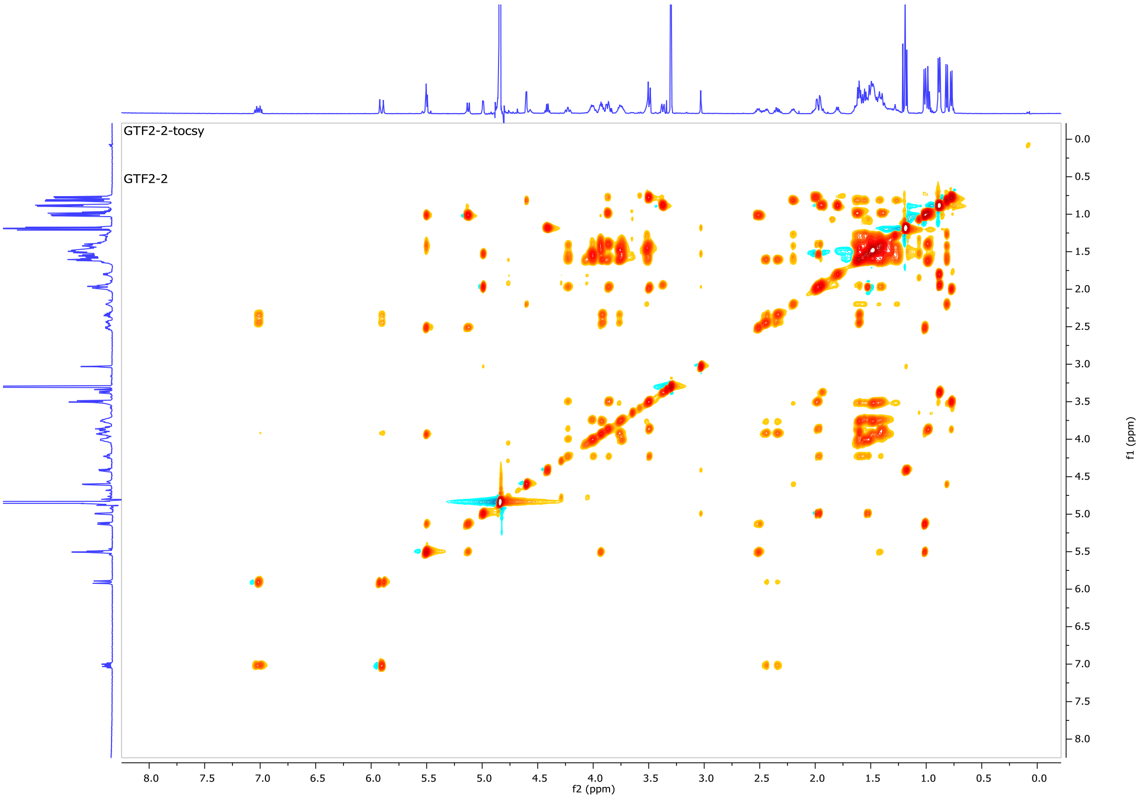

TOCSY spectrum of **12** (CD_3OD, 500 MHz for ^1H and 125 MHz for ^{13}C)

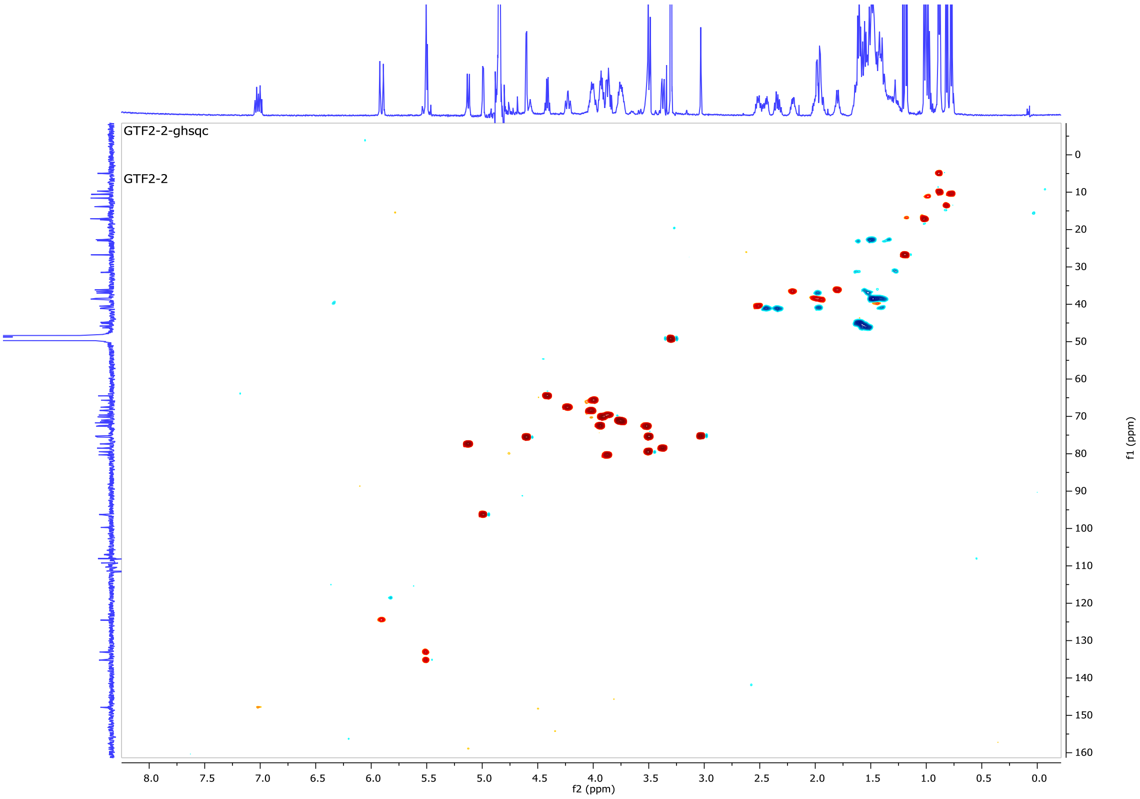

gHSQC spectrun of **12** (CD_3OD, 500 MHz for ^1H and 125 MHz for ^{13}C)

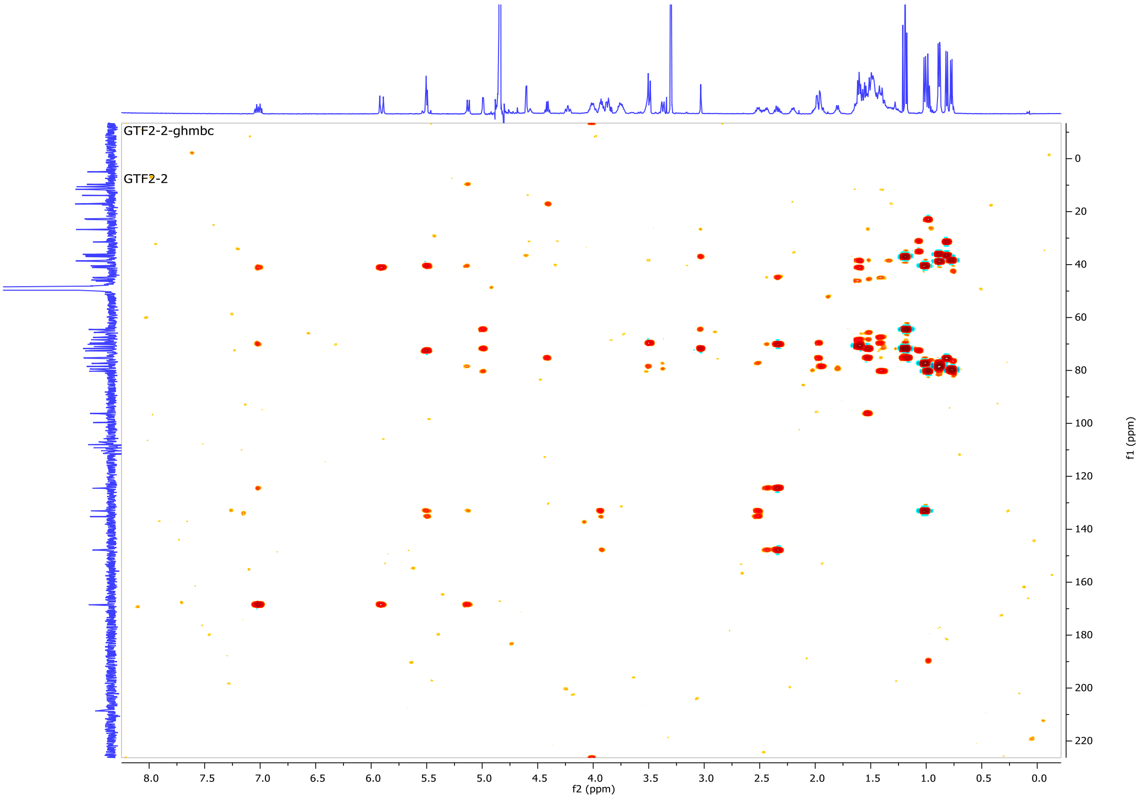

gHMBC spectrum of **12** (CD_3OD, 500 MHz for ^1H and 125 MHz for ^{13}C)

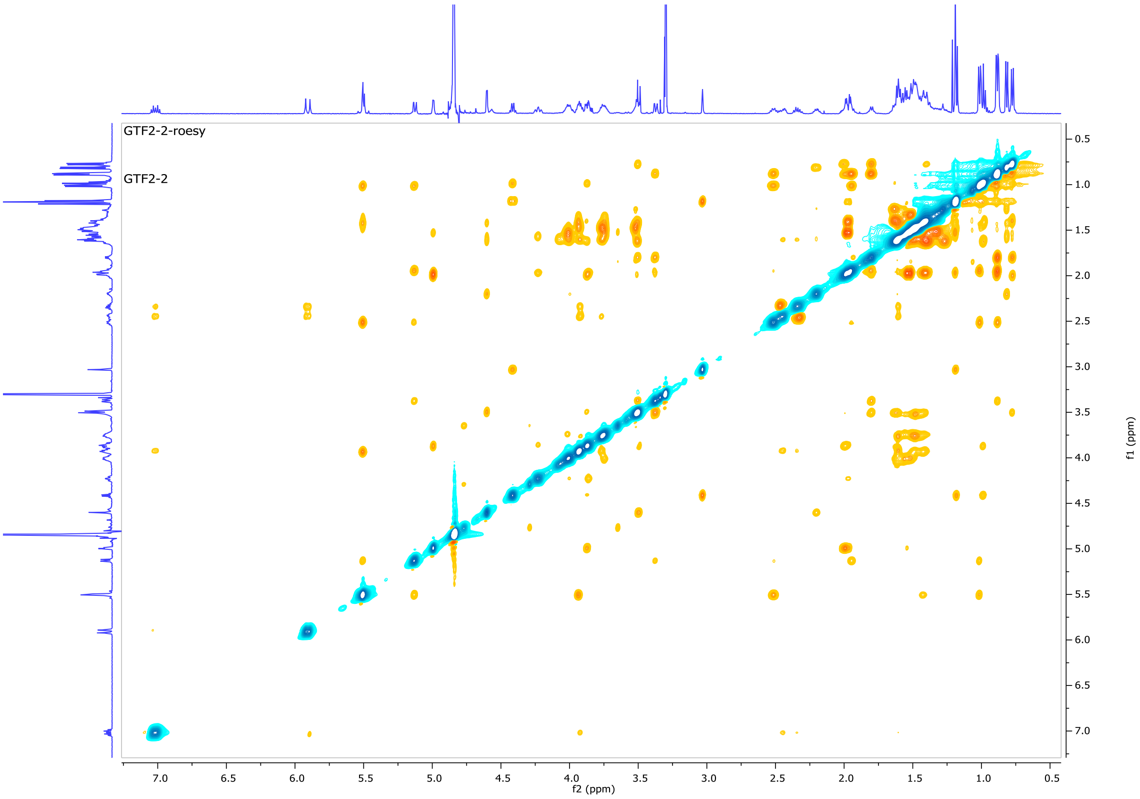

ROESY spectrum of **12** (CD_3OD, 500 MHz for ^1H and 125 MHz for ^{13}C)

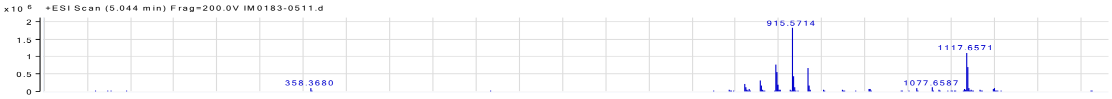

MS spectrum of **12**

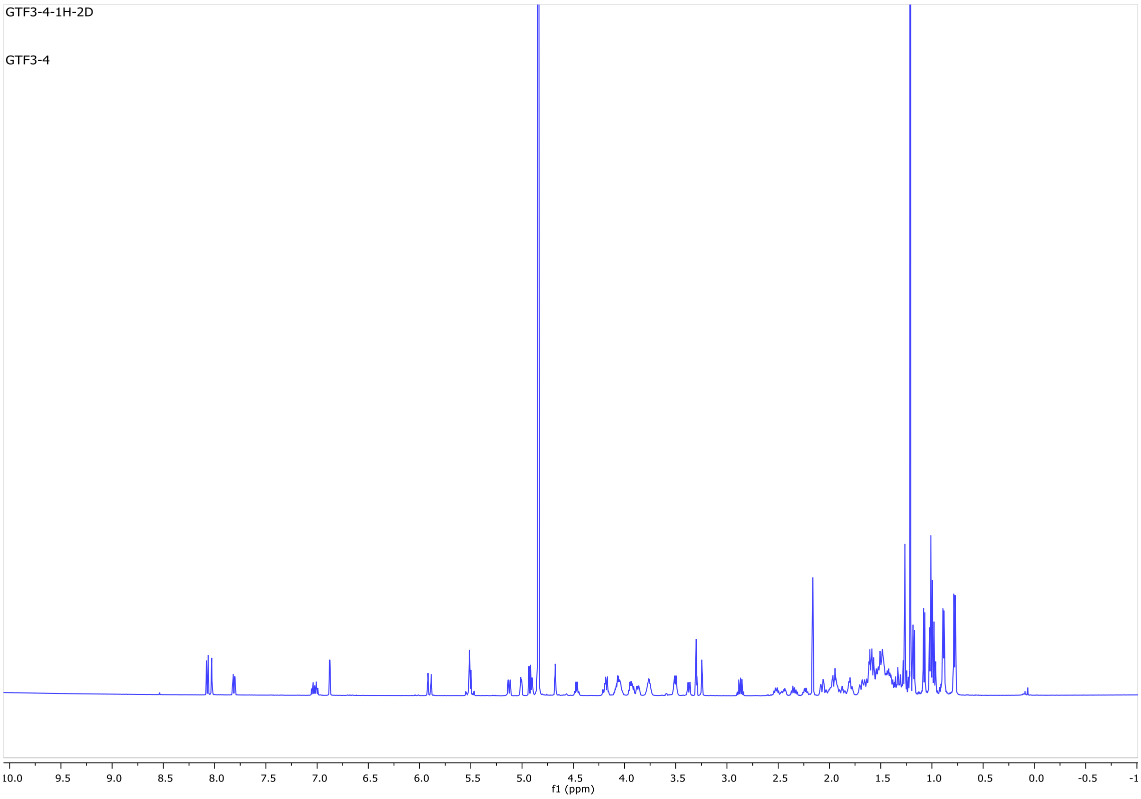

^{1}H spectrum of **13** (CD_3OD, 500 MHz for ^{1}H and 125 MHz for ^{13}C)

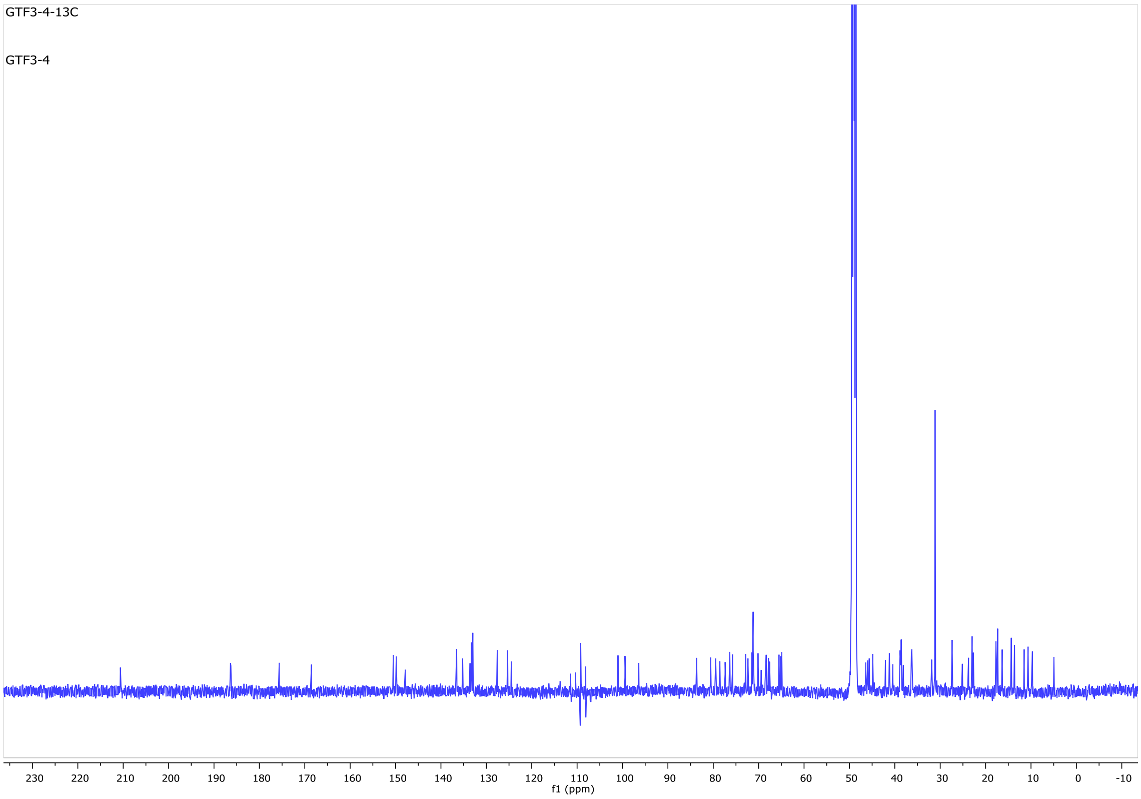

^{13}C spectrum of **13** (CD_3OD, 500 MHz for ^{1}H and 125 MHz for ^{13}C)

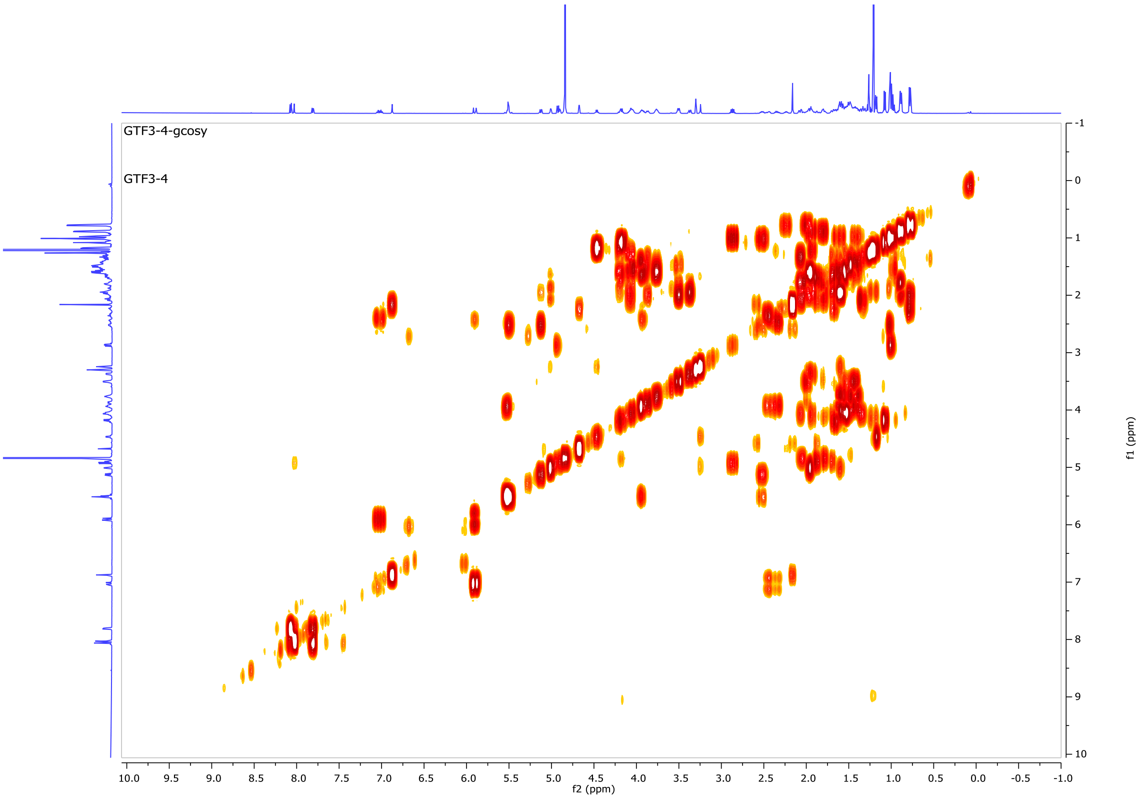

gCOSY spectrum of **13** (CD_3OD, 500 MHz for ^1H and 125 MHz for ^{13}C)

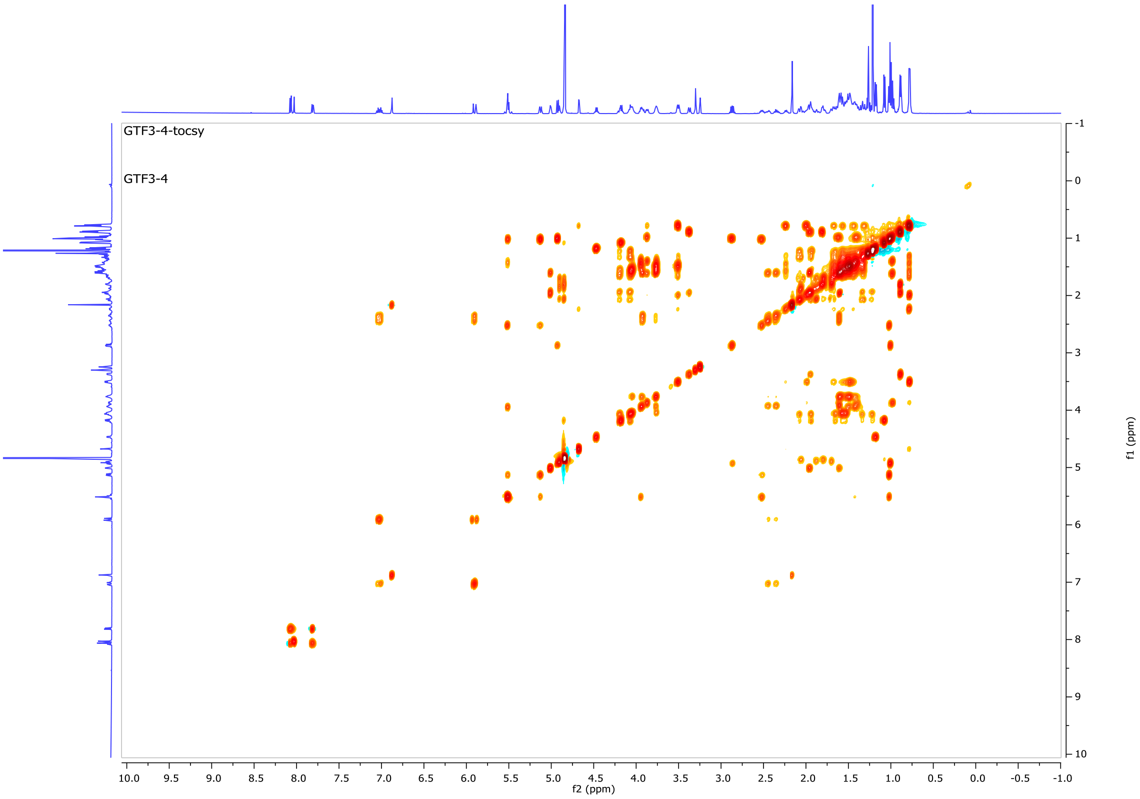

TOCSY spectrum of **13** (CD_3OD, 500 MHz for ^1H and 125 MHz for ^{13}C)

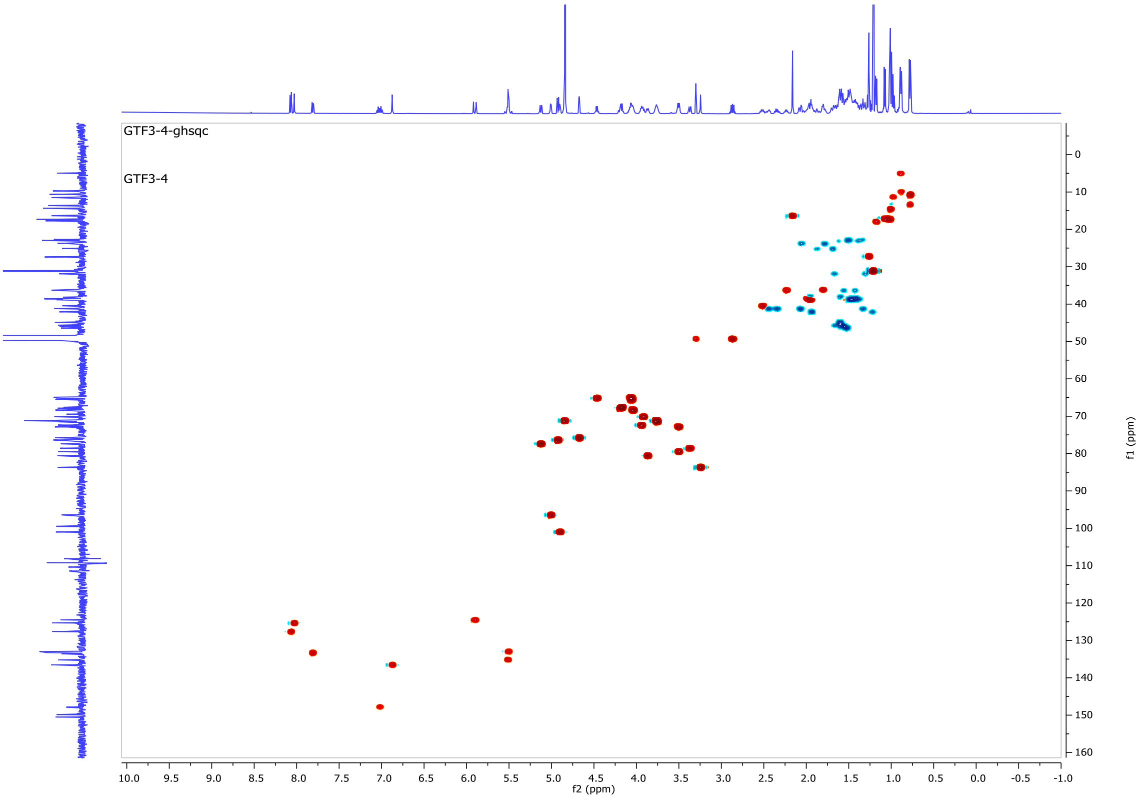

gHSQC spectrun of **13** (CD_3OD, 500 MHz for ^1H and 125 MHz for ^{13}C)

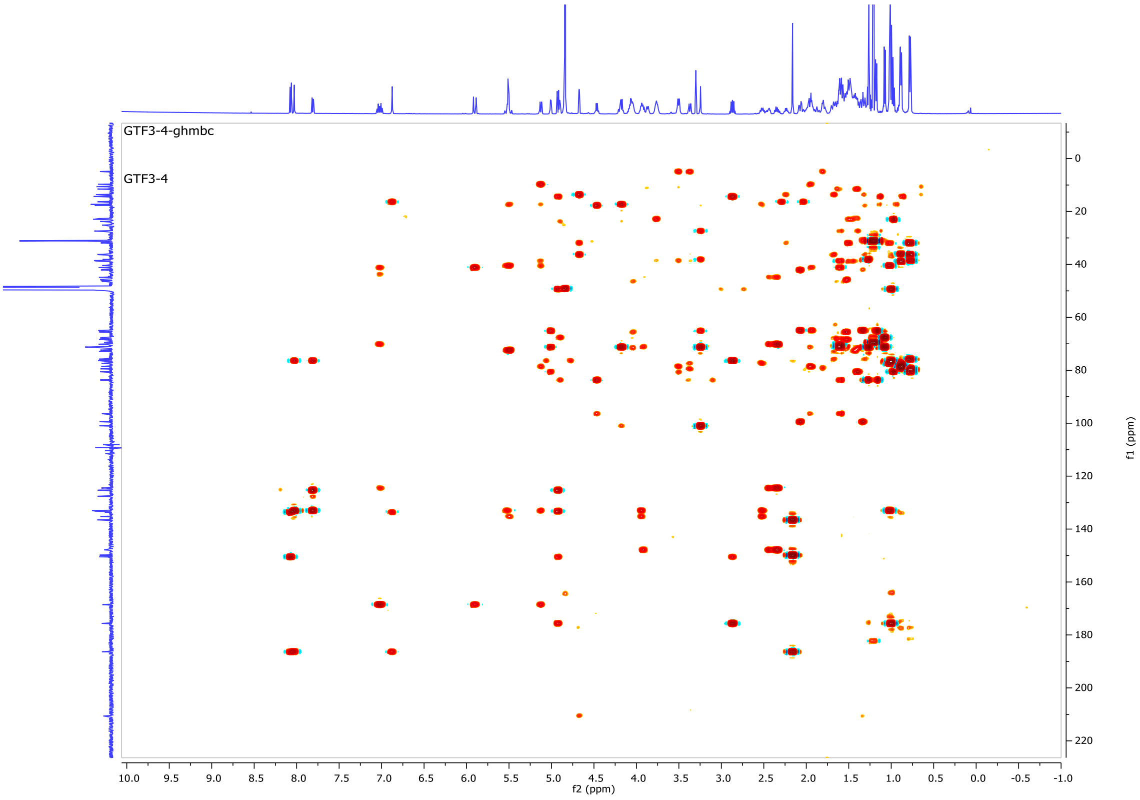

gHMBC spectrum of **13** (CD_3OD, 500 MHz for ^1H and 125 MHz for ^{13}C)

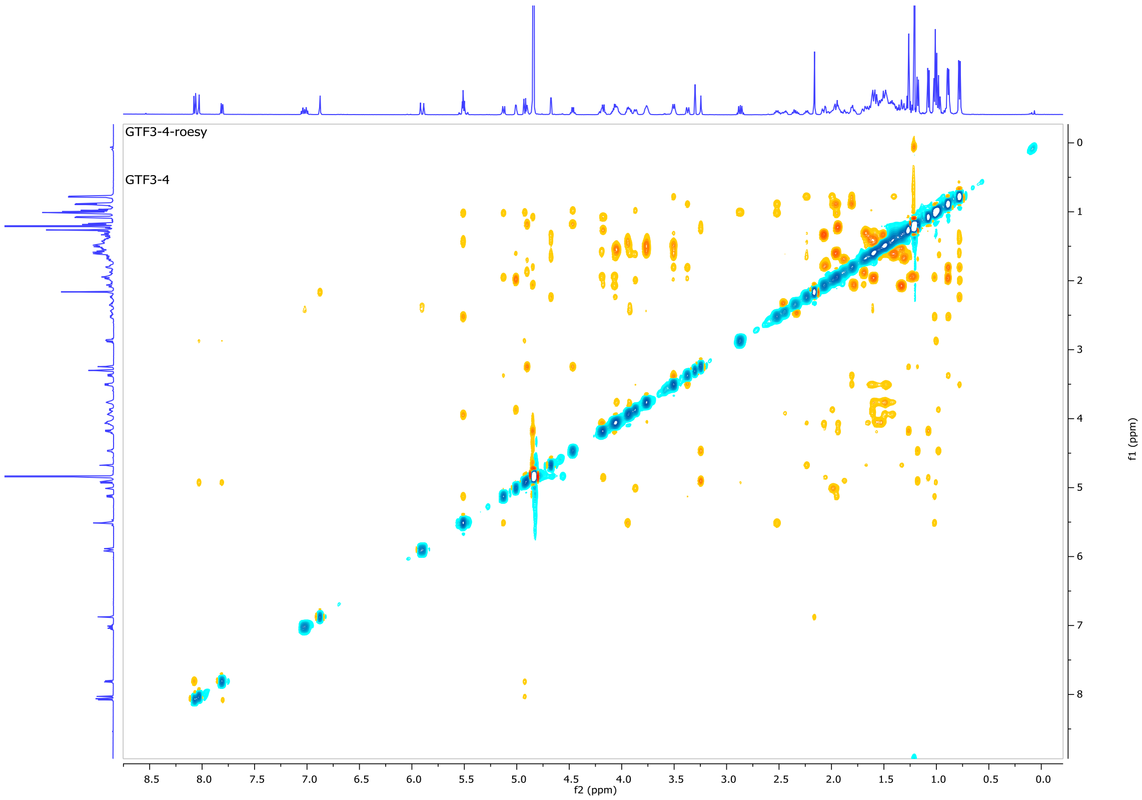

ROESY spectrum of **13** (CD_3OD, 500 MHz for ^1H and 125 MHz for ^{13}C)

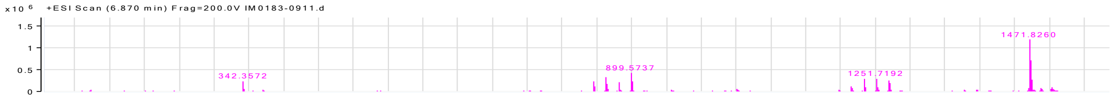

MS spectrum of **13**

Table S1. ^{1}H and ^{13}C NMR (500/125 MHz) data of Compounds **3** and **4** (CD_3OD).

Nº	**3** ^{1}H, mult, *J* = Hz	**3** ^{13}C	**4** ^{1}H, mult, *J* = Hz	Nº	**3** ^{1}H, mult, *J* = Hz	**3** ^{13}C	**4** ^{1}H, mult, *J* = Hz
**1**	-	168.6	-	**42**	1.62, m; 1.55, m	23.0	1.62, m; 1.40, m
**2**	5.91, d, 15.6	124.5	5.91, d, 15.6	**43**	0.97, t, 7.2	11.5	0.98, t, 7.3
**3**	7.02, ddd, 15.2, 7.2, 7.2	147.9	7.02, ddd, 15.1, 7.2, 7.2	**44**	0.82, d, 6.8	13.9	0.78, d, 7.0
**4**	2.45,m; 2.36, m	41.2	2.45,m; 2.36, m	**45**	1.02, d, 6.6	17.3	1.02, d, 6.8
**5**	3.93, m	70.2	3.93, m	**46**	0.88, d, 6.9	9.7	0.88, d, 6.9
**6**	1.61, m	44.9	1.61, m	**47**	0.89, d, 7.0	5.0	0.89, d, 6.9
**7**	3.78, m	71.1	3.77, m	**48**	0.77, d, 7.0	10.4	0.77, d, 7.2
**8**	1.51, m; 1.38, m	38.7	1.52, m; 1.37, m	**1'**	4.87, d, 3.8	95.6	4.99, d, 3.7
**9**	1.62, m; 1.40, m	23.0	1.62, m; 1.40, m	**2'**	1.99, m; 1.39	25.9	1.97, m; 1.40, m
**10**	1.51, m; 1.38, m	38.6	1.51, m; 1.38, m	**3'**	2.02, m; 1.98, m	25.8	2.01, m; 1.98, m
**11**	3.52, m	72.6	3.51, d, 9.1	**4'**	3.56, m	77.6	3.56, s
**12**	1.51, m; 1.38, m	36.2	1.51, m; 1.38, m	**5'**	4.08, m	67.9	4.08, m
**13**	1.66, m; 1.30, m	31.5	1.67, m; 1.31, m	**6'**	1.12, d, 6.6	17.4	1.12, d, 6.5
**14**	2.26, qd, 6.8, 2.4	36.3	2.24, qd, 7.0, 2.7	**1''**	4.60, m	104.8	4.67, d, 2.8
**15**	4.61, d, 2.7	75.6	4.68, d, 2.8	**2''**	1.97, m; 1.66, m	31.5	1.65, m; 1.69, m
**16**	-	210.6	-	**3''**	2.10, m; 1.57, m	28.7	2.06, m; 1.78, m
**17**	-	99.4	-	**4''**	4.46, m	74.6	4.45, m
**18**	3.50, m	75.4	2.07, m; 1.34, m	**5''**	3.61, m	74.3	3.61, m
**19**	3.86, m	69.7	4.07, m	**6''**	1.21, d, 6.7	18.7	1.22, d, 6.6
**20**	1.97, m; 1.40, m	41.1	1.94, m ; 1.22, m	**49**	-	175.3	-
**21**	4.23, dd, 11.5, 9.7	67.5	4.19, dd, 10.7, 10.7	**50**	2.89, dq, 9.7, 7.1	48.4	2.88, dq, 9.9, 7.4
**22**	1.63, m	46.4	1.63, m	**51**	4.73, d, 9.8	84.1	4.7, d, 9.9
**23**	4.00, m	65.7	4.06, m	**52**	-	148.5	-
**24**	1.57, m	46.0	1.57, m	**53**	8.01, d, 1.8	125.9	8.01, d, 1.7
**25**	4.02, m	68.4	4.02, m	**54**	-	133.5	-
**26**	1.53, m	45.6	1.53, m	**55**	-	186.2	-
**27**	3.76, m	71.2	3.75, m	**56**	6.89, q, 1.6	136.5	6.89, q, 1.5
**28**	1.57, m; 1.44, m	38.6	1.57, m; 1.44, m	**57**	-	150.0	-
**29**	1.51, m	22.7	1.51, m	**58**	-	186.2	-
**30**	1.51, m; 1.38, m	38.7	1.51, m; 1.38, m	**59**	-	133.1	-
**31**	3.94, m	72.5	3.93, m	**60**	8.09, d, 8.0	127.6	8.09, d, 8.2
**32**	5.51, dd, 15.4, 4.5	135.2	5.52, dd, 15.5, 4.5	**61**	7.82, dd, 8.1, 1.8	133.9	7.82, dd, 8.2, 1.2
**33**	5.49, dd, 15.4, 7.0	133.1	5.49, 15.5, 7.0	**62**	0.87, d, 7.1	14.3	0.92, d, 7.2
**34**	2.51, m	40.5	2.52, m	**63**	2.17, d, 1.5	16.3	2.16, d, 1.6
**35**	5.14, d, 9.5	77.4	5.13, dd, 9.5, 1.8	**1'''**	4.96, m	101.3	
**36**	1.98, m	38.7	1.99, m	**2'''**	2.00, m; 1.98, m	25.8	
**37**	3.37, dd, 9.4, 2.3	78.5	3.37, dd, 8.7	**3'''**	1.60, m; 1.45, m	25.6	
**38**	1.81, m	36.1	1.81, m	**4'''**	3.31, m	67.6	
**39**	3.51, m	79.5	3.50, m	**5'''**	3.13, q, 6.9	68.3	
**40**	2.00, m	38.8	2.01, m	**6'''**	0.45, d, 6.6	16.8	0.45, d, 6.6
**41**	3.92, m	78.9	3.82, ddd, 9.2, 3.2, 3.2				

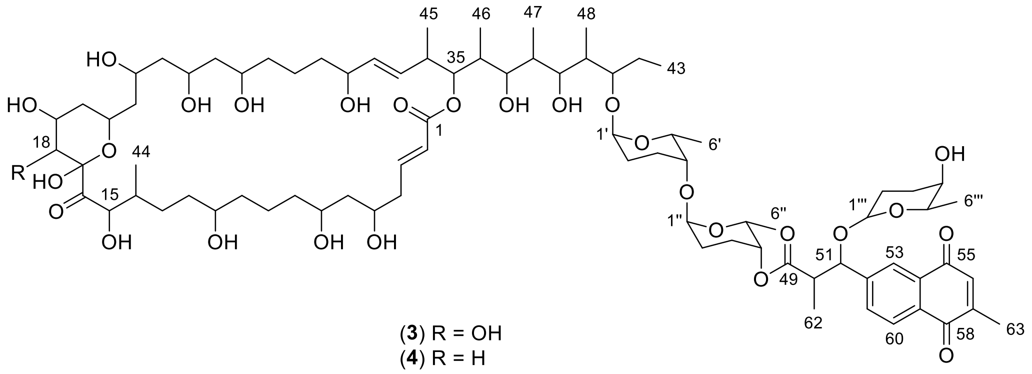
45
46
47
48
35
43
HO
OH
OH
OH
HO
18
R
HO
O
O
1
1'
O
6'
44
15
OH
OH
OH
OH
O
1''
6''
O
49
51
53
55
1'''
6'''
62
60
58
63

(**3**) R = OH
(**4**) R = H

Table S2. ^{1}H and ^{13}C NMR (500/125 MHz) data of Compounds **5** and **13** (CD_3OD).

Nº	**5**<br>^{1}H, mult, J = Hz	**13**<br>^{1}H, mult, J = Hz	**13**<br>^{13}C	Nº	**1**<br>^{1}H, mult, J = Hz	**13**<br>^{1}H, mult, J = Hz	**13**<br>^{13}C
**1**		-	168.5	**42**	1.61, m; 1.39, m	1.61, m; 1.39, m	23.0
**2**	5.90, d, 15.6	5.90, d, 15.6	124.5	**43**	0.99, t, 7.3	0.99, t, 7.3	11.8
**3**	7.02, m	7.02, m	147.9	**44**	0.78, d, 6.8	0.78, d, 6.8	13.6
**4**	2.45,m; 2.34, m	2.45,m; 2.34, m	41.2	**45**	1.02, d, 6.6	1.02, d, 6.6	17.3
**5**	3.92, m	3.92, m	70.2	**46**	0.90, d, 6.9	0.90, d, 6.9	9.7
**6**	1.61, m	1.61, m	44.9	**47**	0.90, d, 7.4	0.90, d, 7.4	4.6
**7**	3.76, m	3.76, m	71.3	**48**	0.79, d, 6.9	0.79, d, 6.9	10.6
**8**	1.38, m; 1.51, m	1.38, m; 1.51, m	38.7	**1’**	5.01, d, 3.7	5.01, d, 3.7	96.4
**9**	1.61, m; 1.39, m	1.61, m; 1.39, m	23.0	**2’**	1.95, dd, 14.0, 3.5; 1.60, d, 13.5	1.95, dd, 14.0, 3.5; 1.60, d, 13.5	38.1
**10**	1.51, m; 1.38, m	1.51, m; 1.38, m	38.6	**3’**	-	-	71.5
**11**	3.50, m	3.50, m	72.9	**4’**	3.25, br s	3.25, br s	83.7
**12**	1.51, m; 1.38, m	1.51, m; 1.38, m	36.2	**5’**	4.47. q, 7.0	4.47. q, 7.0	65.2
**13**	1.67, m; 1.31, m	1.67, m; 1.31, m	31.9	**6’**	1.18, d, 6.5	1.18, d, 6.5	17.7
**14**	2.24, dddd, 7.0, 7.0, 7.0, 2.8	2.24, dddd, 7.0, 7.0, 7.0, 2.8	36.3	**7’**	1.26, s	1.26, s	27.4
**15**	4.67, d, 2.8	4.67, d, 2.8	75.8	**1’’**	4.93, dd, 4.4, 4.4	4.90, d, 3.3	101.0
**16**	-	-	210.6	**2’’**	2.07, ddd, 14.4, 4.3, 4.2; 1.95, m	1.87, m; 1.69, m	25.1
**17**	-	-	99.5	**3’’**	3.86, m	2.06, m; 1.78, m	23.8
**18**	2.07, dd, 13.3, 3.8 ; 1.33, dd, 12.8, 11.2	2.07, dd, 13.3, 3.8 ; 1.33, dd, 12.8, 11.2	41.2	**4’’**	4.74, dd, 4.5, 2.3	4.85, m	71.3
**19**	4.06, m	4.06, m	64.9	**5’’**	4.44, m	4.18, dq, 6.3, 1.4	67.9
**20**	1.94, m ; 1.22, m	1.94, m ; 1.22, m	42.1	**6’’**	1.11, d, 6.7	1.08, d, 6.6	17.3
**21**	4.18, m	4.18, m	67.6	**49**	-	-	175.6
**22**	1.63, m	1.63, m	46.4	**50**	2.87, dq, 9.8, 7.1	2.87, dq, 8.4, 7.1	49.0
**23**	4.07, m	4.07, m	65.5	**51**	4.78, d, 10.0	4.92, d, 8.2	76.4
**24**	1.57, m	1.57, m	46.0	**52**	-	-	150.5
**25**	4.04, m	4.04, m	68.3	**53**	8.03, d, 1.7	8.03, d, 1.4	125.3
**26**	1.53, m	1.53, m	45.6	**54**	-	-	133.6
**27**	3.76, m	3.76, m	71.1	**55**	-	-	186.4
**28**	1.57, m; 1.44, m	1.57, m; 1.44, m	38.6	**56**	6.88, q, 1.6	6.88, q, 1.5	136.5
**29**	1.51, m	1.51, m	22.7	**57**	-	-	150.0
**30**	1.51, m; 1.38, m	1.51, m; 1.38, m	38.6	**58**	-	-	186.3
**31**	3.94, m	3.94, m	72.4	**59**	-	-	133.0
**32**	5.53, dd, 15.5, 4.5	5.53, dd, 15.5, 4.5	135.2	**60**	8.07, d, 8.0	8.07, d, 8.0	127.6
**33**	5.49, 15.5, 7.0	5.49, 15.5, 7.0	133.0	**61**	7.81, dd, 8.0, 1.7	7.81, dd, 8.1, 1.8	133.3
**34**	2.52, ddd, 9.5, 6.7, 6.7	2.52, ddd, 9.5, 6.7, 6.7	40.4	**62**	0.89, d, 7.1	0.92, d, 7.2	14.3
**35**	5.13, dd, 9.7, 1.7	5.13, dd, 9.7, 1.7	77.4	**63**	2.16, d, 1.5	2.16, d, 1.6	16.3
**36**	1.99, m	1.99, m	38.8				
**37**	3.37, dd, 9.6, 2.0	3.37, dd, 9.6, 2.0	78.6				
**38**	1.80, m	1.80, m	36.1				
**39**	3.50, m	3.50, m	79.5				
**40**	1.99, m	1.99, m	38.8				
**41**	3.87, ddd, 9.5, 3.5, 3.5	3.87, ddd, 9.5, 3.5, 3.5	80.6				

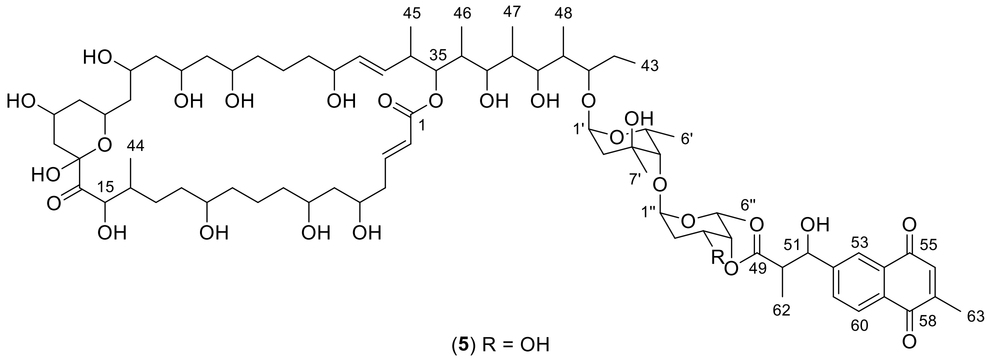

45
46
47
48
35
43
HO
HO
OH
OH
OH
OH
OH
O
O
1
OH
O
1'
6'
7'
O
HO
44
15
O
OH
OH
OH
OH
1''
O
6''
O
OH
51
53
55
R
O
49
62
60
58
63
O
O

(**5**) R = OH
(**13**) R = H

Table S3. ^{1}H NMR (500/125 MHz) data of Compounds **6** and **7** (CD_3OD).

N°	**6**<br>^{1}H, mult, *J* = Hz	**7**<br>^{1}H, mult, *J* = Hz
**1**	-	-
**2**	2.78, m	-
**3**	4.92, d, 8.3	8.62, dd, 1.6
**4**	0.95, d, 7.1	-
**5**	-	6.95, q, 1.5
**6**	8.03, d, 1.7	-
**7**	-	-
**8**	-	
**9**	6.88, q, 1.4	
**10**	-	8.17, d, 8.1
**11**	-	8.31, dd, 8.1, 1.7
**12**	-	2.20, d, 1.6
**13**	8.07, d, 8.0	
**14**	7.79, dd, 8.0, 1.8	
**15**	0.95, d, 7.1	

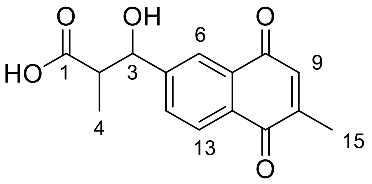

(**6**)

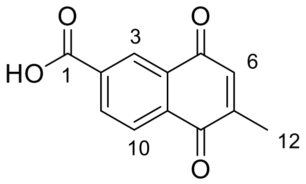

(**7**)

Table S4. ^{1}H and ^{13}C NMR (500/125 MHz) data of Compound **10** (CD_3OD).

Nº	**10**<br>^{1}H, mult, *J* = Hz	**10**<br>^{13}C
**1**		168.5
**2**	5.90, dd, 15.6, 1.5	124.5
**3**	7.02, ddd, 15.5, 7.3, 7.3	147.8
**4**	2.44,m; 2.34, m	41.1
**5**	3.91, m	70.1
**6**	1.61, m	44.9
**7**	3.76, m	71.1
**8**	1.38, m; 1.51, m	38.8
**9**	1.50, m	23.0
**10**	1.38, m; 1.51, m	38.5
**11**	3.50, m	72.9
**12**	1.57, m; 1.43, m	36.3
**13**	1.67, m; 1.30, m	31.9
**14**	2.24, qd, 7.0, 2.8	36.2
**15**	4.67, d, 2.8	75.8
**16**	-	210.6
**17**	-	99.5
**18**	2.07, ddd, 13.3, 4.7, 1.6<br>1.33, dd, 12.2, 11.9	41.2
**19**	4.08, m	64.9
**20**	1.93, m ; 1.23, m	42.1
**21**	4.18, m	67.8
**22**	1.64, m; 1.57, m	45.6
**23**	4.06, m	65.5
**24**	1.57, m	46.0
**25**	4.04, m	68.3
**26**	1.53, m	46.0
**27**	3.76, m	71.5
**28**	1.57, m; 1.44, m	38.7
**29**	1.50, m; 1.34, m	22.7
**30**	1..51, m; 1.38, m	38.6
**31**	3.94, m	72.4
**32**	5.53, dd, 15.5, 4.5	135.2
**33**	5.49, 15.5, 7.0	133.0
**34**	2.51, ddd, 9.5, 6.9, 6.9	40.5
**35**	5.14, dd, 9.6, 1.8	77.3
**36**	1.94, m	38.7
**37**	3.39, dd, 9.6, 2.0	78.7
**38**	1.83, m	36.0
**39**	3.58, dd, 9.4, 2.0	81.5
**40**	1.76, m	42.5
**41**	3.66, ddd, 8.8, 5.9, 2.5	76.3
**42**	1.57, m; 1.32, m	26.3
**43**	0.96, t, 7.4	10.5
**44**	0.78, d, 6.9	13.6
**45**	1.02, d, 6.8	17.3
**46**	0.88, d, 6.9	9.7
**47**	0.89, d, 6.9	4.6
**48**	0.76, d, 7.0	11.8

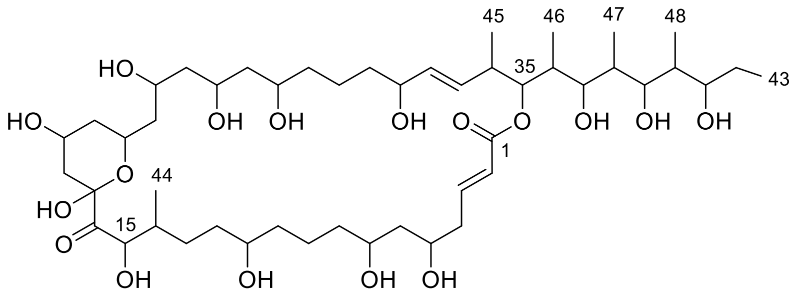

(**10**)

Table S5. ^{1}H and ^{13}C NMR (500/125 MHz) data of Compounds **11** and **12** (CD_3OD).

Nº	**11** ^{1}H, mult, *J* = Hz	**11** ^{13}C	**12** ^{1}H, mult, *J* = Hz	**12** ^{13}C	Nº	**11** ^{1}H, mult, *J* = Hz	**11** ^{13}C	**12** ^{1}H, mult, *J* = Hz	**2** ^{13}C
**1**	-	168.6	-	168.6	**42**	1.62, m; 1.39, m	22.8	1.62, m; 1.40, m	23.0
**2**	5.91, ddd, 15.7, 1.5, 1.5	124.5	5.91, ddd, 15.7, 1.5, 1.5	124.5	**43**	0.98, t, 7.3	11.6	0.9, t, 7.4	11.7
**3**	7.02, ddd, 15.0, 7.3, 7.3	147.9	7.02, ddd, 15.0, 7.3, 7.3	147.9	**44**	0.78, d, 6.9	13.6	0.82, d, 6.8	13.9
**4**	2.45,m; 2.34, m	41.1	2.45,m; 2.34, m	41.1	**45**	1.01, d, 6.8	17.3	1.01, d, 6.8	17.1
**5**	3.92, m	70.1	3.92, m	70.1	**46**	0.88, d, 6.9	9.7	0.88, d, 6.9	9.7
**6**	1.61, m	44.9	1.61, m	44.9	**47**	0.89, d, 7.0	5.0	0.89, d, 6.9	5.0
**7**	3.74, m	71.4	3.76, m	71.0	**48**	0.77, d, 6.9	10.7	0.77, d, 6.9	10.6
**8**	1.51, m; 1.38, m	38.7	1.51, m; 1.38, m	38.7	**1'**	4.99, d, 3.8	96.5	4.99, d, 3.7	96.4
**9**	1.62, m; 1.40, m	23.0	1.62, m; 1.40, m	23.0	**2'**	1.98, m; 1.53, dd, 14.0, 3.0	37.0	1.97, m; 1.63, m	37.0
**10**	1.51, m; 1.38, m	38.6	1.51, m; 1.38, m	38.6	**3'**	-	71.7	-	71.7
**11**	3.52, m	72.6	3.52, m	72.6	**4'**	3.03, br s	75.3	3.03, br s	75.3
**12**	1.51, m; 1.38, m	36.2	1.51, m; 1.38, m	36.2	**5'**	4.41. br q, 6.6	64.5	4.41. br q, 6.8	65.2
**13**	1.66, m; 1.30, m	31.5	1.67, m; 1.31, m	31.5	**6'**	1.18, d, 7.0	17.1	1.18, d, 6.8	17.7
**14**	2.23, qd, 6.9, 2.8	36.3	2.20, qd, 6.9, 3.4	36.2	**7'**	1.19, s	26.8	1.19, s	26.8
**15**	4.67, d, 2.8	75.8	4.60, d, 3.4	75.5					
**16**	-	210.6	-	208.7					
**17**	-	99.4	-	99.7					
**18**	2.07, ddd, 12.4, 4.4, 1.6; 1.34, m	41.1	3.50, m	75.4					
**19**	4.07, m	65.0	3.87, m	69.7					
**20**	1.94, m; 1.22, m	42.1	1.97, m ; 1.43, m	41.0					
**21**	4.19, dd, 11.9,9.3	67.8	4.23, dd, 11.9, 9.1	67.5					
**22**	1.63, m	46.4	1.63, m	46.3					
**23**	4.06, m	65.5	4.00, m	65.7					
**24**	1.57, m	46.0	1.57, m	45.8					
**25**	4.04, m	68.3	4.02, m	68.4					
**26**	1.53, m	45.6	1.53, m	45.1					
**27**	3.76, m	71.5	3.74, m	71.4					
**28**	1.57, m; 1.44, m	38.6	1.57, m; 1.44, m	38.6					
**29**	1.51, m	22.7	1.51, m	22.8					
**30**	1.51, m; 1.38, m	38.6	1.51, m; 1.38, m	38.7					
**31**	3.93, m	72.4	3.94, m	72.5					
**32**	5.51, dd, 15.4, 4.5	135.2	5.52, dd, 15.5, 4.5	135.2					
**33**	5.49, dd, 15.4, 7.0	133.0	5.49, dd, 15.5, 7.0	133.1					
**34**	2.52,ddd, 9.7, 6.9, 6.9	40.5	2.51, m	40.5					
**35**	5.13, dd, 9.6, 1.8	77.4	5.14, dd, 9.5, 1.8	77.4					
**36**	2.00, m	38.7	1.99, m	38.8					
**37**	3.37, dddd, 9.4, 1.8, 0.6, 0.6	78.5	3.37, dd, 9.6, 2.1	78.5					
**38**	1.81, m	36.1	1.81, m	36.2					
**39**	3.50, m	79.5	3.50, m	79.5					
**40**	2.00, m	38.8	2.01, m	38.8					
**41**	3.87, ddd, 9.6, 3.1, 3.1	80.3	3.87, m	80.3					

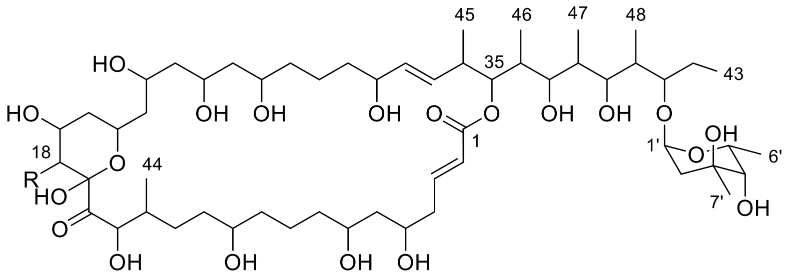
45
46
47
48
HO
35
43
HO
OH
OH
OH
O
O
OH
OH
1
O
OH
18
O
1'
O
6'
44
R
HO
7'
OH
O
OH
OH
OH
OH

(**11**) R = H
(**12**) R = OH
