# Supplementary material for: Elucidation of the glycosylation steps during biosynthesis of antitumor macrolides PM100117 and PM100118 and engineering for novel derivatives
Source: Microb Cell Fact. 2016 Nov 9;15:187. doi: 10.1186/s12934-016-0591-7 (PMC5103430; doi:10.1186/s12934-016-0591-7)
Supplement: Supplementary file 3 — Additional file 3. Fig. S2. Effect of gonR2 over-expression on PM100117/18 biosynthesis. Fig. S3. Effects of gonG4 over-expression on S. caniferus GUA-06-05-006A and ΔgonG2. Methods: Feeding experiment. Fig. S4. Heterologous expression of gonG4 in Streptomyces albus J1074 and feeding with PM100118. [file 12934_2016_591_MOESM3_ESM.pdf]

Fig. S2. Effect of *gonR2* over-expression on PM100117/18 biosynthesis. Strains GUA-pSETH (control) and OE*gonR2* (pC*gonR2*) were analyzed for PM100117 (**1**) and PM100118 (**2**) production by UPLC.

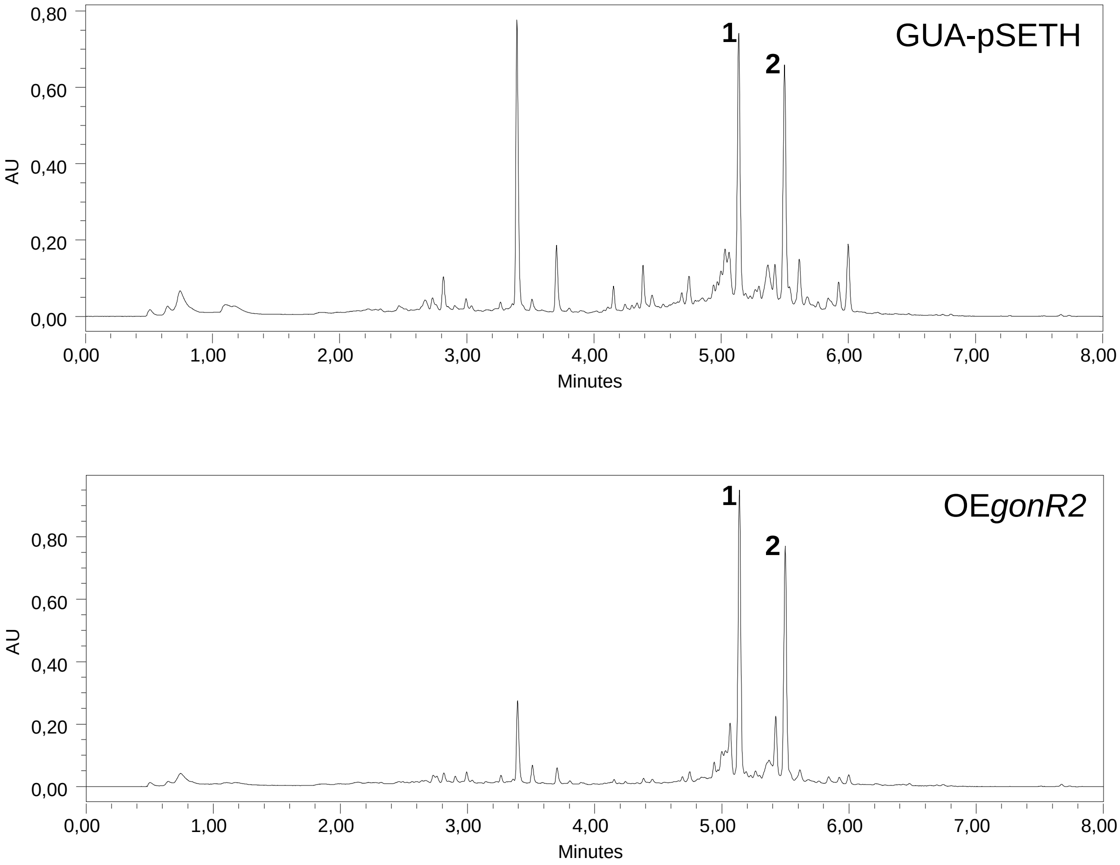

Fig. S3. Effects of *gonG4* over-expression on *S. caniferus* GUA-06-05-006A and Δ*gonG2*. A) UPLC analysis of PM100117/18 production in strains GUA-pSETH and OE*gonG4*. B) UPLC analysis of the effect of *gonG4* over-expression in strain Δ*gonG2*.

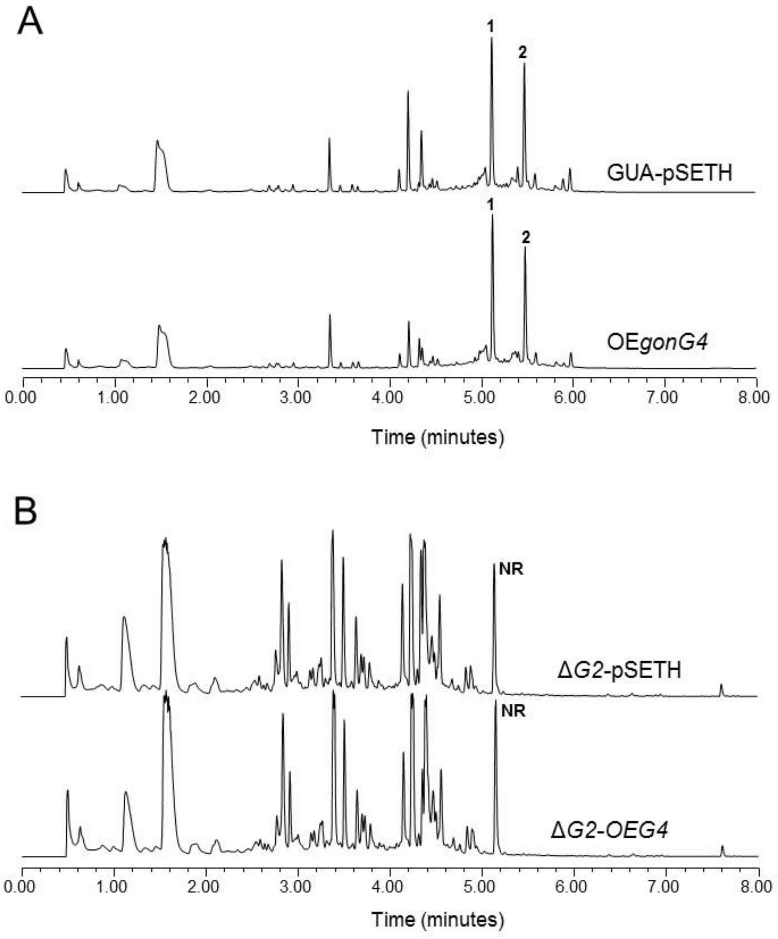

Methods: Feeding experiment

Plamids pSETH and pC-*gonG4* were transfer into *Streptomyces albus* J1074 by intergeneric conjugation yielding strains SA-pSETH and SA-OE*G4*, which were cultivated in TSB medium for 24 h at 30ºC and 200 rpm. Each one of these seed cultures was used to inoculate (2% v/v) two 250-ml flasks containing 25 ml of R5A medium (sucrose, 100 g/liter; K_2SO_4, 0.25 g/liter; $MgCl_2 \cdot 6H_2O$, 10.12 g/liter; glucose,

10 g/liter; Casamino Acids, 0.1 g/liter; yeast extract, 5 g/liter; and MOPS, 21 g/liter). After 24 h of cultivation a pulse feeding with 100% methanol (control) or 200ug of PM100118 in methanol was started. The culture was then left in agitation for additional 8 h before the first sample extraction (3 ml), which was continued by additional sample collection 24, 48 and 72 h after the pulse. Samples were extracted with 3 ml of ethyl acetate as described in methods and analyzed by HPLC/MS to detect the appearance of PM100118 derivatives carrying glucose subunits.

Fig. S4. Heterologous expression of *gonG4* in *Streptomyces albus* J1074 and feeding with PM100118. Strains SA-pSETH and SA-OE*G4* were fed with PM100118 or methanol, samples were taken at A) 8 h, B) 24 h, C) 48 h and 72 h, and analyzed by HPLC/MS. The peak labeled with an *asterisk* corresponds to a PM100118 (2) shunt product that was co-purified with the respective parental compound. *PD* is a differential peak corresponding to the *S. albus* J1074 natural product paulomycin D.

A

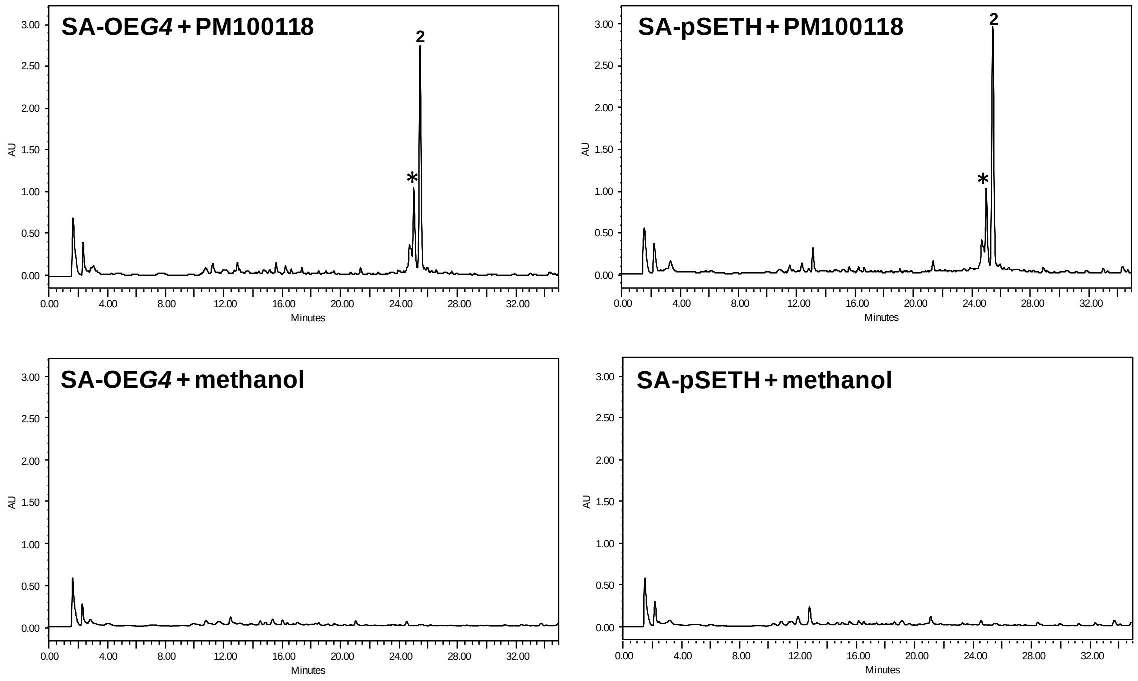

B

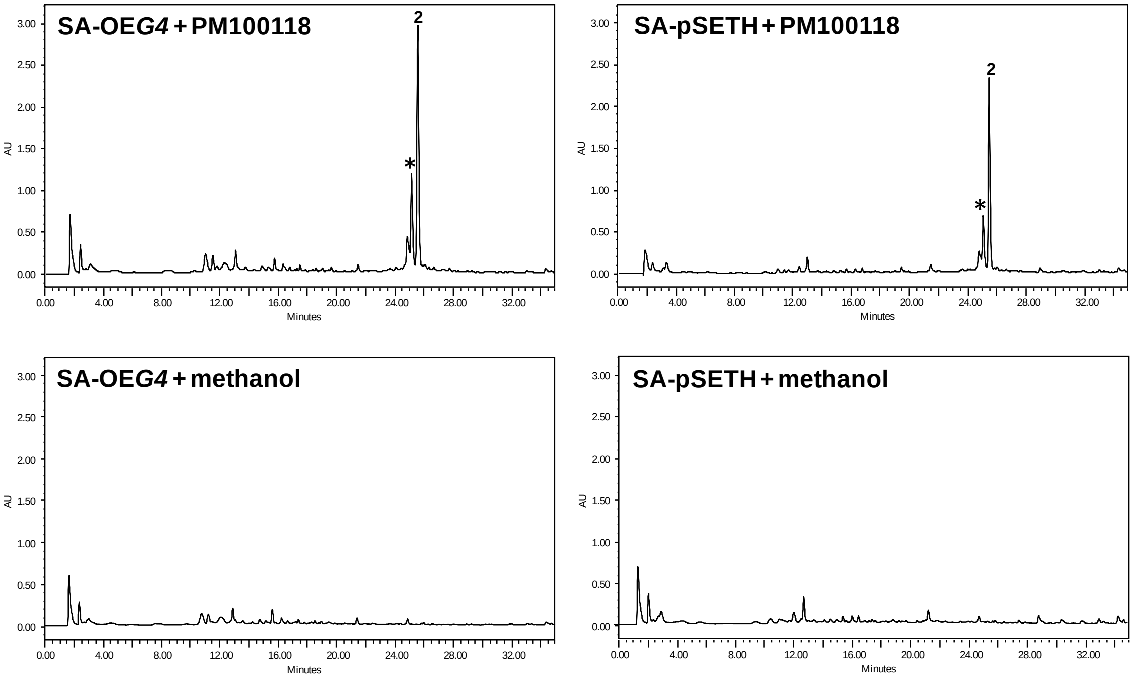
SA-OEG4 + PM100118
2
*
SA-pSETH + PM100118
2
*
SA-OEG4 + methanol
SA-pSETH + methanol
AU
Minutes
0.00
0.50
1.00
1.50
2.00
2.50
3.00
0.00
4.00
8.00
12.00
16.00
20.00
24.00
28.00
32.00

C

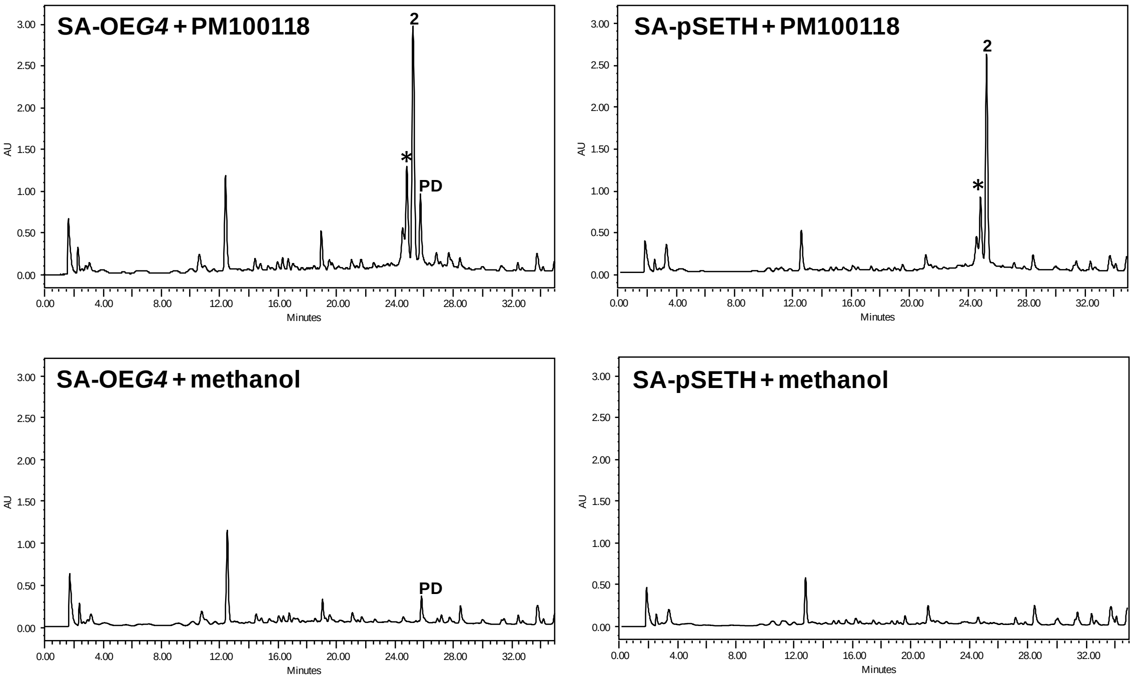
SA-OEG4 + PM100118
2
*
PD
SA-pSETH + PM100118
2
*
SA-OEG4 + methanol
PD
SA-pSETH + methanol
AU
Minutes
0.00
0.50
1.00
1.50
2.00
2.50
3.00
0.00
4.00
8.00
12.00
16.00
20.00
24.00
28.00
32.00

D

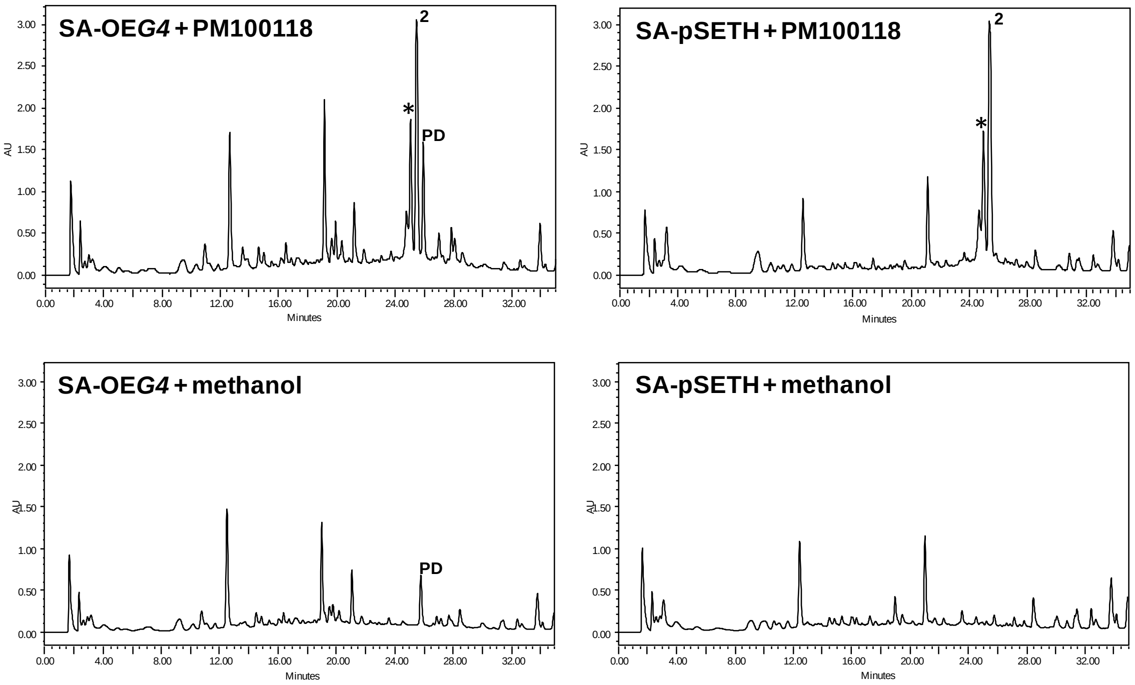
SA-OEG4 + PM100118
SA-pSETH + PM100118
SA-OEG4 + methanol
SA-pSETH + methanol
2
*
PD
AU
Minutes
