# Supplementary material for: Elucidation of the glycosylation steps during biosynthesis of antitumor macrolides PM100117 and PM100118 and engineering for novel derivatives
Source: Microb Cell Fact. 2016 Nov 9;15:187. doi: 10.1186/s12934-016-0591-7 (PMC5103430; doi:10.1186/s12934-016-0591-7)
Supplement: Supplementary file 4 — Additional file 4. Table S6. Primers and plasmids used in this work. Fig. S5. Gene deletion confirmation by PCR in mutant strains ∆gonG1, ∆gonG2, ∆gonG3, ∆gonG4, ∆gonR1, ∆gonE, ∆gonCM and ∆gonD3. [file 12934_2016_591_MOESM4_ESM.pdf]

Table S6. Primers and plasmids used in this work

Primer	Sequence (5'-3')	PCR product and purpose	Plasmid
		**Gene deletion**	
**NsiI-***CM*	TATA**ATGCAT**AGTTGTCCCCGTACATCAGC	2,167-bp upstream flanking region for *gonCM* deletion	pD*gonCM*
**SpeI-***CM*	TATA**ACTAGT**TAGGTCTCGACGGCCTTGTA		
**EcoRV-***CM*	TATA**GATATC**CCTTTCTTGGTCGTGAAGGT	2,099-bp downstream flanking region for *gonCM* deletion	
**NdeI-***CM*	TATA**CATATG**CCCGAACTCTCCATCGTCT		
**NsiI-***D3*	TATA**ATGCAT**GACGTGCTCCTGGTGGTACT	2,072-bp upstream flanking region for *gonD3*deletion	pD*gonD3*
**SpeI-***D3*	TATA**ACTAGT**GAGGACACCGTTCGTGGTT		
**EcoRV-***D3*	TATA**GATATC**CACGAGTACGCCCCACAG	2,106-bp downstream flanking region for *gonD3*deletion	
**BamHI-***D3*	TATA**GGATCC**TCACCGAGTTCGTTCTCTCC		
**NsiI-***E*	TATA**ATGCAT**TCCTGCAATGGTGAGAAGAA	2,270-bp upstream flanking region for *gonE* deletion	pD*gonE*
**SpeI-***E*	TATA**ACTAGT**CGCTGTCTGACGATGGAGTA		
**EcoRV-***E*	TATA**GATATC**TCACACCATTCTGCTTCTCG	2,255-bp downstream flanking region for *gonE* deletion	
**BamHI-***E*	TATA**GGATCC**CTCCGTGACTGATGAGGACA		
**SpeI-***R1*	TATA**ACTAGT**GTGACCTACCTTCCGCAGAG	2,147-bp upstream flanking region for *gonR1* deletion	pD*gonR1*
**NsiI-***R1*	TATA**ATGCAT**GTCAGGTCCATCGGGAAGA		
**BamHI-***R1*	TATA**GGATCC**CCGCAGCATCGACAGTTC	2,021-bp downstream flanking region for *gonR1* deletion	
**EcoRV-***R1*	TATA**GATATC**TACATCTTCTCGCACGTTGG		
**NsiI-***R2*	TATA**ATGCAT**ACTGCACGTTGGTGTAGTGC	1,981-bp upstream flanking region for *gonR2* deletion	pD*gonR2*
**SpeI-***R2*	TATA**ACTAGT**GCTGATGTACGGGGACAACT		
**EcoRV-***R2*	TATA**GATATC**ACAGCCTTTAGGCGTGTTGT	2,277-bp downstream flanking region for *gonR2* deletion	
**BamHI-** *R2*	TATA**GGATCC**GGGTGAGGTCCACAGCAG		
**SpeI-***G1*	TATA**ACTAGT**CTAACCGCCGTCGTACATCT	2,219-bp upstream flanking region for *gonG1* deletion	pD*gonG1*
**NsiI-***G1*	TATA**ATGCAT**GTCTCATCCTGTGGTCTGTGG		
**BamHI-***G1*	TATA**GGATCC**GGGATCTACACCCACCACAG	2,016-bp downstream flanking region for *gonG1* deletion	
**EcoRV-***G1*	TATA**GATATC**AGGAGATGGGTGACGAACAG		

Table S1. Primers and plasmids used in this work (continued)

**Primer**	**Sequence (5'-3')**	**PCR product and purpose**	**Plasmid**
		**Gene deletion**	
**SpeI**-*G2*	TATA**<u>ACTAGT</u>**TGTCGACCGTTCCATACTCA	2,033-bp upstream flanking region for *gonG2* deletion	pD*gonG2*
**NsiI**-*G2*	TATA**<u>ATGCAT</u>**TGTAGTGGTGGTCGACGAAG		
**BglII**-*G2*	TATA**<u>AGATCT</u>**GGCCTGTTGGAGCAGTGG	2,079-bp downstream flanking region for *gonG2* deletion	
**EcoRV**-*G2*	TATA**<u>GATATC</u>**AGAGAGCCCTCCCTCATCTC		
**XbaI**-*G3*	TATA**<u>TCTAGA</u>**CCACACGGAATGAGGAGATT	2,110-bp upstream flanking region for *gonG3* deletion	pD*gonG3*
**NsiI**-*G3*	TATA**<u>ATGCAT</u>**ACTGCACCATGACCAGTGAC		
**NdeI**-*G3*	TATA**<u>CATATG</u>**CGGCGAACATACTGGAATCT	2,385-bp downstream flanking region for *gonG3* deletion	
**EcoRV**-*G3*	TATA**<u>GATATC</u>**ACGAGATCACCTCGCATCTT		
**SpeI**-*G4*	TATA**<u>ACTAGT</u>**GGGCACCTTGAAATAGGACA	2,000-bp upstream flanking region for *gonG4* deletion	pD*gonG4*
**NsiI**-*G4*	TATA**<u>ATGCAT</u>**CATGTGAAGGTATCGGCTCA		
**BamHI**-*G4*	TATA**<u>GGATCC</u>**ACAGCCTTTAGGCGTGTTGT	2,331-bp downstream flanking region for *gonG4* deletion	
**EcoRV**-*G4*	TATA**<u>GATATC</u>**CCTCACAGATGGTCCTCGAC		
		**Mutant complementation (over-expression)**	
**EcoRV**-c*CM*	TATA**<u>GATATC</u>**GCTGTGTCTGCCTCAGTATGG	1,426-bp fragment containing *gonCM* (-109 bp from start codon) for mutant complementation	pC*gonCM*
**BamHI**-c*CM*	TATA**<u>GGATCC</u>**TAAGGTCGCCGGCAGATAC		
**EcoRV**-c*D3*	TATA**<u>GATATC</u>**GGCCTGTTGGAGCAGTGG	1,466-bp fragment containing *gonD3* (-32 bp from start codon) for mutant complementation	pC*gonD3*
**BamHI**-c*D3*	TATA**<u>GGATCC</u>**GGCAGGACACTCACAGGAG		
**EcoRV**-c*E*	TATA**<u>GATATC</u>**GATCGCGAGGACTTCGTTC	875-bp fragment containing *gonE* (-106 bp from start codon) for mutant complementation	pC*gonE*
**BamHI**-c*E*	TATA**<u>GGATCC</u>**CGAAAACGTGAGTGACATGAG		
**EcoRV**-c*R2*	TATA**<u>GATATC</u>**CCTTGCGGATGGGTATCTC	1,083-bp fragment containing *gonR2* (-88 bp from start codon) for over-expression	pC*gonR2*
**BamHI**-c*R2*	TATA**<u>GGATCC</u>**CTCCGTGACTGATGAGGACA		

Table S1. Primers and plasmids used in this work (continued)

**Primer**	**Sequence (5'-3')**	**PCR product and purpose**	**Plasmid**
		**Mutant complementation (over-expression)**	
**BglII**-c*G1*	TATA**AGATCT**CCACAGACCACAGGATGAGA	1,436-bp fragment containing *gonG1* (-100 bp from start codon) for mutant complementation	pC*gonG1*
**EcoRV**-c*G1*	TATA**GATATC**CACAAGAGCCTTCACGGTTT		
**BamHI**-c*G2*	TATA**GGATCC**CGAGTGAAGGTGTCGATGTG	1,357-bp fragment containing *gonG2* (-107 bp from start codon) for mutant complementation	pC*gonG2*
**EcoRV**-c*G2*	TATA**GATATC**TCACCGAGTTCGTTCTCTCC		
**BglII**-c*G3*	TATA**AGATCT**TAGTCATTCAGCACCGTCGT	1,429-bp fragment containing *gonG3* (-92 bp from start codon) for mutant complementation	pC*gonG3*
**EcoRV**-c*G3*	TATA**GATATC**ACCTGACGCCACCCTATTC		
**BamHI**-c*G4*	TATA**GGATCC**GGTGAAAGAGCTTGGAGTGC	1,209-bp fragment containing *gonG4* (-24 bp from start codon) for over-expression	pC*gonG4*
**EcoRV**-c*G4*	TATA**GATATC**CTAGGGCGAACACGACCTCT		
**NcoI**-dHG^{R}	TATA**CCATGG**CATCAGCAAAAGGGGATGAT	1,635-bp fragment containing hygR gene from pDR4	pSETH
**NcoI**-rvHG^{R}	TATA**CCATGG**ATCGAAGAGAAGCAGGACGA		
		**Mutant confirmation**	
cf*CM*	CATCGTGGTGGTCTTACCG	used with ApraII for confirmation of *gonCM* deletion	
cf*D3*	GCCGTATGGCTGTGTTCTC	used with ApraII for confirmation of *gonD3*deletion	
cf*E*	AACTTCACCACATCGCACAG	used with ApraII for confirmation of *gonE* deletion	
cf*R1*	CAAGCCGATCCTCTTCTACG	used with Apra60 for confirmation of *gonR1*deletion	
cf*G1*	GTACGGCACCTCCTGCTG	used with Apra60 for confirmation of *gonG1*deletion	
cf*G2*	ACTACGTCCGTACGCTCCTG	used with Apra60 for confirmation of *gonG2* deletion	

Table S1. Primers and plasmids used in this work (continued)

Primer	Sequence (5'-3')	PCR product and purpose	Plasmid
		**Mutant confirmation**	
cfG3	TGCAGCACCTCAAGAGAGAA	used with Apra60 for confirmation of gonR3deletion	
cf*G4*	GTGAAACCGAGTTGATGCAG	used with Apra60 for confirmation of gonG4deletion	
Apra60	CCAAGGTTGAGAAGCTGACC	reverse primer annealing *aac(3)IV*	
ApraII	CTTCAGGATGGCAAGTTGGT	forward primer annealing *aac(3)IV*	

Mutant confirmation by PCR

Gene deletion in *S. caniferus* GUA-06-05-006A was confirmed by colony PCR by using the procedure described in [1] with the following modifications. Colonies of *S. caniferus* GUA-06-05-006A mutants were suspended in 50 µl of 0.2M TES buffer, pH 7.5, with 1 µl lysozyme (50 mg/ml) and incubated for 40 min at 30ºC. The mix was centrifuged (10000 x g, 2 min) and the pellet thoroughly suspended in 10 µl DMSO. The resulting suspension (2 µl) was used as PCR template with the primer pairs described in Table S6. DNA bands obtained from PCR amplification were analyzed in 0.7% agarose gels. Fig. S5 shows the analysis of two colonies (1 and 2) and a control template (C) consisting in 30 ng of wild type *S. caniferus* GUA-06-05-006A genomic DNA from a pure extract. Band sizes were compared with 0.1 µg of lambda/PstI DNA marker. The expect band size is indicated in red.

Fig. S5. Gene deletion confirmation by PCR in mutant strains Δ*gonG1*, Δ*gonG2*, Δ*gonG3*, Δ*gonG4*, Δ*gonR1*, Δ*gonE*, Δ*gonCM* and Δ*gonD3*.

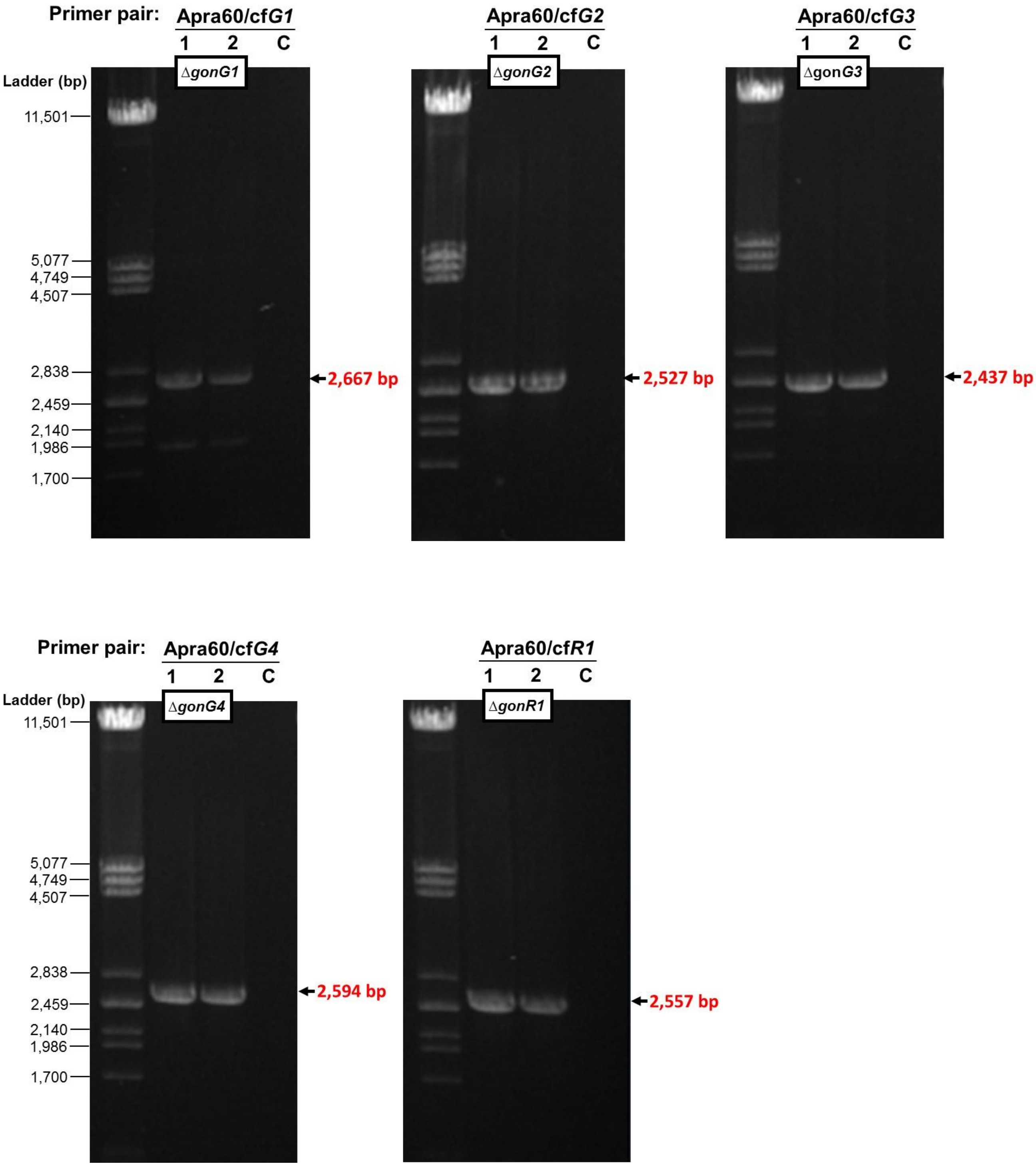

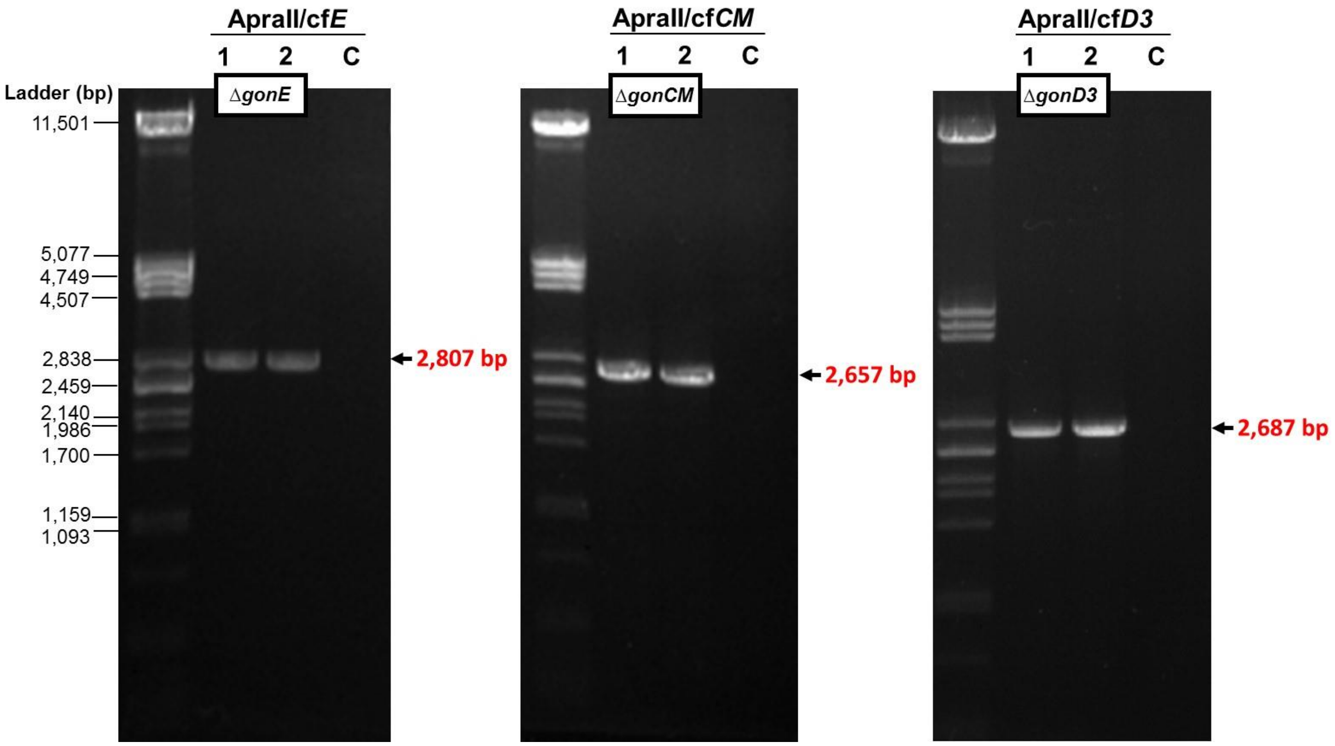

References

[1] Van Dessel W, Van Mellaert L, Geukens N, Anné J: **Improved PCR-based method for the direct screening of Streptomyces transformants.** *J Microbiol Methods* 2003 53:401-3.
