# Supplementary material for: Elucidation of the glycosylation steps during biosynthesis of antitumor macrolides PM100117 and PM100118 and engineering for novel derivatives
Source: Microb Cell Fact. 2016 Nov 9;15:187. doi: 10.1186/s12934-016-0591-7 (PMC5103430; doi:10.1186/s12934-016-0591-7)
Supplement: Supplementary file 5 — Additional file 5. Fig. S6. Genetic complementation of mutant strains ΔgonCM, ΔgonD3, ΔgonE, ΔgonG1, ΔgonG2 and ΔgonG3. [file 12934_2016_591_MOESM5_ESM.pdf]

Figure S6. Genetic complementation of mutant strains Δ*gonCM*, Δ*gonD3*, Δ*gonE*, Δ*gonG1*, Δ*gonG2* and Δ*gonG3*. UPLC analysis of PM100117 (1) and PM100118 (2) production in strains GUA-pSETH, C*gonCM*, C*gonD3*, C*gonE*, C*gonG1*, C*gonG2* and C*gonG3*.

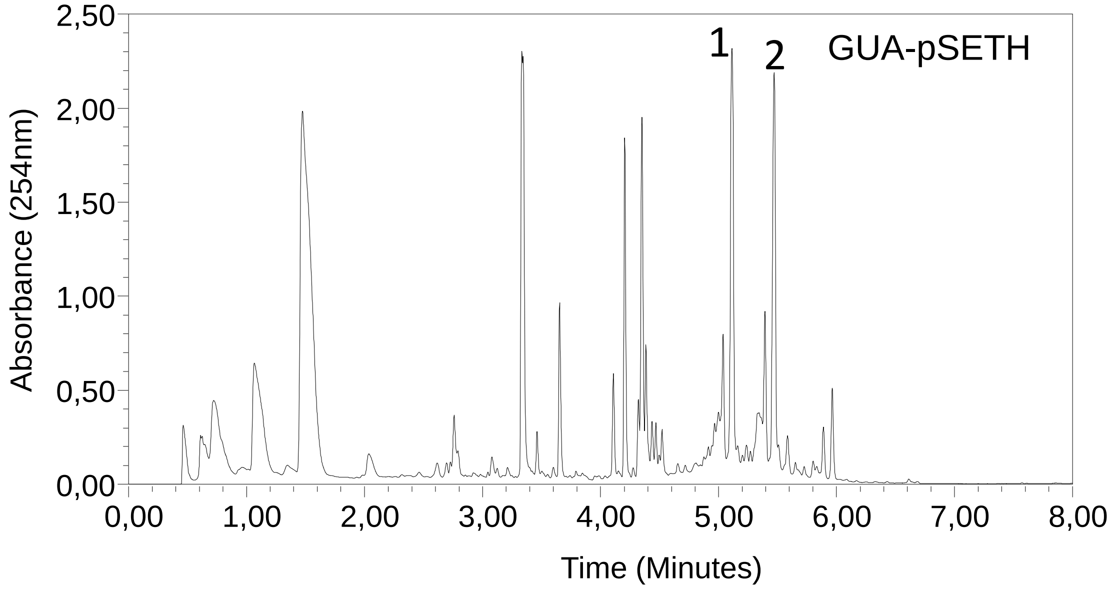

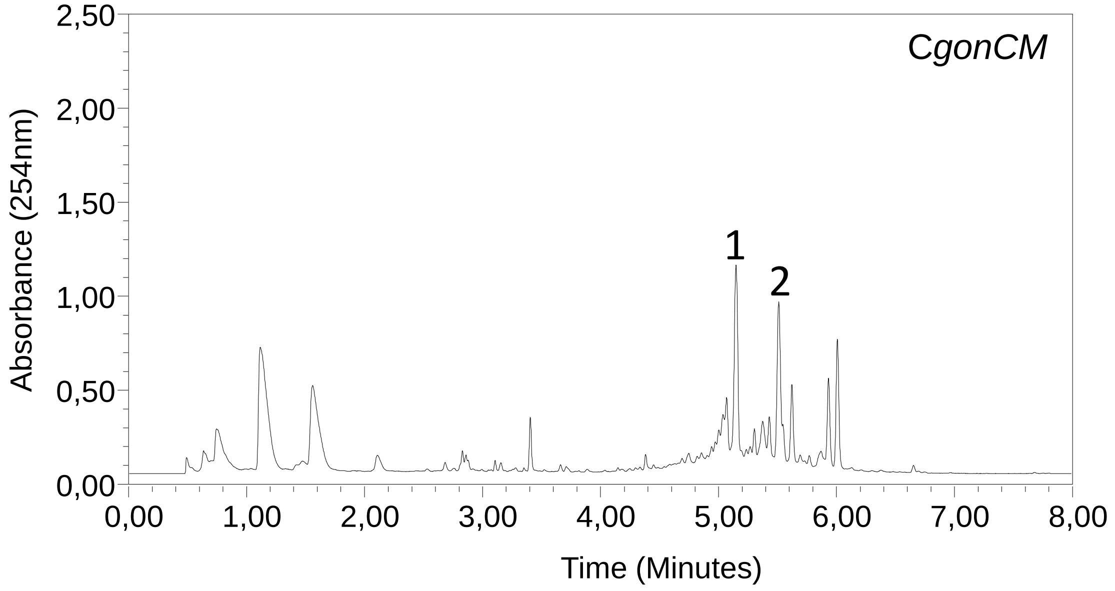

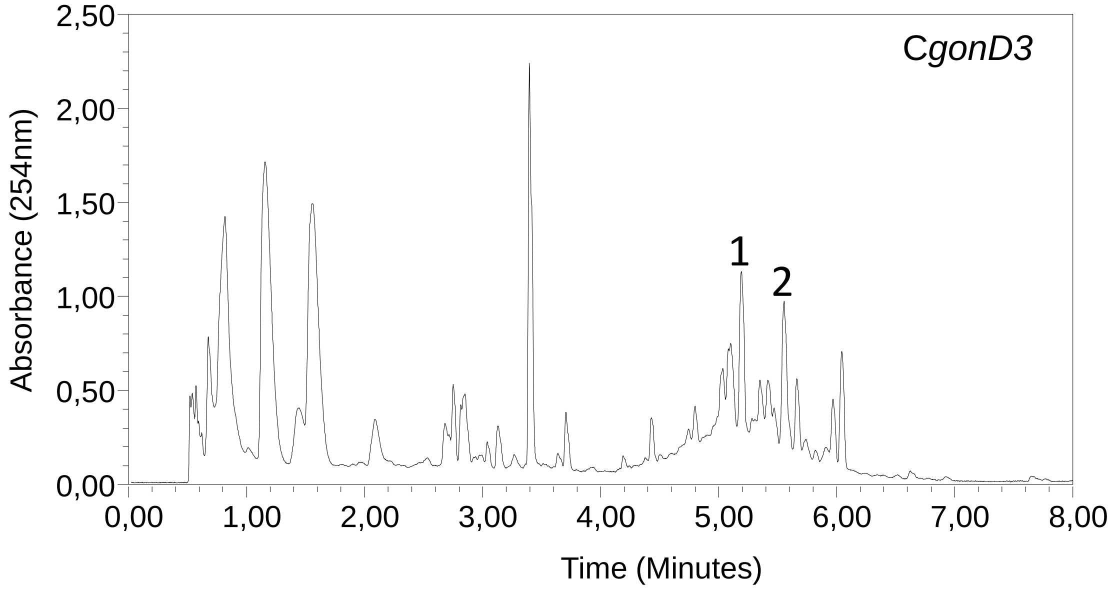
CgonD3
Absorbance (254nm)
2,50
2,00
1,50
1,00
0,50
0,00
0,00
1,00
2,00
3,00
4,00
5,00
6,00
7,00
8,00
Time (Minutes)
1
2

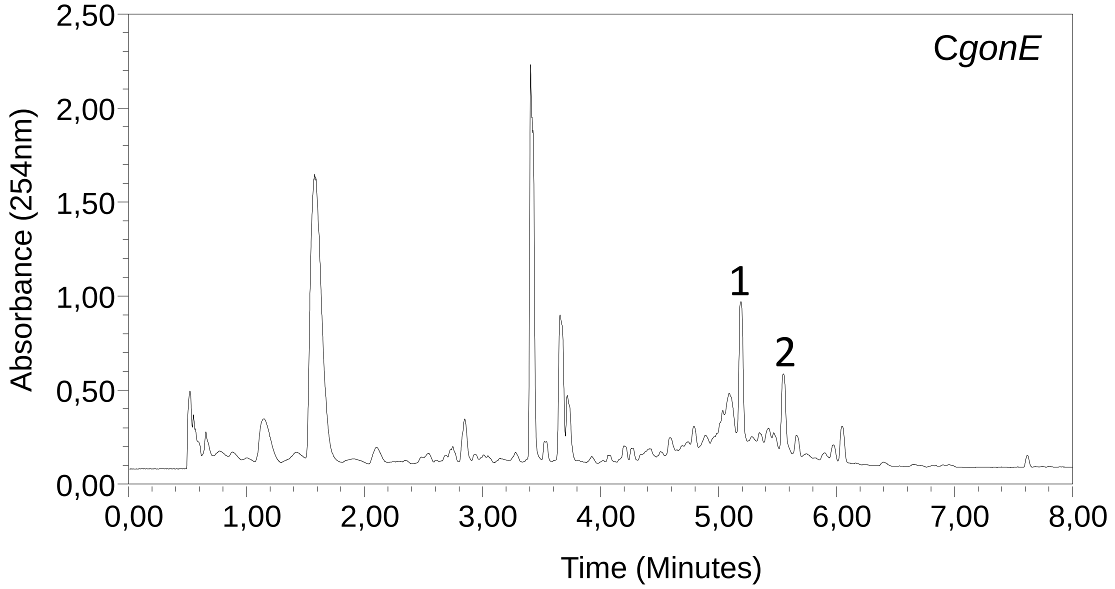
CgonE
Absorbance (254nm)
2,50
2,00
1,50
1,00
0,50
0,00
0,00
1,00
2,00
3,00
4,00
5,00
6,00
7,00
8,00
Time (Minutes)
1
2

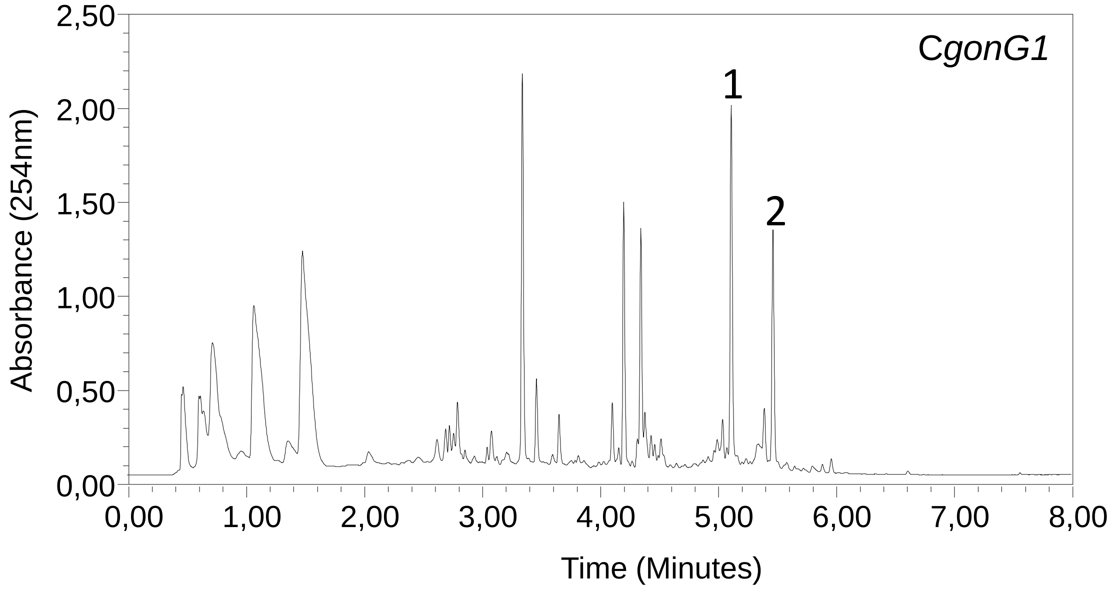
CgonG1
1
2
Absorbance (254nm)
2,50
2,00
1,50
1,00
0,50
0,00
0,00
1,00
2,00
3,00
4,00
5,00
6,00
7,00
8,00
Time (Minutes)

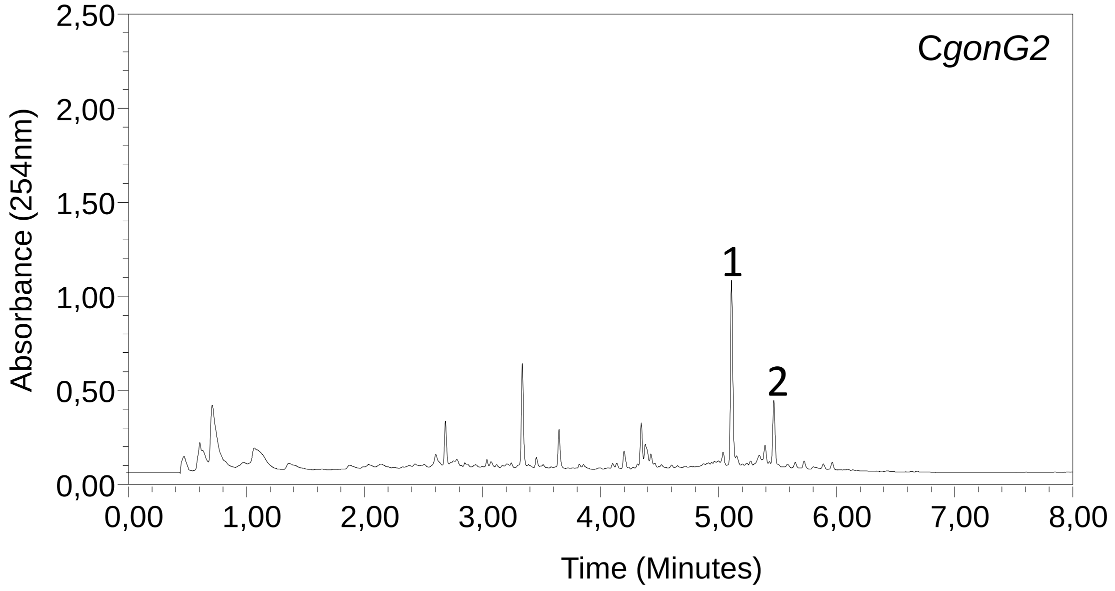
CgonG2
1
2
Absorbance (254nm)
2,50
2,00
1,50
1,00
0,50
0,00
0,00
1,00
2,00
3,00
4,00
5,00
6,00
7,00
8,00
Time (Minutes)

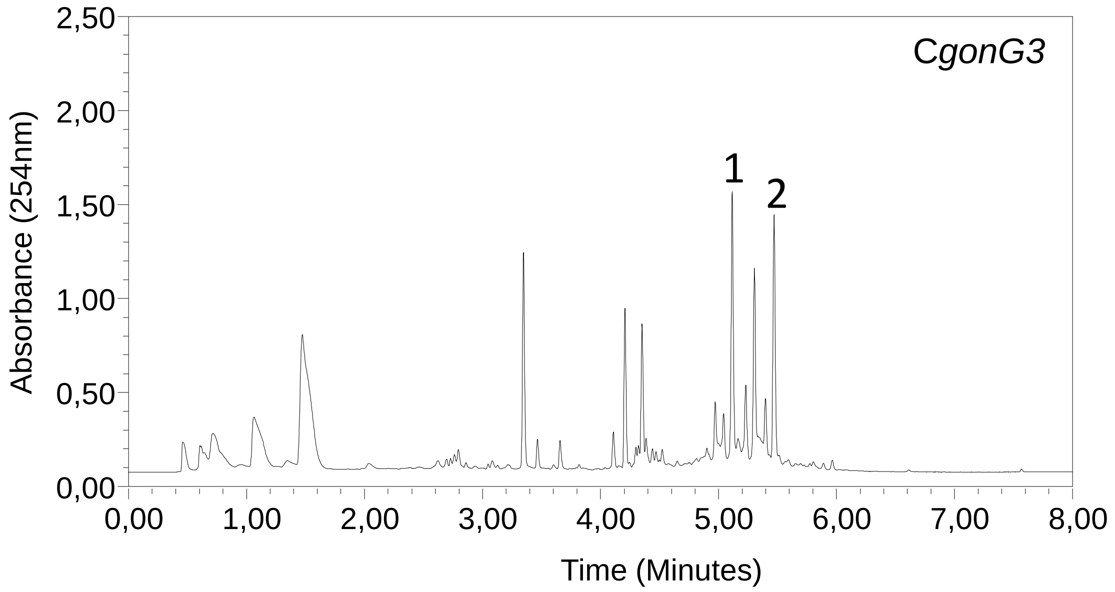

CgonG3
1
2
Absorbance (254nm)
2,50
2,00
1,50
1,00
0,50
0,00
0,00
1,00
2,00
3,00
4,00
5,00
6,00
7,00
8,00
Time (Minutes)
